# Surfactant-Mediated Buchwald–Hartwig Coupling of Aliphatic Amines for the Synthesis of DNA-Encoded Libraries

**DOI:** 10.1021/acs.bioconjchem.6c00213

**Published:** 2026-07-02

**Authors:** Thomas P. Carton, Jessica S. Graham, Michael J. Waring

**Affiliations:** Cancer Research UK Newcastle Drug Discovery Group, Chemistry, School of Natural and Environmental Sciences, Bedson Building, 5994 Newcastle University, Newcastle upon Tyne NE1 7RU, U.K.

## Abstract

New and efficient chemical reactions with broad substrate applicability are needed to expand the diversity of DNA-encoded libraries (DELs), which are becoming established as a powerful technology for hit finding in medicinal chemistry. For reactions to be useful, they must be compatible with the DNA-tags and with the aqueous conditions used in DEL synthesis. One potential approach to address this requirement is the application of surfactant-mediated chemistry, which can lead to improved reaction efficiency. Our recent application of this technology to Buchwald–Hartwig aminations is very effective at coupling (hetero)­aryl amines but performed less well for aliphatic substrates. This paper describes the development of an efficient, surfactant-promoted method of effecting on-DNA Buchwald–Hartwig aminations on DNA with aliphatic amines. This was developed through sequential optimization of the base, palladium ligand and the surfactant, for which Span 60 proved optimal. The applicability of the method is demonstrated across a broad substrate scope and its use in the synthesis of DELs is demonstrated by the synthesis of a prototype library.

## Graphical abstract

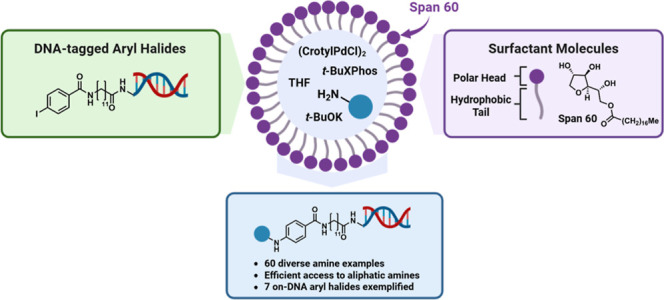

## Introduction

DNA-encoded libraries (DELs) are becoming widely applied as a technology for finding starting points for drug discovery.^1^ Their power lies in the ability to screen large numbers of compounds, each tagged with a unique DNA barcode, by affinity selection against a target protein. The DNA-tag allows identification of hits by PCR amplification of the DNA-tags of retained ligands and subsequent sequencing. To be successful, the synthesis must employ chemistry that is compatible with DNA and there is a need to increase the range of reactions that are applicable with broad substrate scope.

The amination of aryl halides is a desirable reaction for DEL synthesis due to the large number of bioactive arylamines and the diversity of commercially available amine monomers. While there are now reliable methods for Buchwald–Hartwig^2–5^ and Ullmann type^6^ amination of aryl halides with (hetero)­aryl amines, coupling of aliphatic amines is more problematic.^7^

It has been demonstrated that the use of micelle-forming surfactants^8,9^ can promote reactions on DNA-tagged substrates across a range of reaction types.^10–16^ This approach can lead to highly efficient conversions to the desired products. The application of surfactant-mediated aliphatic Buchwald–Hartwig coupling was therefore investigated.

## Results and Discussion

Investigations commenced with the application of our previously reported arylamine conditions^4^ using a 4-iodobenzamide coupled to a DNA adaptor sequence via hexylamino-PEG-4 (**HP1**) with benzylamine as the coupling partner (Scheme 1). This afforded moderate (67%) conversion to aryl benzylamine **1**. The remaining DNA-containing material was dehalogenated starting material **2**.

**Scheme 1:**
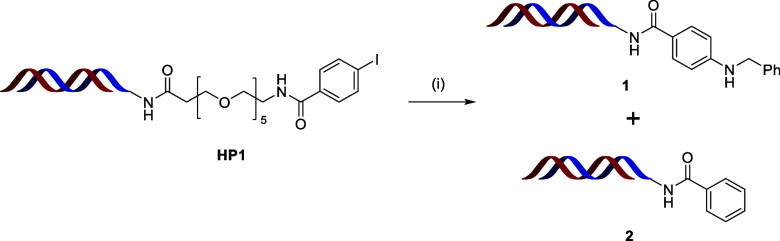
Initial Coupling to Benzylamine, (i) Conditions: **HP1** (1 nmol); Benzylamine (1.0 M), *t*-BuOK (0.2 M), [(cinnamyl)­PdCl]_2_ (3.5 M), cBRIDP (6.9 M), TPGS-750-M (3.5%), 5% DMPU, 30 μL Total Volume, 80 °C, 30 min

Investigation of the nature of the palladium source revealed that using [PdCl­(crotyl)]_2_ and BrettPhos^17^ improved the conversion for benzylamine (74%), and gave 88% for 3-phenylpropylamine and 87% for cyclohexanemethylamine, which failed to couple under previous conditions (Table 1). However, the conditions performed less well for cyclopentylmethylamine and failed for 2-pyridylmethylamine and morpholine. In all cases, dehalogenation to **2** was the only observable side-reaction.

**Table 1:**
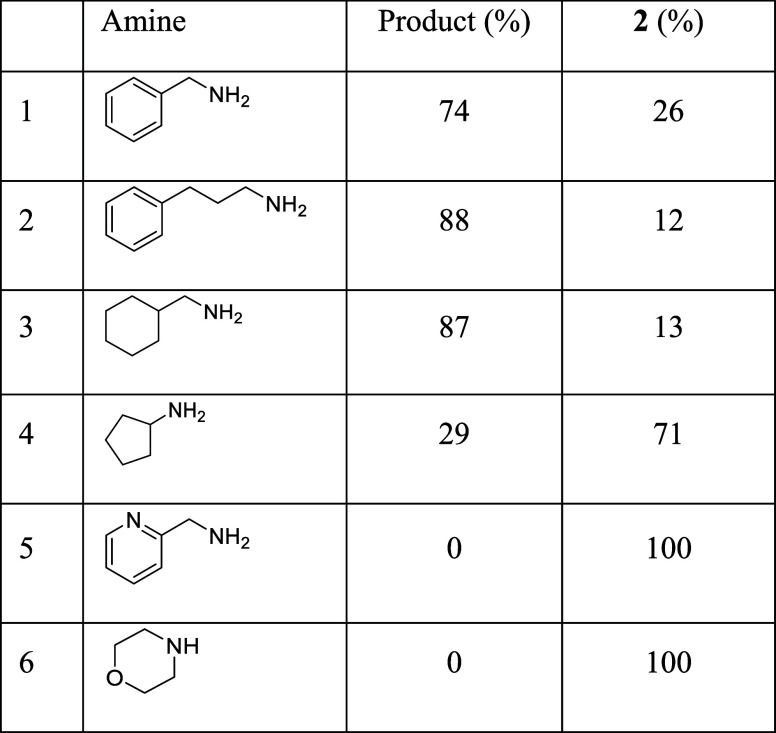
Coupling of Amines to **HP1**. Conditions: **HP1** (1 nmol), Amine (0.5 M), *t*-BuOK (0.53 M), [PdCl­(crotyl)]_2_ (10 mM), BrettPhos (20 mM), aq. TPGS-750-M aq. (2%), 30 μL Total Volume, 60 °C, 1 h

Investigation of bases and phosphine ligands followed by optimization of conditions using factorial experimental design revealed improved conversions with potassium phosphate as base and *t* **-**BuXPhos as the ligand. However, the performance across a range of substrates was still suboptimal.

Further optimization of reaction conditions was investigated through a screen of eight alternative bases (Table 2). These reactions were carried out using an alternative DNA headpiece (**HP2**), in which the DNA is conjugated to 4-iodobenzamide via a C_11_-alkyl chain linker in place of the PEG-4. These headpieces were confirmed to perform interchangeably in this case. Each base was assessed against two substrates with contrasting conversions under the established conditions: benzylamine (95% conversion) and piperidine (65%) (Table 2). Most bases screened afforded significant reductions in conversion for both amines, including a series of inorganic bases (varying both phosphate and countercation species) and Et_3_N. However, both DBU and *t*-BuOK maintained comparatively high conversions for benzylamine (88% and 84% respectively) with significant increases in conversion for piperidine relative to K_3_PO_4_ (86% and 94% respectively). An assessment of the wider scope of both these conditions to a series of 12 diverse amines highlighted the superior substrate tolerance of *t*-BuOK and K_3_PO_4_ conditions compared to DBU, both with an average conversion of 74% (Table 3).

**Table 2:**
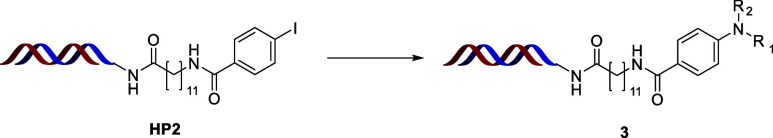

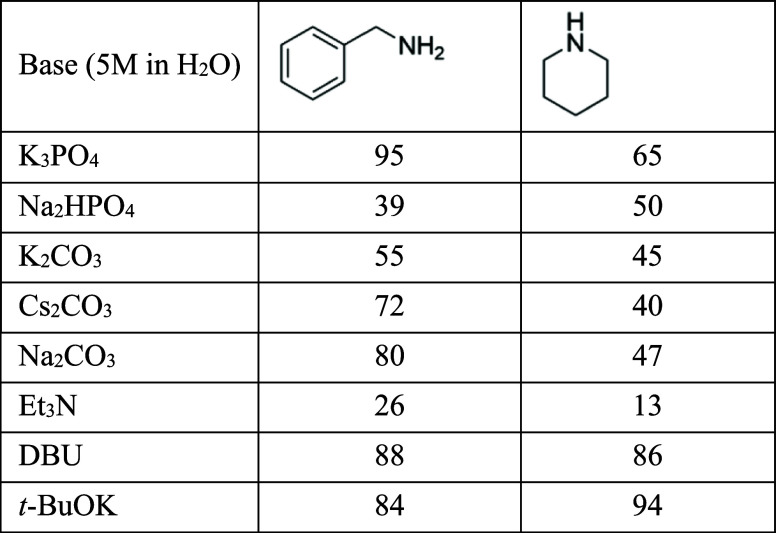
Conditions: **HP2** (1 nmol), Amine (0.3 M), Base (1 M), [PdCl­(crotyl)]_2_ (8.1 mM), tBuXPhos (16.2 mM), TPGS-750-M aq. (2%), 30 μL Total Volume, 20% THF, 70 °C, 1 h

**Table 3:**
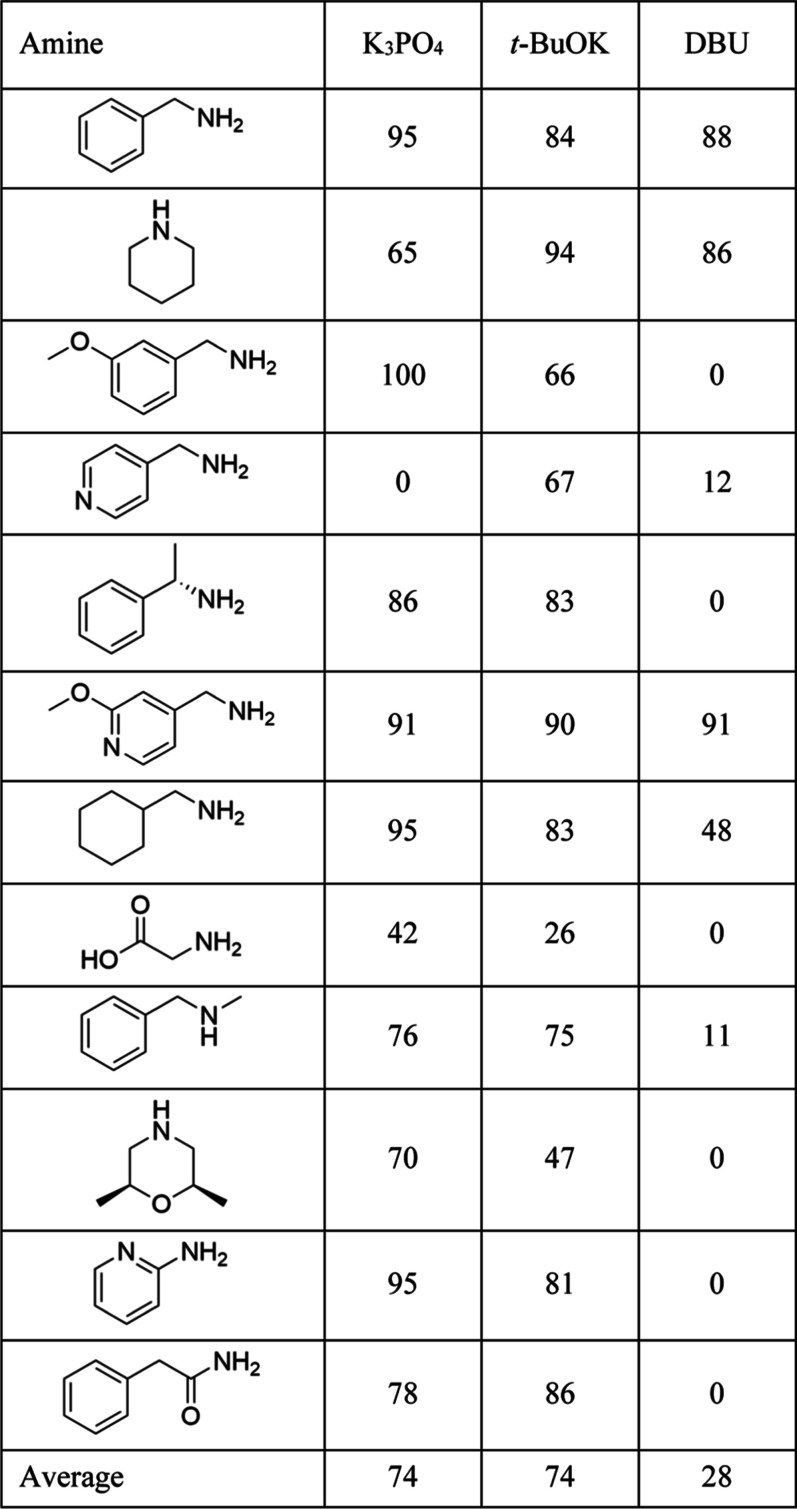
Conditions: **HP2** (1 nmol), Amine (0.3 M), Base (1 M), [PdCl­(crotyl)]_2_ (8.1 mM), *t*-BuXPhos (16.2 mM), TPGS-750-M aq. (2%), 30 μL Total Volume, 20% THF, 70 °C, 1 h

Previous work has shown that varying the surfactant employed in DEL chemistry can lead to improved conversions.^5^ A screen of 8 diverse surfactants, including zwitterionic (sulfobetaine-16), sorbitan-based (Tween 65 and Span 60), phenolic (Triton-x-405), and simple straight chain (Brij 700, PEG_5‑_C_11_ and Brij S20) surfactants was assessed (Table 4). Generally, the reaction performed well with benzylamine irrespective of surfactant choice (>75% in all cases) with BrijS20 and PEG_5_-C_11_ performing best (95% and 93% respectively). Piperidine, however, was highly variable, with TPGS-750-M and Span 60 (Figure 1) being the only surfactants affording good conversions (94% and 83% respectively).

**Table 4:**
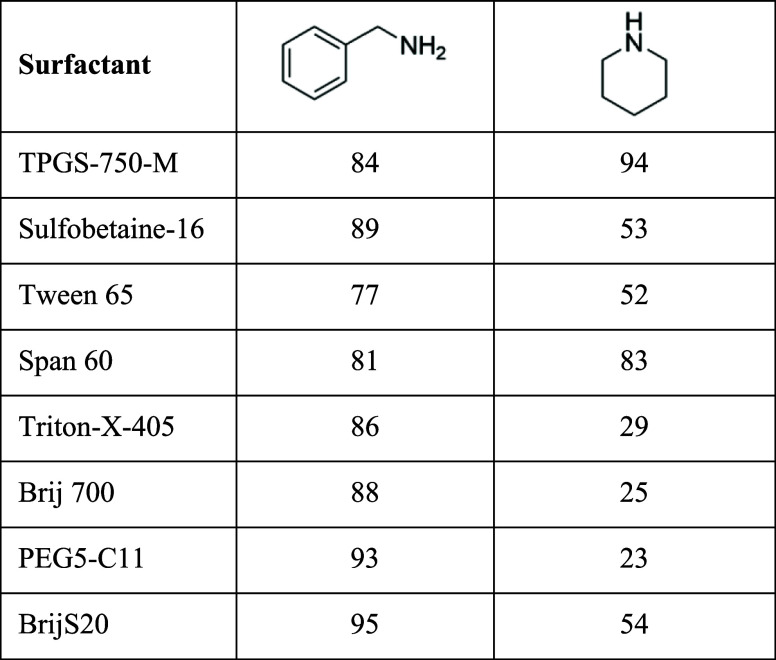
Conditions: **HP2** (1 nmol), Amine (0.3 M), *t*-BuOK (1 M), [PdCl­(crotyl)]_2_ (8.1 mM), *t*-BuXPhos (16.2 mM), Surfactant aq. (2%), 30 μL Total Volume, 20% THF, 70 °C, 1 h

**Figure 1:**
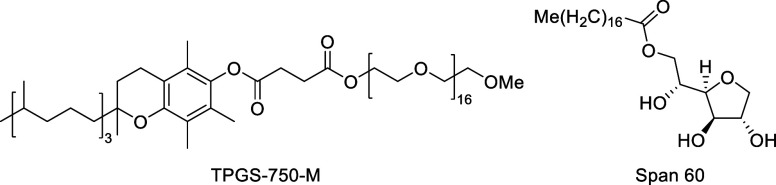
Structures of nonionic surfactants TPGS-750-M and Span 60.

An extended range of amines was subsequently assessed using Span 60 and TPGS-750-M (Table 5). An improved average conversion (78%) across the 12 substrates was observed. From this set, 11 amines reacted with over 70% conversion, with glycine the only substrate to fall below this level (potentially resulting from the poor affinity of the polar, ionised substrate for the hydrophobic surfactant). Repeating a representative set of these reactions in the absence of surfactants resulted in poorer conversions in all cases, thus establishing the beneficial effect of the surfactant on the reaction. As a result of its increased average conversion compared to TPGS-750-M (78 vs 74%) Span 60 was selected for further examination.

**Table 5:**
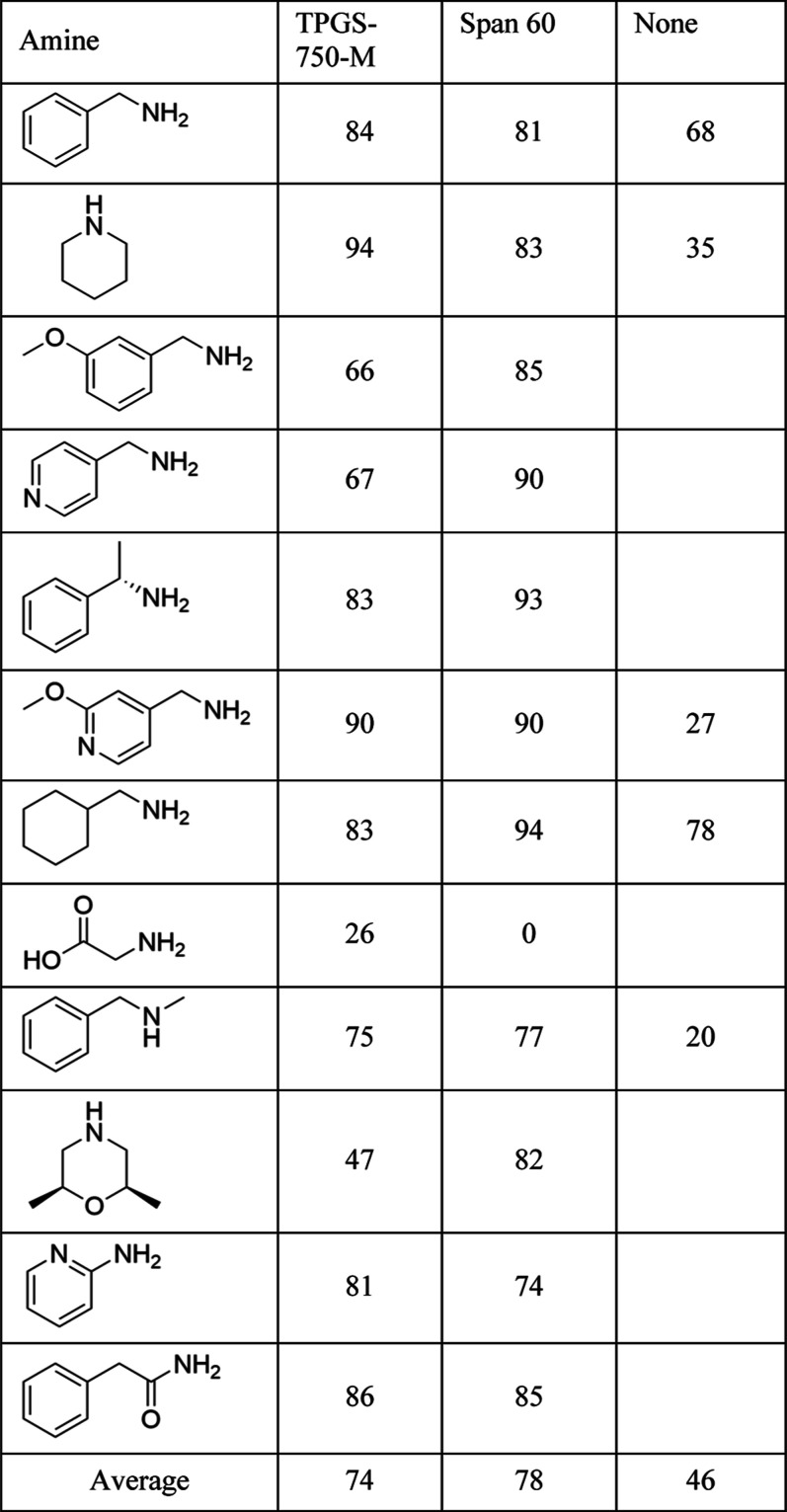
Conditions: **HP2** (1 nmol), Amine (0.3 M), *t*-BuOK (1 M), [PdCl­(crotyl)]_2_ (8.1 mM), *t*-BuXPhos (16.2 mM), Surfactant aq. (2%), 30 μL Total Volume, 20% THF, 70 °C, 1 h

While the explanation for the (albeit modest) difference in conversions between Span 60 and TPGS-750-M is unclear, it is interesting to consider factors that may give rise to the different effects. The two surfactants are relatively similar in their overall physical properties as assessed by principal components analysis.^18^ Span 60 is a more lipophilic and less water-soluble surfactant than TPGS-750-M and their propensity to form micelles or other structures such as niosomes in aqueous media differ. While it is unknown where the reaction occurs in these heterogeneous systems, it is possible that the environment is very different between the two systems.

The optimized conditions with Span 60 were assessed over an extended scope of 61 substrates, including both aliphatic and aromatic amines (Table 6). 32 of the amines reacted with >70% conversion to the desired product, a further 11 afforded conversions >50%, while 18 fell below 50% conversion. A wide range of aryl-containing amines performed with high conversions, including substituted aryls such as 3-methoxybenzylamine (85%) and 4-methylbenzylamine (76%). Substituents on the methylene linker position e.g. (+)-phenylethylamine (93%) and 1-(4-fluorophenyl)­ethylamine (70%) were tolerated. The incorporation of heteroaromatics was also favorable, with 4-(aminomethyl)­pyridine (90%) and 2-thiophenemethylamine (92%) proceeding efficiently. Simple aliphatic systems also afforded high conversions, including cyclohexanemethylamine (94%) and cyclohexanamine (78%). However, simple aliphatic *N*-methylamines generally gave poorer conversions, e.g. *N*-methylcyclohexanamine (7%). The most probable cause of this is the increased steric hindrance in these substrates. Introduction of additional heteroatoms, in particular basic nitrogens, resulted in significantly reduced conversion, potentially resulting from chelation of these substrates with the Pd center. However, despite being optimized for aliphatic amines, a set of aromatic amines afforded consistently high conversions (6 out of 8 proceeded with conversion >70%), establishing these conditions as a broad protocol for both aliphatic and aromatic amines. Finally, several functionally diverse substrates, including a primary amide (85%) and two ureas (78% and 77%), proceeded with excellent conversions.

**Table 6:**
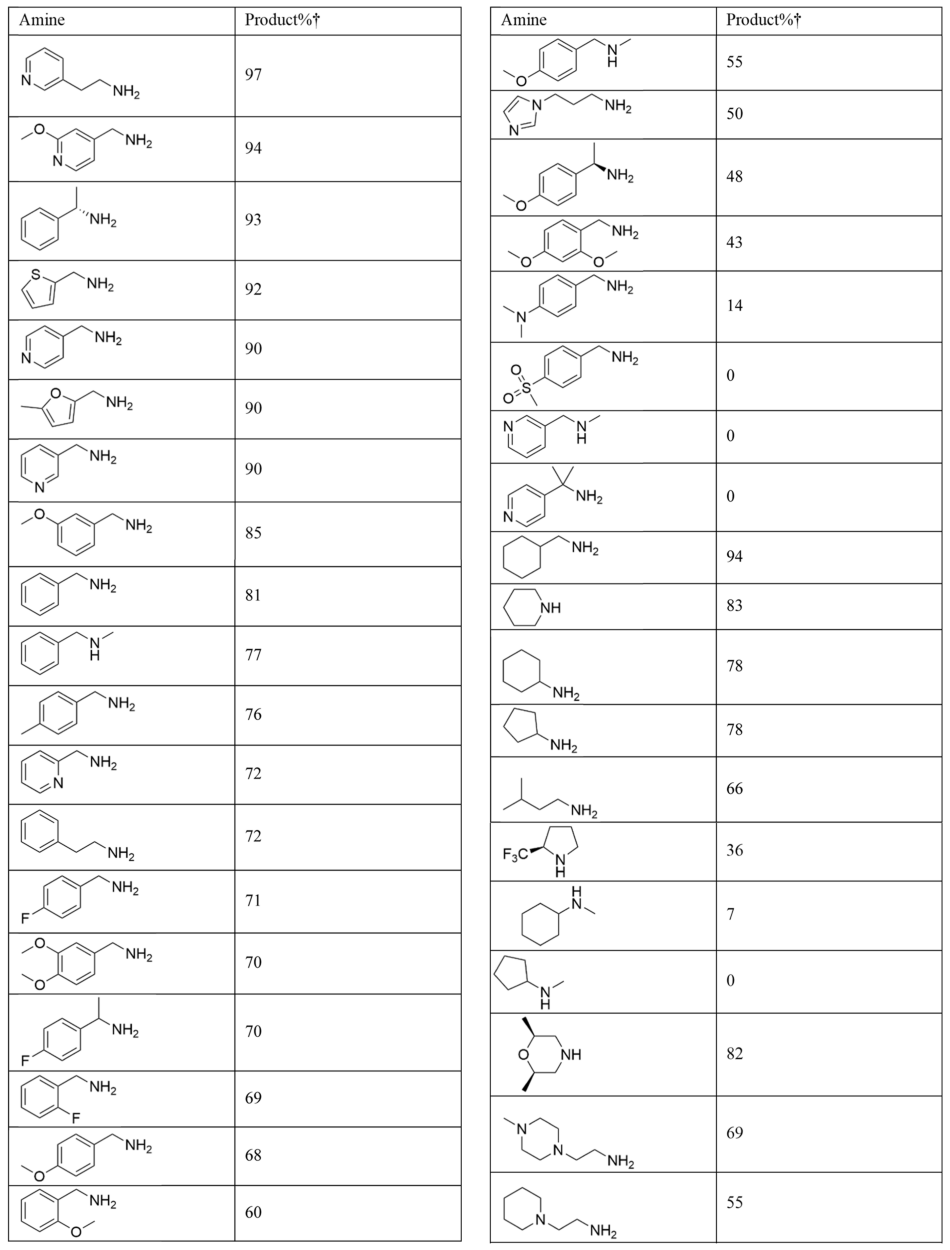

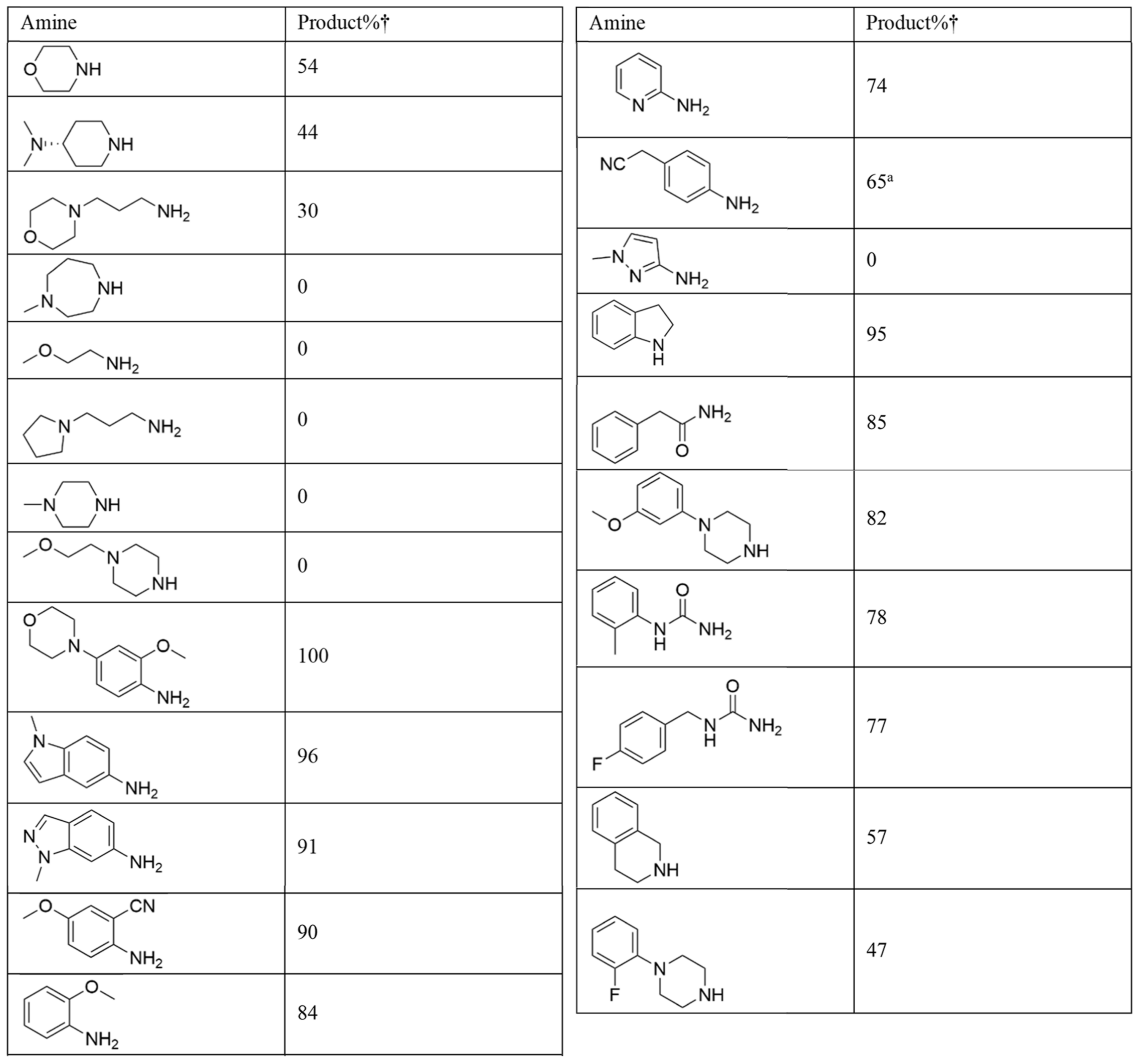

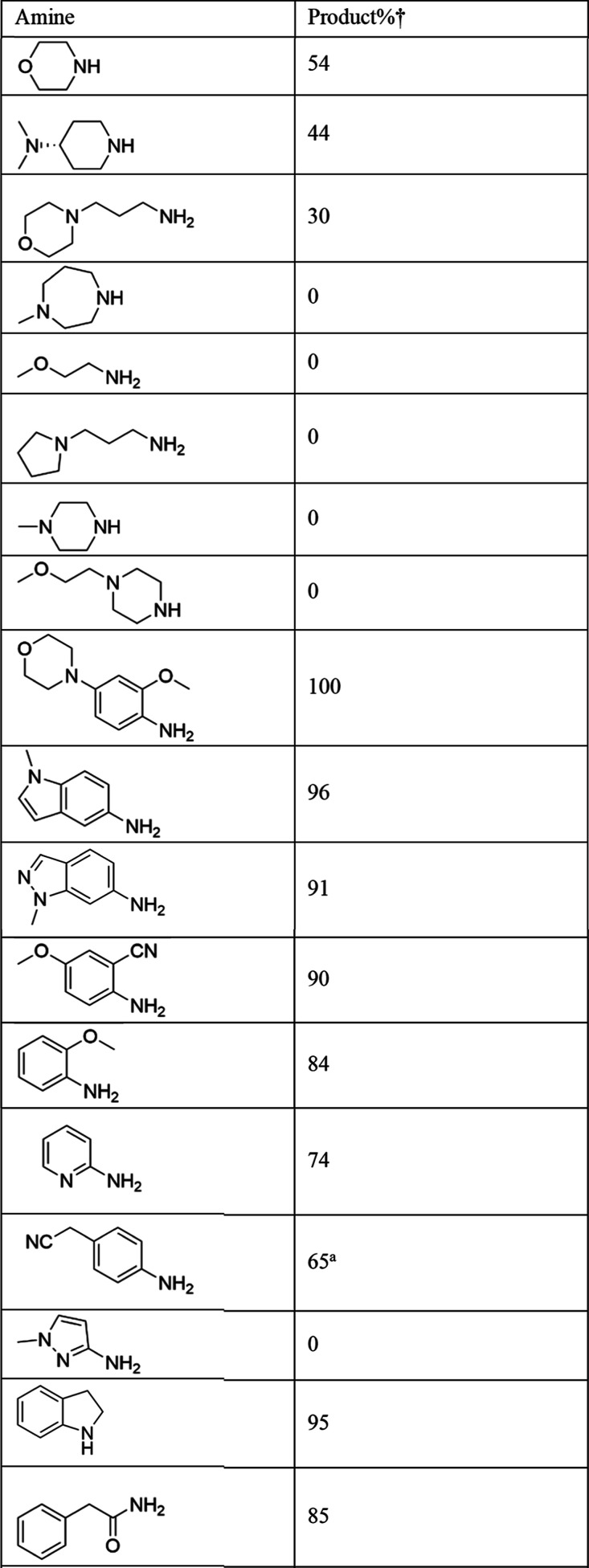

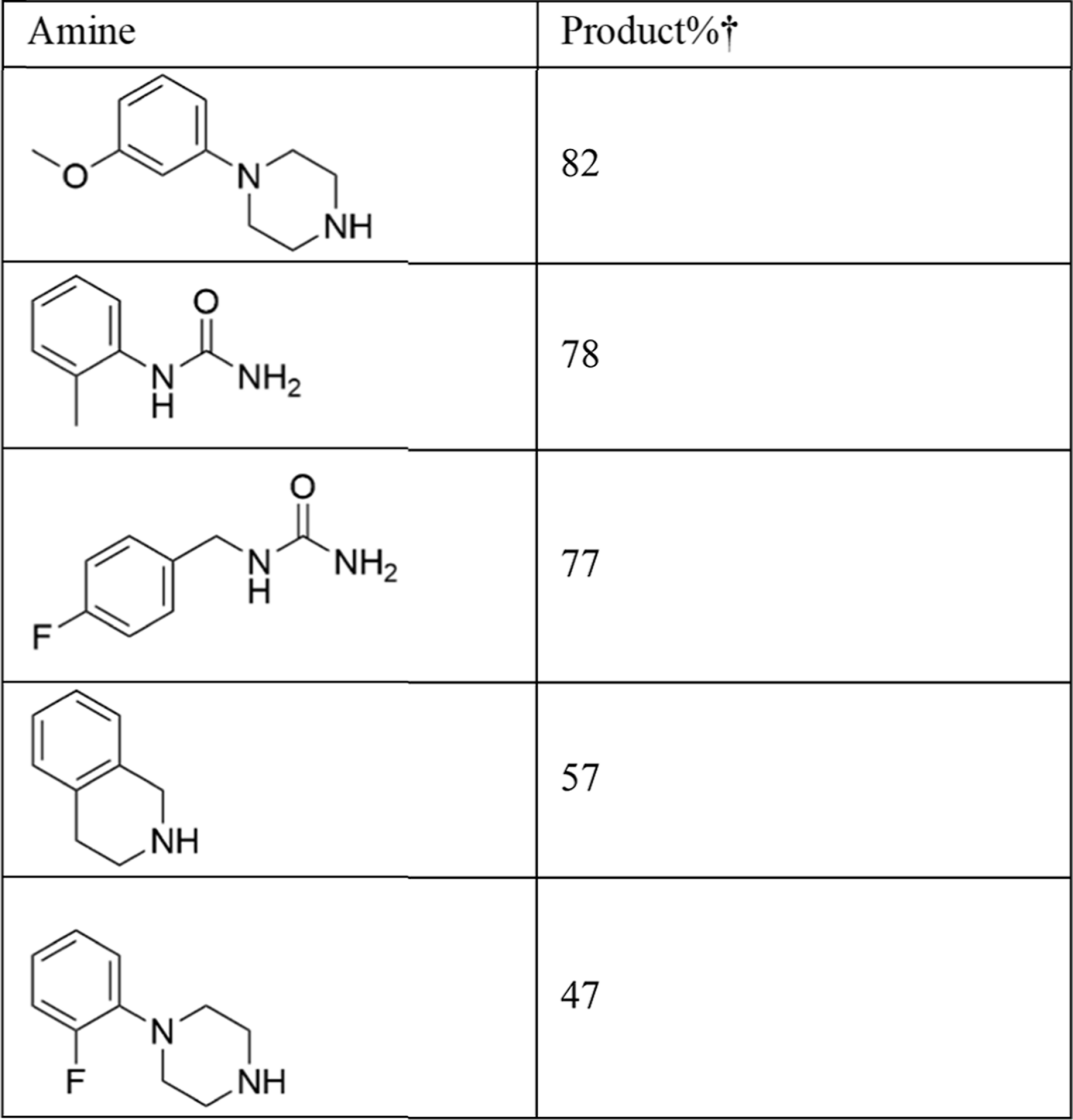
Conditions: **HP2** (1 nmol), Amine (0.3 M), *t*-BuOK (1 M), [PdCl­(crotyl)]_2_ (8.1 mM), *t*-BuXPhos (16.2 mM), Span 60 (2%), 30 μL Total Volume, 20% THF, 70 °C, 1 h

† Remaining DNA products are dehalogenated or hydroxylated aryl headpiece side-products.

a Product includes hydrolyzed amide.

In comparison with existing approaches toward DEL-compatible aliphatic amine Buchwald–Hartwig coupling, this method affords superior conversions. The 61 amines demonstrated increased average conversions compared to the approach reported by Chheda et al. (59% vs 52%).^7^ Furthermore, with this method, 46% of the aliphatic amines proceeded with conversion >70% while this alternative approach only reached this threshold with 26% of the aliphatic amines exemplified.^7^ In addition, although a related Ullmann coupling approach has demonstrated comparable conversions for aliphatic amines, the use of Cu-promoted methodology has been demonstrated to afford damage to the DNA tag, making our Pd-based approach a highly attractive alternative.^6^

Having established the diverse amine scope, the on-DNA aryl halide compatibility was investigated. A series of 5 amines, which afforded a range of conversions against the DNA tagged 4-iodobenzamide headpiece, were assessed against 6 alternative aryl halides (Table 7). Generally, good conversions were observed across the series, particularly with the 4-bromobenzamide headpiece, where 4 of the 5 amines afforded excellent conversion (>90%). A pyridine (5-iodopicolinate) was poorly tolerated, however, with only benzylamine and cyclohexanemethylamine proceeding with moderate conversions (46% and 49% respectively). Both a 3-iodobenzamide and 4-iodophenylacetamide headpieces proceeded with high efficiency across the rage of amines, highlighting the tolerance of varied electronics, with both examples of halogen substitution in positions less activated to oxidative addition. Substitution of the aryl halide was also shown to be tolerated including both 5-bromo-2-trifluoromethylbenzamide and 4-bromo-2-fluorobenzamide with moderate to excellent conversions afforded across the 5 amines.

**Table 7:**
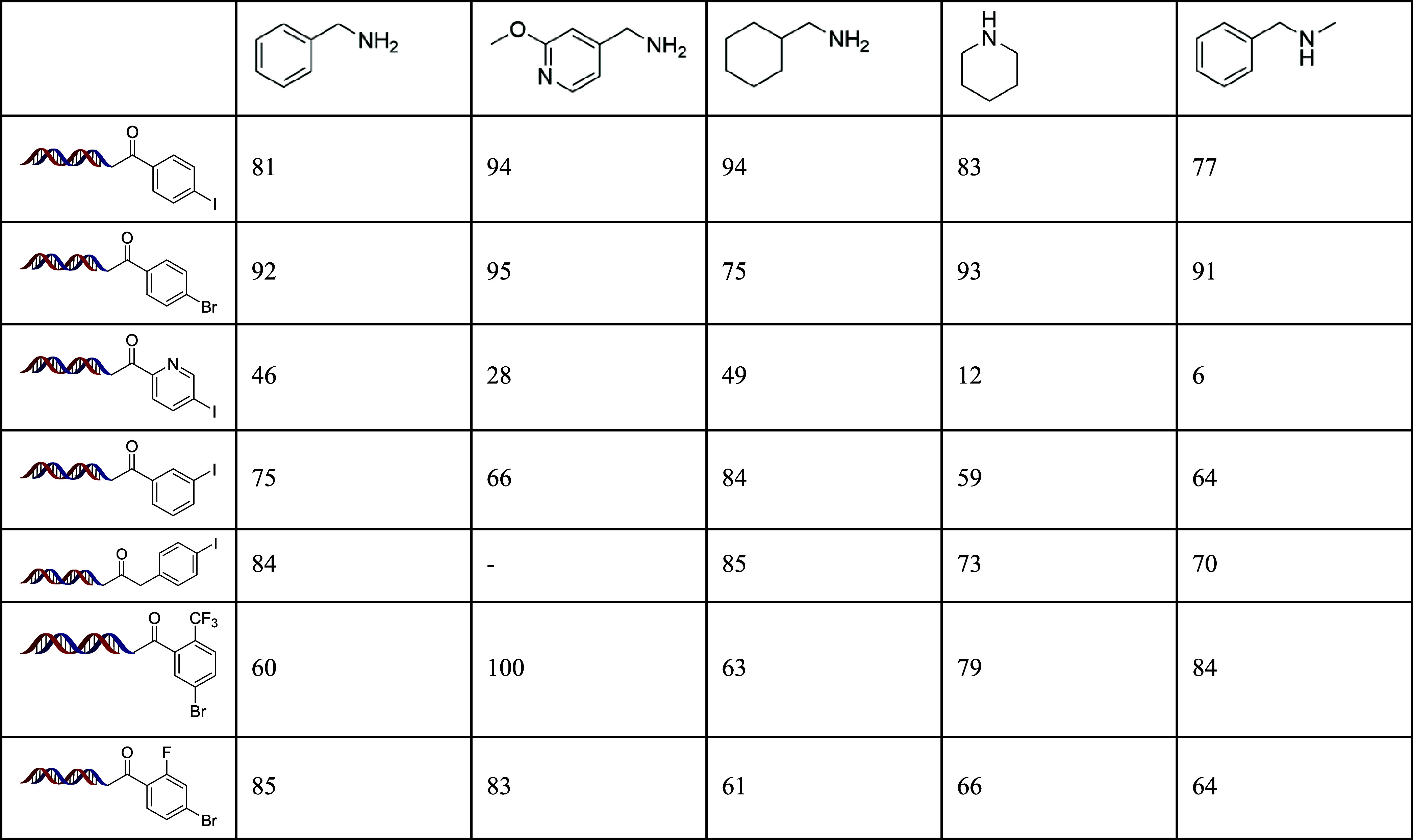
Conditions: **HP2** (1 nmol), Amine (0.3 M), *t*-BuOK (1 M), [PdCl­(crotyl)]_2_ (8.1 mM), *t*-BuXPhos (16.2 mM), Span 60 (2%), 30 μL Total Volume, 20% THF, 70 °C, 1 h

To highlight the applicability of the methodology to DEL synthesis, a 2 × 2 DNA-encoded library was synthesized, incorporating a Buchwald–Hartwig coupling step using the optimized conditions (Scheme 2). The library was generated through an initial amide coupling step of aryl halide-containing acids, 4-iodobenzoic acid and 4-bromo-2-fluorobenzoic acid to an amino-tagged DNA headpiece HP2, followed by ligation of a specific DNA codon sequence. The DNA products were pooled and split into two wells, where either benzylamine or (2-methoxypyridin-4-yl)­methanamine was subsequently coupled with the resulting on-DNA aryl halides and a second ligation of the second codon and closing primer sequence. DNA ligation was confirmed to have occurred efficiently by the appearance of a single band upon gel electrophoresis (Figure S92). PCR amplification (40 cycles) of the pooled library with next-generation sequencing (NGS) elongation primers resulted in a major band of the expected 161 base pair length (Figure S93), suggesting efficient amplification of the DNA barcode following exposure to the reaction conditions. NGS of the amplified library confirmed the conserved integrity of the DNA barcode, with 68% of the 42,222 reads corresponding to the expected sequences.

**Scheme 2:**
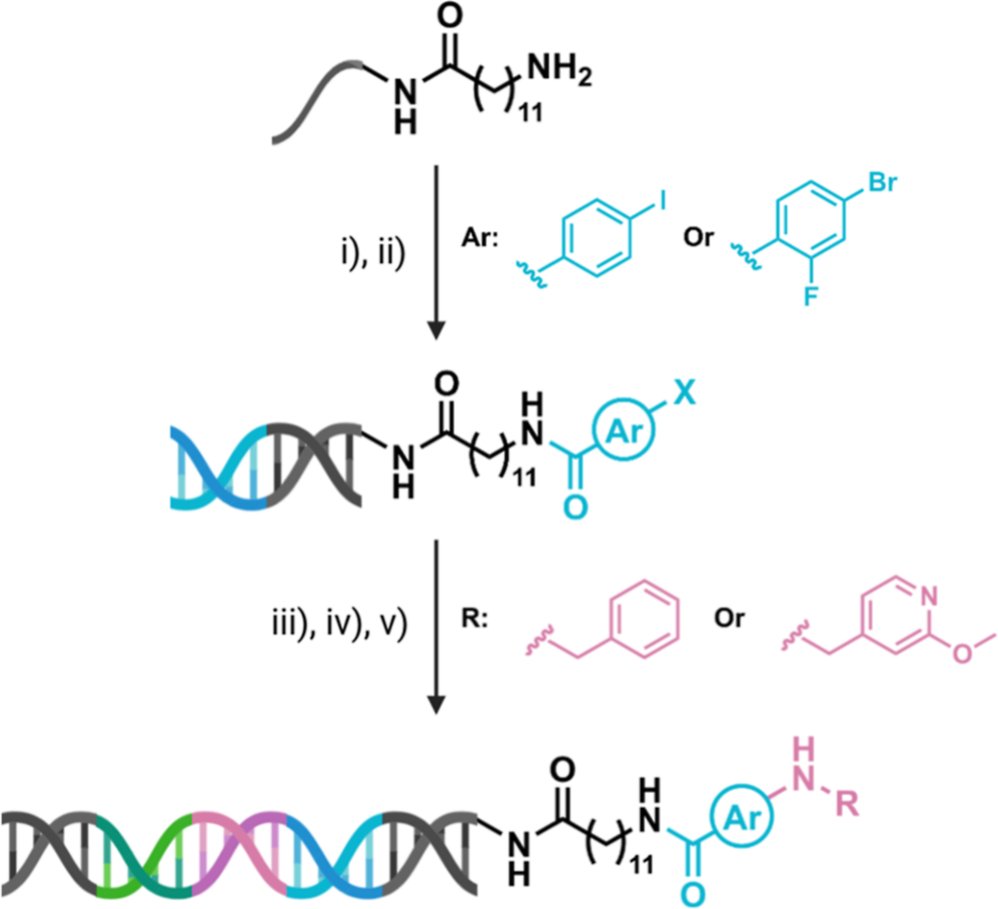
Synthesis of a 2 × 2 Prototype DEL Using the Optimized Buchwald–Hartwig Coupling in the Second Step. Conditions: (i) Phosphorylation (Primer, Complementary Primer, First Building Block Oligo), Ligation (Phosphorylated DNA, Headpiece Oligo, First Building Block Complementary Oligo); (ii) Carboxylic Acid (28 mM), EDC.HCl (14 mM), Sulfo-NHS (14 mM), DMSO (37%), MOPS (58 mM, pH8), 37 °C, 16 h.; (iii) Phosphorylation (Library Oligo, Second Building Block), Ligation (Phosphorylated DNA, Complementary Second Building Block); (iv) HP1 (1 nmol), Amine (0.3 M), *t*-BuOK (1 M), [PdCl­(crotyl)]_2_ (8.1 mM), *t*-BuXPhos (16.2 mM), Span 60 (2%), 30 μL Total Volume, 20% THF, 70 °C, 1 h. V) Phosphorylation (Library Oligo, Third Building Block + Complementary Reverse Primer Oligo), Ligation (Phosphorylated DNA, Complementary Third Building Block + Reverse Primer Oligo). Created with BioRender.com

## Conclusion

In summary, we have developed a surfactant-mediated Buchwald–Hartwig coupling protocol that enables efficient on-DNA amination of aryl halides with a broad range of aliphatic amines and is also compatible with (hetero)­aryl amines. Through evaluation of palladium sources, ligands, bases, and surfactants, Span 60 and *t*-BuOK were identified as key components in achieving improved substrate tolerance and consistently high conversions. The optimized conditions proved effective across a diverse set of 60 amines and were successfully applied in the construction of a small DNA-encoded library, with preservation of DNA integrity. This work expands the scope of DNA-compatible C–N bond-forming reactions and provides a robust, broadly applicable methodology for the incorporation of structurally diverse amines into DNA-encoded libraries, thereby enhancing the chemical space accessible for DEL-based drug discovery.

## Supplementary Material



## Notes

Additional data, experimental details and supporting analytical data are available as electronic Supporting Information (PDF)

## †

JSG, The Francis Crick Institute, 1 Midland Road, London, NW1 1AT, UK

## Abbreviations

DEL: DNA-encoded library
DNA: Deoxyribonucleic acid
HP: head piece
NGS: next-generation sequencing
PCR: polymerase chain reaction.
