# Supplementary material for: Surfactant-Mediated Buchwald–Hartwig Coupling of Aliphatic Amines for the Synthesis of DNA-Encoded Libraries
Source: Bioconjug Chem. 2026 Jul 2;37(7):1443–51. doi: 10.1021/acs.bioconjchem.6c00213 (PMC13377609; doi:10.1021/acs.bioconjchem.6c00213)
Supplement: Supplementary file 1 [file bc6c00213_si_001.pdf]

Supporting Information

Surfactant-mediated Buchwald-Hartwig coupling of aliphatic amines for the synthesis of DNA-encoded libraries

*Thomas P. Carton,[a] Jessica S. Graham[a] and Michael J. Waring.[a]**

[a]Cancer Research UK Newcastle Drug Discovery Group, Chemistry, School of Natural and Environmental Sciences, Bedson Building, Newcastle University, Newcastle upon Tyne, NE1 7RU, UK.

*Email: mike.waring@ncl.ac.uk.

Table of Contents

Materials and Methods **3**

Solvents and Reagents 3

Analytical Techniques 3

Chromatography and Equipment 4

General Procedures **4**

General DNA Headpiece Synthesis Procedure 4

General Ethanol Precipitation Procedure for DNA Purification 5

General on-DNA DMT-MM Forward Amide Coupling Procedure 5

On-DNA Buchwald-Hartwig Amination with DNA-Conjugated Aryl-iodo HP2 ..... **6**

Synthesis and Characterisation of DNA Headpiece HP2 6

General on-DNA Aliphatic Buchwald-Hartwig Amination Procedure 7

Buchwald-Hartwig Amination (Table 6) Chromatograms 8

On-DNA Buchwald-Hartwig Amination with DNA-Conjugated Aryl Halides (Table 7) **35**

Synthesis and Characterisation of DNA Headpieces for Table 7 35

6x6 Buchwald-Hartwig Amination (Table 7) Chromatograms 39

DNA-Encoded Library Synthesis **55**

2x2 Library Construction 55

Cycle 1 56

Cycle 2 57

PCR and Sequencing 58

Materials and Methods

Safety

Prior to commencing practical work COSHH risk assessments were completed and no unexpected or unusually high safety hazards were encountered.

Solvents and Reagents

Reagents were purchased from Acros Organics, Alfa Aesar, Apollo Scientific, BLD Pharmatech, Fluorochem, Manchester Organics, Sigma-Aldrich; linkers from Apollo Scientific and Sigma-Aldrich; and surfactants from Sigma-Aldrich. All chemicals were used without further purification. Bottles of anhydrous solvents using SureSeal™ were purchased from Sigma-Aldrich. All water used alongside DNA substrates was nuclease-free, DEPC-treated water purchased from ThermoFisher. Solid-supported 14mer DNA and the corresponding complementary strand were custom synthesised by Sigma-Aldrich and supplied as solid-supported crude material and single-stranded DNA after desalting, respectively.

Analytical Techniques

DNA mass spectrometry was conducted on an Agilent 6550 QTOF in negative mode, using a standard 3200 m/z maximum and a 2 GHz extended dynamic range. Drying gas temperature was 260 °C at 12 L/min, sheath gas temperature was 400 °C at 12 L/min, nebulizer at 45 psig, VCap voltage of 4000 V, and nozzle voltage of 2000 V. The LC was carried out on an Agilent 1260 Infinity 2 using an Agilent Advancedbio oligonucleotides column, 2.1x100 mm, where the gradient was run at either:

A) 0.45 mL/min from 40–70% MeOH over 4.5 mins against a 25 mM HFIP:15 mM hexylamine buffer solution.

B) 0.8 mL/min from 20–70% MeOH over 3.5 mins against a 200 mM HFIP:8 mM triethylamine buffer solution.

C) 0.8 mL/min from 10–50% MeOH over 3.5 mins against a 200 mM HFIP:8 mM triethylamine buffer solution.

D) 0.4 mL/min from 10–40% MeOH over 10 mins against a 200 mM HFIP:8 mM triethylamine buffer solution.

All methods were followed by a 1.5 min flush at 95% MeOH.

Analysis of data was carried out by Agilent Qualitative Analysis version 7.

Calculated exact masses were quoted from ChemDraw Professional 18.1. Conversions were determined by integrating peaks in TIC chromatograms for the starting material and all products. The proportion of the desired product was determined by integrating all peaks, including the starting

material. Both conversion and the desired product were reported as a percentage of all components detected.

DNA concentrations were calculated using a NanoDrop™ One/OneC Microvolume UV-Vis Spectrophotometer with a 1 µL sample loading.

Gel electrophoresis was conducted using prepacked E-Gel® EX 4% Agarose Gels on an Invitrogen™ E-Gel™ Power Snap Electrophoresis Device, using Invitrogen™ E-Gel™ Ultra Low Range DNA Ladders.

Chromatography and Equipment

Preparative HPLC purification was undertaken on an Agilent 1260 Infinity system using a Phenomenex Clarity 5 µm Oligo-RP column, 10x150 mm. The gradient was run at 5 mL/min from 10–95% MeOH over 20 mins against a 50 mM HFIP:15 mM DIPEA buffer solution. Fractions were analysed at 254 nm wavelength.

Ligations and phosphorylations were carried out using an Applied Biosystems™ ProFlex™ PCR System, 96-well.

Unless otherwise specified, centrifugation was undertaken at 13,400 rpm using an Eppendorf™ Centrifuge MiniSpin®, non-refrigerated, except for ethanol precipitations, which were undertaken at 15,000 rpm using an Eppendorf™ Centrifuge 5424 R at 4 °C.

General Procedures

General DNA Headpiece Synthesis Procedure

Scheme S1 Summary of the synthesis of double-stranded 14-mer DNA headpieces from polymer-bound single-stranded MMT-protected DNA, comprising the multiple steps outlined below.

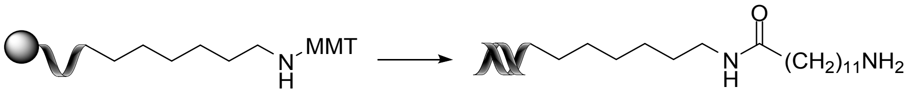

MMT Deprotection: The average loading of single-stranded DNA attached to solid support was found by cleavage from the solid support using the below method and repeating 3 times. Nanodrop concentration of cleaved DNA showed that 103 mg resulted in 2 µmol of DNA. The single-stranded DNA used was a 14mer (GTCTTGCCGAATTC) with a 5’ MMT amino C6 linker bound to solid support at the 3’ end.

The solid-supported DNA (103 mg, ca. 2 µmol) was washed with 5% TCA in DCM (20 x 400 µL). Successful removal of the protecting group was complete once the yellow colour subsided, and the solid-supported DNA was subsequently washed with DCM (14 x 400 µL). The beads were allowed to air dry (2 h) before being carried through to the next step without further analysis or purification.

Linker Coupling to Headpiece: To a centrifuge tube was added HATU (17 mg, 44 µmol); 12-((((9*H*-fluoren-9-yl)methoxy)carbonyl)amino)dodecanoic acid (18 mg, 40 µmol); and DIPEA (17 µL, 100 µmol) in DMF (1 mL). The mixture was vortexed at room temperature for 15 mins. The deprotected, solid-

supported DNA was added (ca. 2 μmol) and the mixture vortexed at room temperature for 20 h. The reaction mixture was filtered and washed with DMF (3 x 500 μL), MeCN (3 x 500 μL), MeOH (3 x 500 μL), and DCM (3 x 500 μL). The beads were allowed to air dry (1 h) before being carried through to the next step without analysis.

Cleavage from Solid Support: 40 wt.% aqueous methylamine solution and 33% aqueous NH_3 solution were added to a centrifuge tube and vortexed at room temperature for 15 mins. The solid-supported, linker-bound DNA was added and the reaction mixture was vortexed at room temperature for 5 h. The mixture was filtered and washed with DEPC-treated water (3 x 500 μL), before being concentrated under N_2 at rt. The product was purified via preparative HPLC (10–90% MeOH over 16 mins against a 50 mM HFIP: 15 mM DIPEA buffer solution) to afford the desired compound as a white solid.

Complementary Strand Annealing: The complementary 14mer (GAATTCGGCAAGAC), 3' and 5' hydroxylated (1.0 mM in H_2O, 1 eq), and linker-bound DNA (1 eq) were heated to 80 °C for 30 mins. The reaction mixture was allowed to cool to room temperature, and the double-stranded DNA concentrated under N_2 at rt to form a 1–4 mM solution of **greasy amine DNA HP**. Mass spectrum analysis confirmed product formation.

General Ethanol Precipitation Procedure for DNA Purification

To the reaction mixture was added 10% volume NaCl (5 M in water) and 3x volume cold EtOH. The mixture was either incubated for 2 hours at -78 °C, or overnight at -20 °C. The sample was then centrifuged at 13400 rpm at 4 °C for 15 minutes. The supernatant was decanted, and cold 70% EtOH was added. The mixture was centrifuged at 13400 rpm at 4°C for a further 10 minutes and the supernatant was again decanted. The resulting pellet was allowed to air dry before being redissolved in water.

General on-DNA DMT-MM Forward Amide Coupling Procedure

To a solution of double-stranded DNA (50 μL, 0.5–10 nmol in 150 mM borate buffer pH 9.3) was added carboxylic acid (12.6 μL, 150 mM in DMF), followed by DMT-MM (7.6 μL, 250 mM in H_2O). The reaction mixture was shaken at room temperature for 16 h before extra DMT-MM (7.6 μL, 250 mM in H_2O) was added and the reaction shaken at room temperature for a further 2 h. The reaction was then precipitated according to the general ethanol precipitation procedure and analysed by mass spectrometry.

General on-DNA EDC/Sulfo-NHS Forward Amide Coupling Procedure

To a solution of desired carboxylic acid (12 μL, 200 mM in DMSO), DMSO (50 μL), EDC in DMSO (12 μL, 100 mM DMSO), and *N*-hydroxysulfosuccinimide (12 μL, 100 mM in 2:1 DMSO/H_2O) were added to a 1.5 mL DNA LoBind® Tube and left to shake for 30 mins. Double-stranded DNA (5-10 nmol) was dissolved in pH 8 MOPS buffer (100 mM, 50 μL) and added to the reaction mixture. The reaction mixture was then heated to 37 °C in a sand bath for 16 h. The reaction was then precipitated according to the general ethanol precipitation procedure and analysed by mass spectrometry.

On-DNA Buchwald-Hartwig Amination with DNA-Conjugated Aryl-iodo HP2

Synthesis and Characterisation of DNA Headpiece HP2

DNA headpiece **HP2** was prepared utilising the general DNA headpiece synthesis procedure, followed by amide coupling with 4-iodobenzoic acid according to the general on-DNA DMT-MM Forward Amide Coupling Procedure.

Figure S1 Chromatogram displaying double-stranded **greasy amine DNA HP** starting material – used for synthesis of aryl halide DNA headpieces for Buchwald-Hartwig aminations, comprising both the complementary strand and DNA-greasy amine linker.

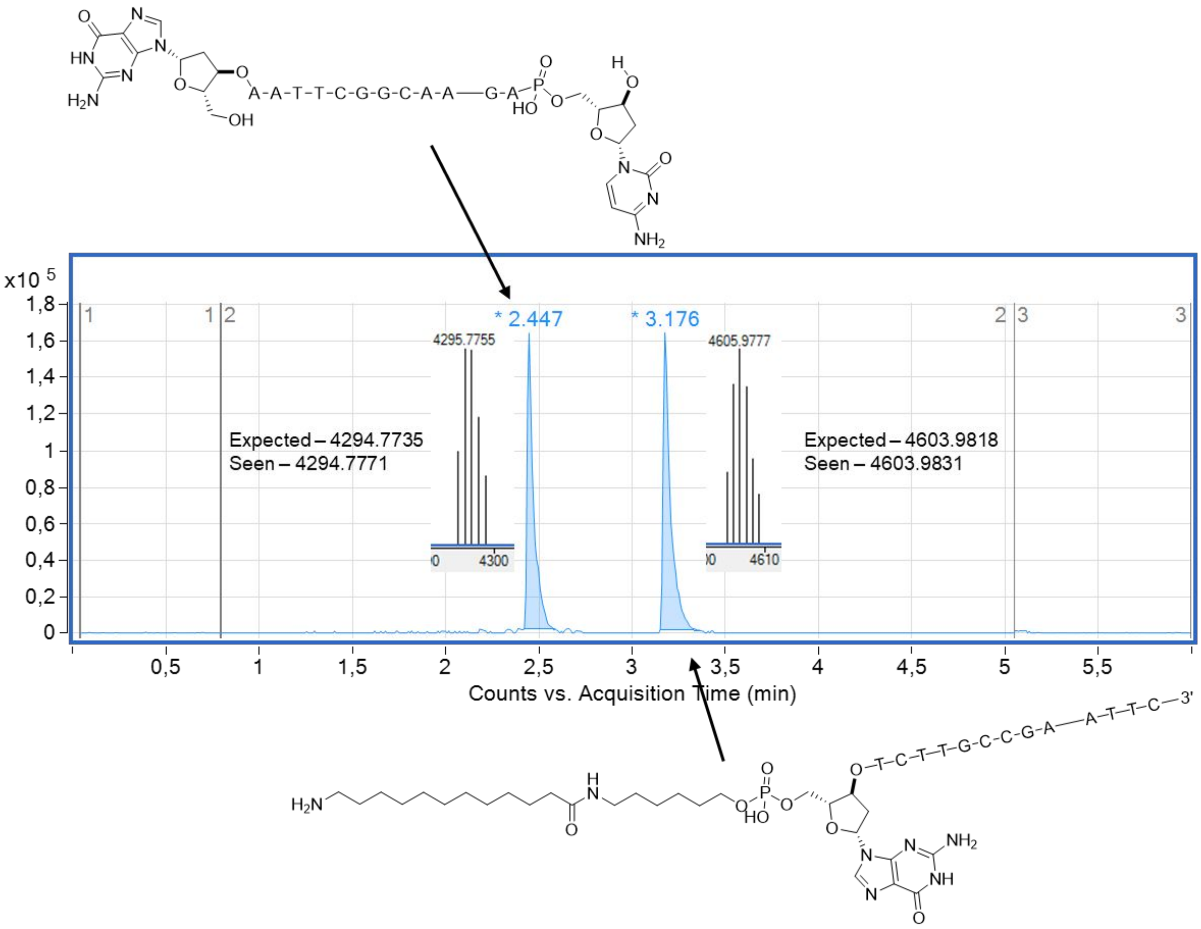

Figure S2 Chromatogram displaying double-stranded DNA starting material, **HP2** – the headpiece for Buchwald-Hartwig Aminations.

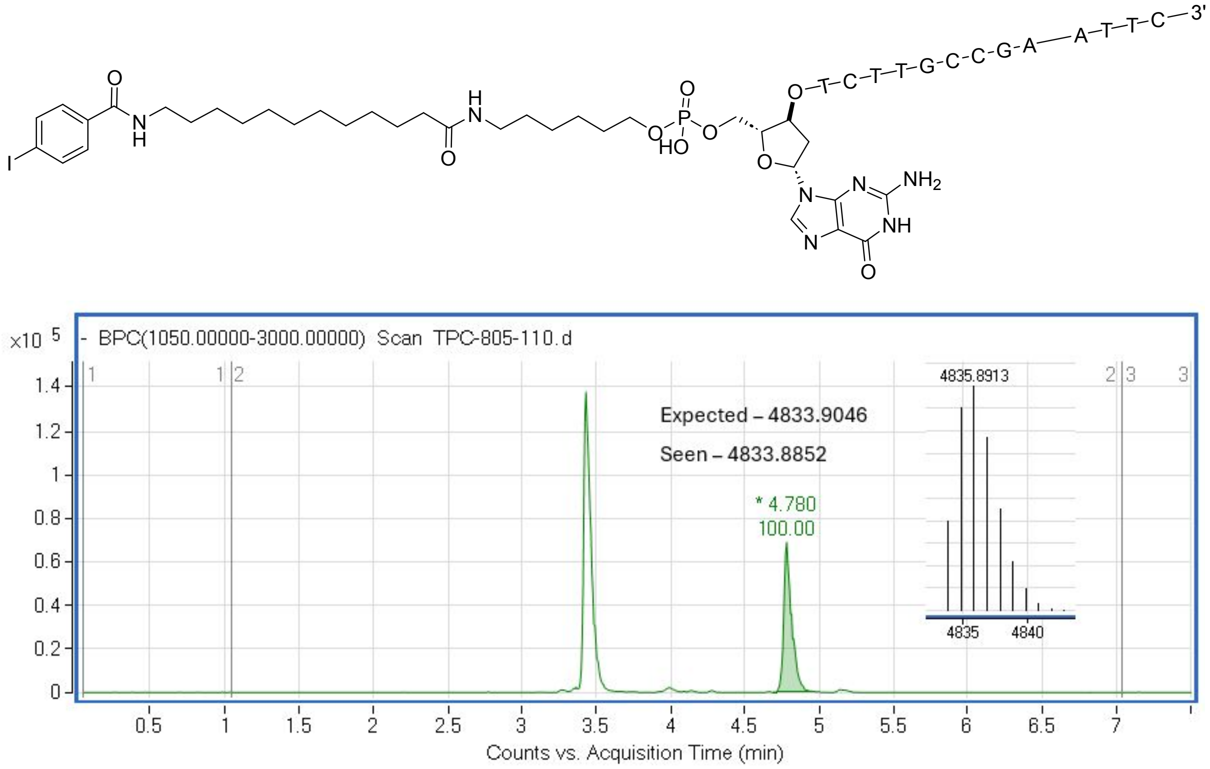

General on-DNA Aliphatic Buchwald-Hartwig Amination Procedure

Span 60 (20 μL, 5% v/v in THF) was added to 0.5 mL Eppendorf™ Polypropylene DNA LoBind Tube and concentrated using a Genevac™ Concentrator EZ-2 Plus at 50 °C for 2 h.[a] Upon complete removal of THF, DEPC-treated water (20 μL). Double-stranded DNA (4 μL, 1 nmol), amine (9 μmol), and *t*-BuOK (6 μL, 5 M in H_2O) was added to the Eppendorf and vortexed for 30s. [(Crotyl)PdCl]$_2$ (3 μL, 81 mM in THF) and *t*-BuXPhos (3 μL, 162 mM in THF) were added and the tube vortexed for 30 s. The sample was heated using a STARLAB® Thermomixer-Mixer HC at 1200 rpm at 70 °C for 1 h. After this time the reaction was allowed to cool, then sodium diethyldithiocarbamic acid (6 μL, 1 M in H_2O) was added, and the reaction was heated at 60 ˚C for a further 30 mins. The reaction mixture was allowed to cool, diluted with DEPC-treated water (2 x 100 μL) and filtered through a hydrophilic PTFE syringe filter. The reaction was then precipitated according to the general ethanol precipitation procedure and analysed by mass spectrometry.

[a]Addition of Span 60 was carried out using THF stock solution (5% v/v) due to the insolubility of Span 60 in water. Alternative surfactants added directly using aqueous stock solution (20 μL, 5% v/v).

Buchwald-Hartwig Amination (Table 6) Chromatograms

Figure S3 Chromatogram displaying double-stranded DNA product of Buchwald-Hartwig coupling between **HP2** and 2-(pyridin-3-yl)ethan-1-amine.

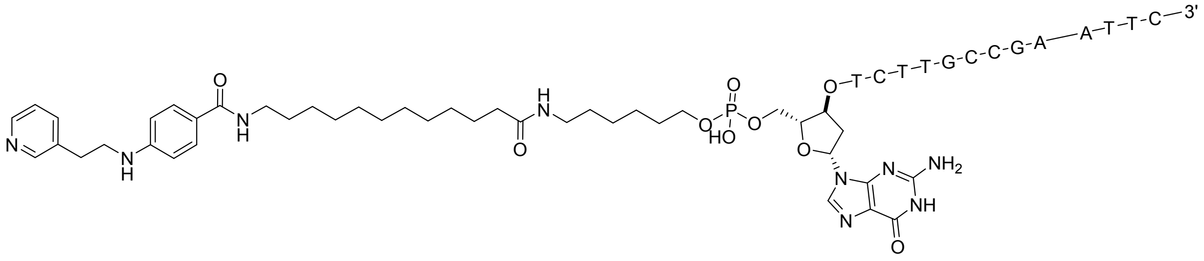

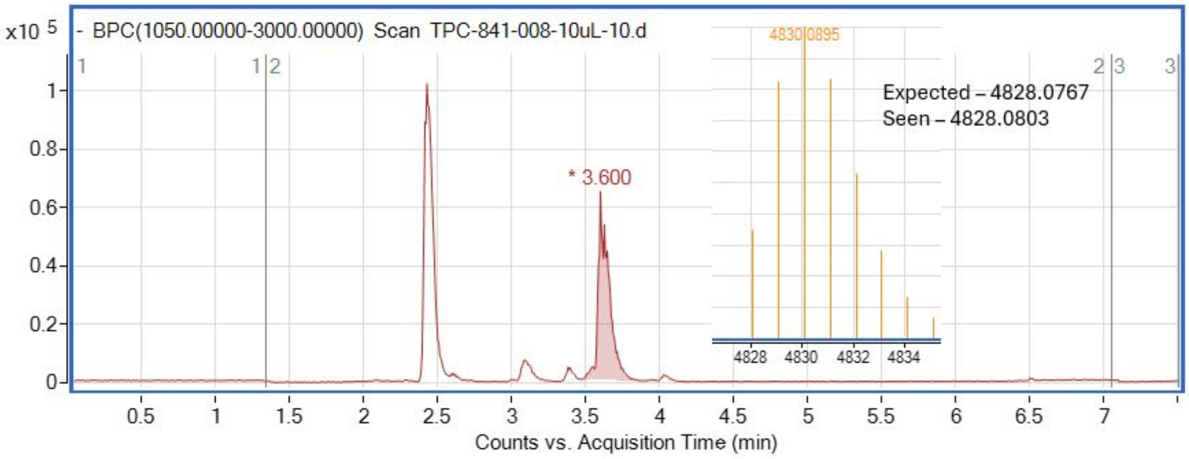

Figure S4 Chromatogram displaying double-stranded DNA product of Buchwald-Hartwig coupling between **HP2** and (2-methoxypyridin-4-yl)methanamine.

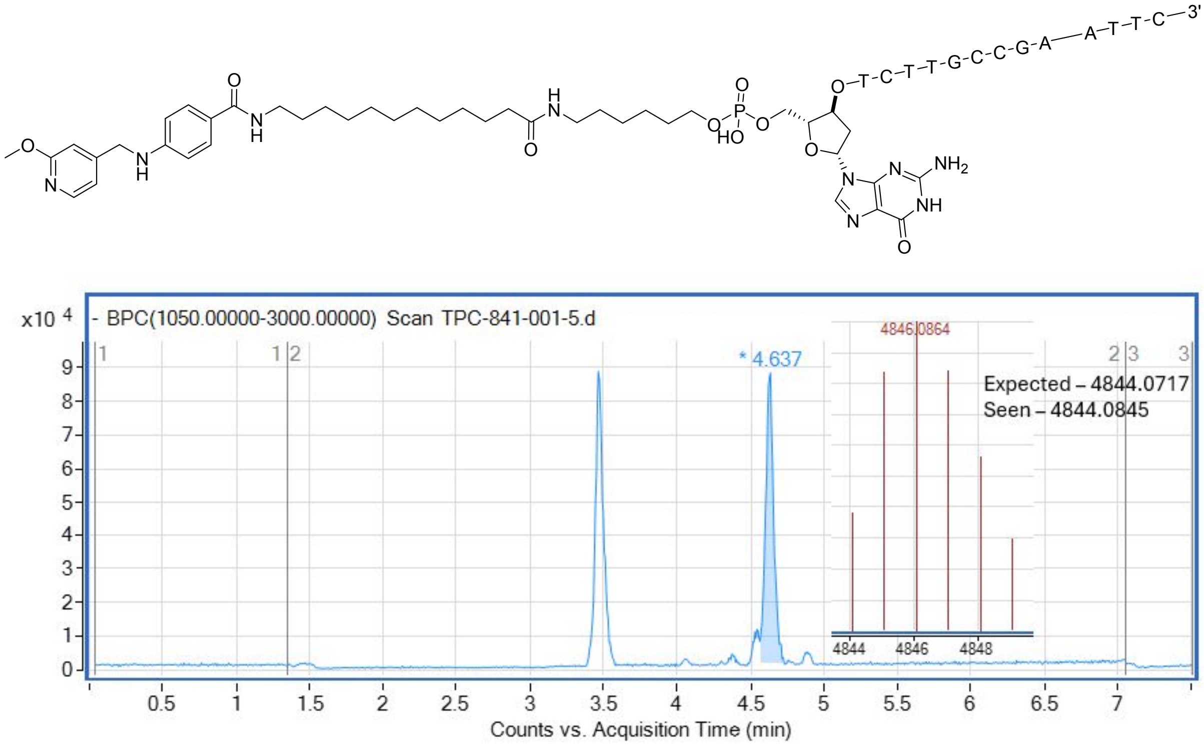

Figure S5 Chromatogram displaying double-stranded DNA product of Buchwald-Hartwig coupling between **HP2** and (*S*)-1-phenylethan-1-amine.

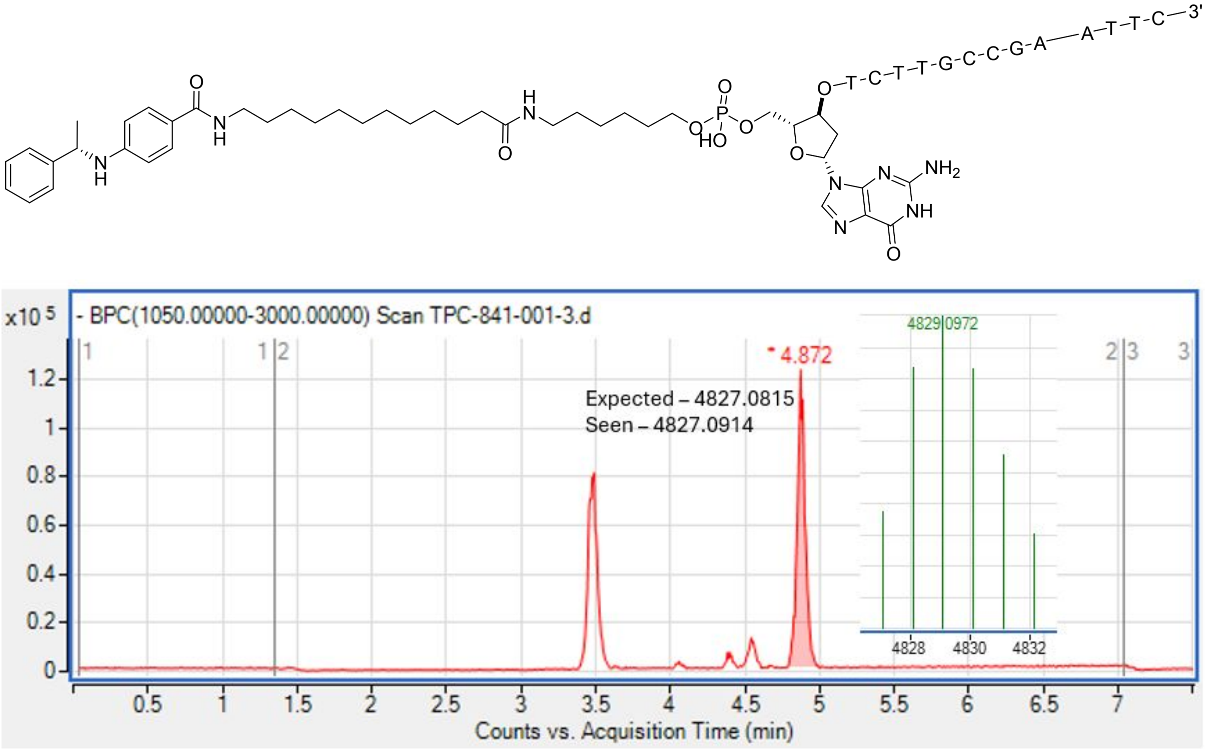

Figure S6 Chromatogram displaying double-stranded DNA product of Buchwald-Hartwig coupling between **HP2** and thiophen-2-ylmethanamine.

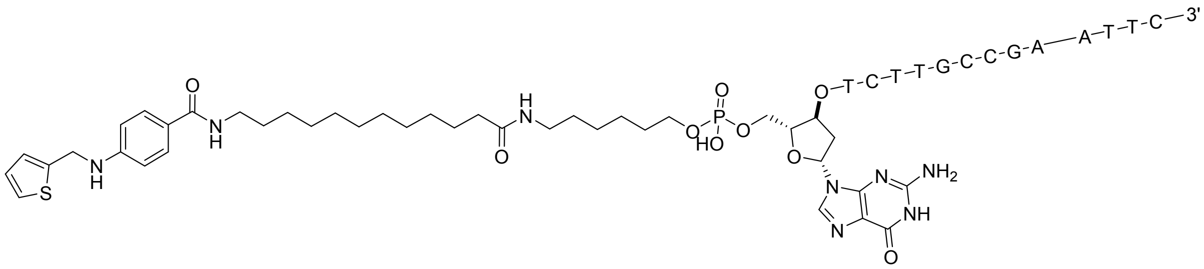

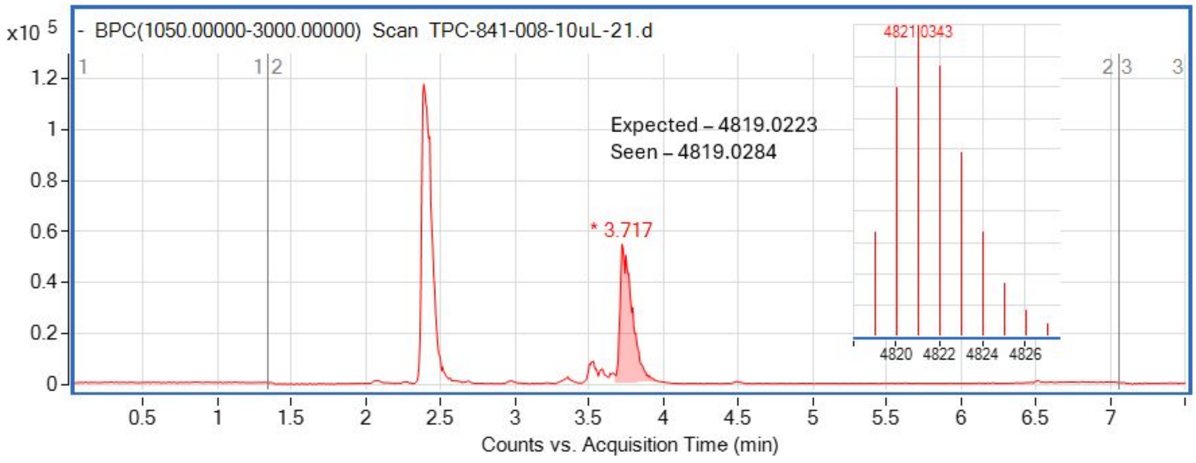

Figure S7 Chromatogram displaying double-stranded DNA product of Buchwald-Hartwig coupling between **HP2** and pyridin-4-ylmethanamine.

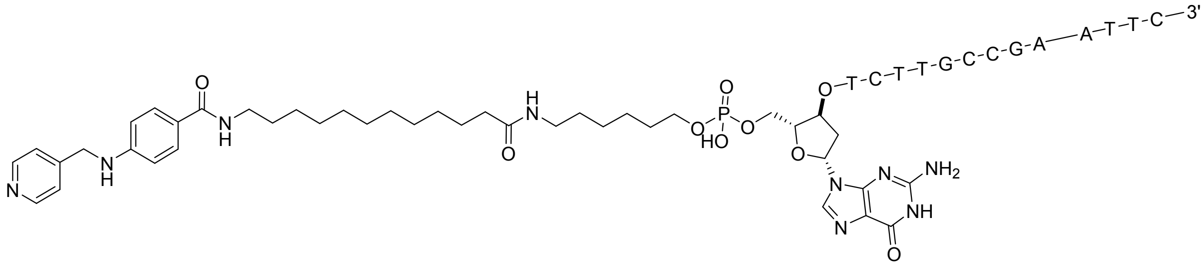

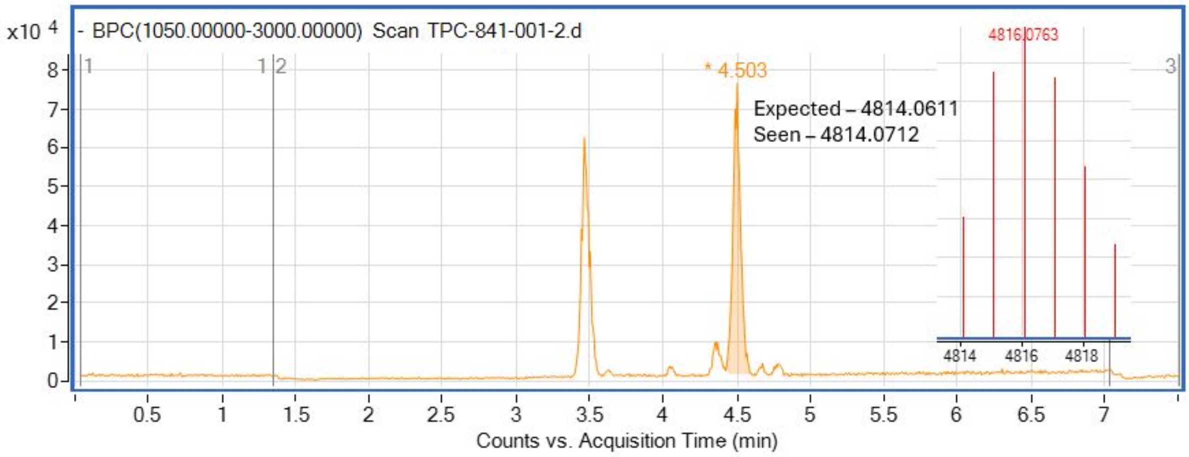

Figure S8 Chromatogram displaying double-stranded DNA product of Buchwald-Hartwig coupling between **HP2** and (5-methylfuran-2-yl)methanamine pyridin-3-ylmethanamine.

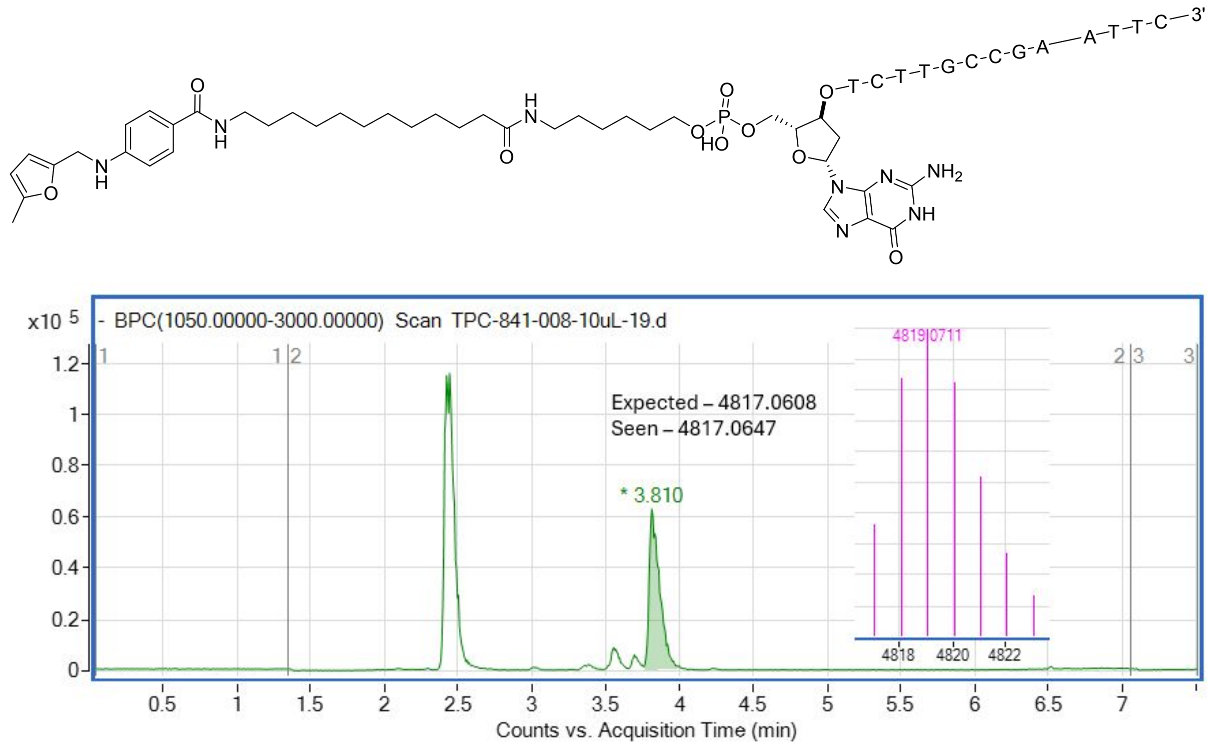

Figure S9 Chromatogram displaying double-stranded DNA product of Buchwald-Hartwig coupling between **HP2** and pyridin-3-ylmethanamine.

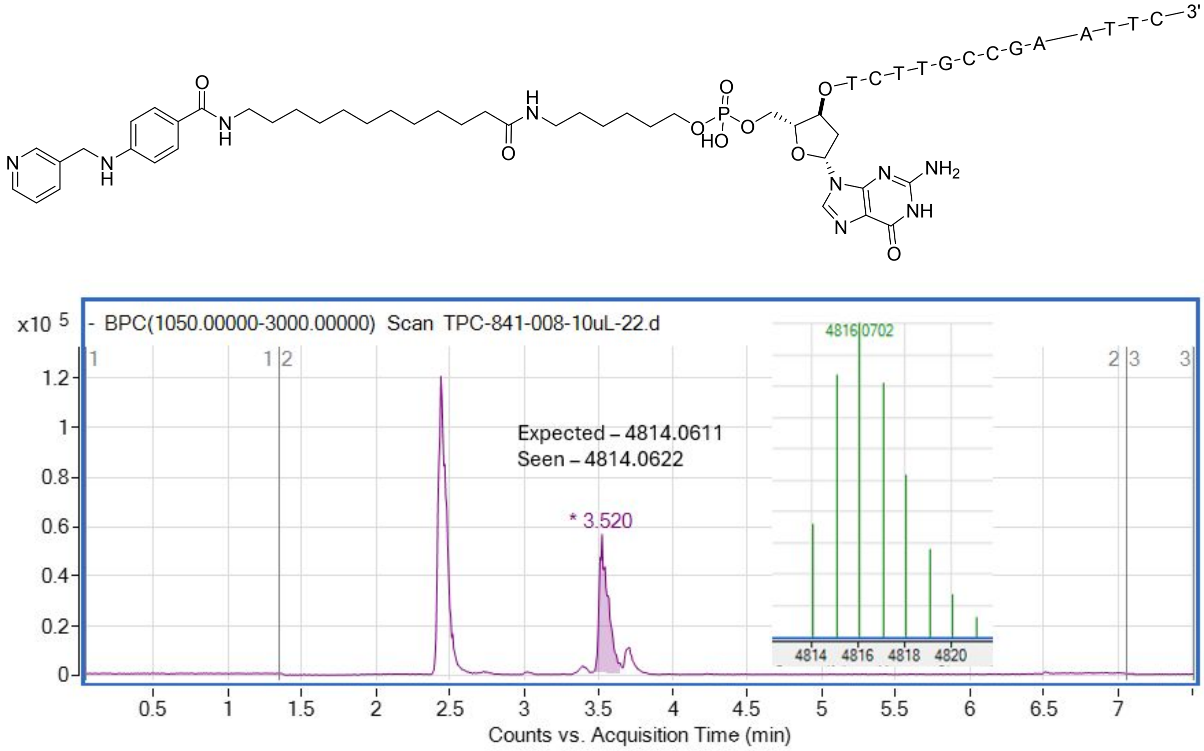

Figure S10 Chromatogram displaying double-stranded DNA product of Buchwald-Hartwig coupling between **HP2** and (3-methoxyphenyl)methanamine.

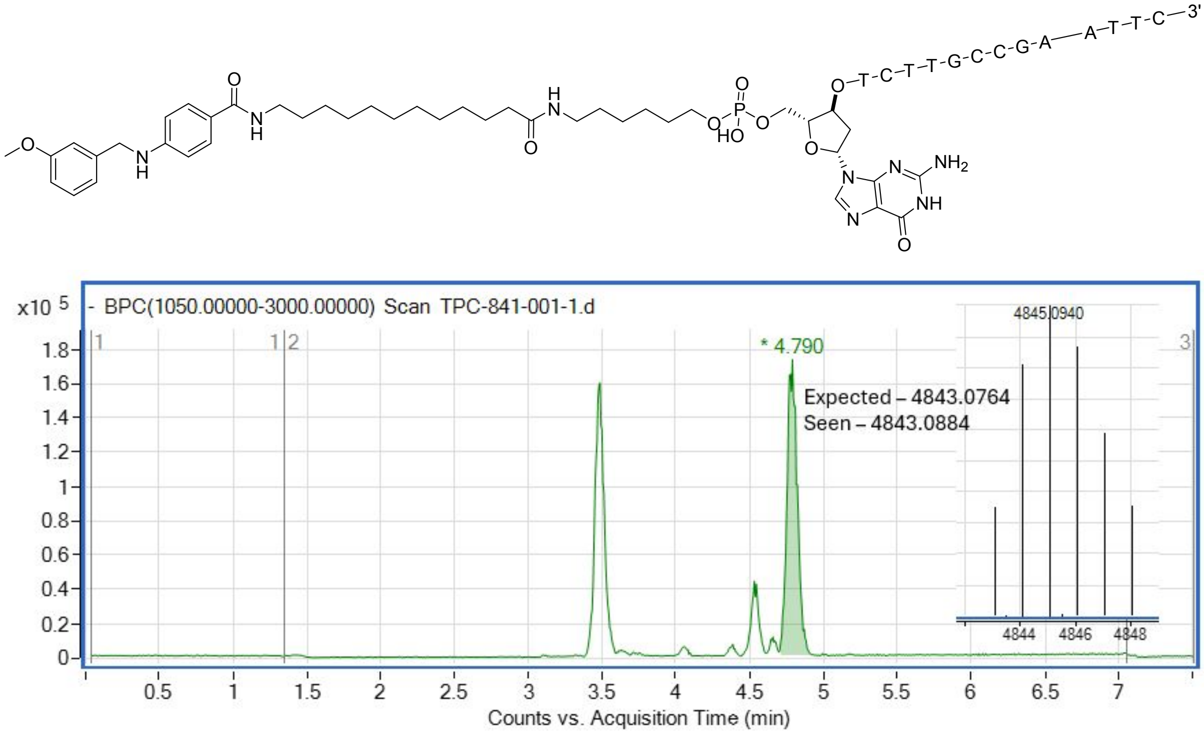

Figure S11 Chromatogram displaying double-stranded DNA product of Buchwald-Hartwig coupling between **HP2** and phenylmethanamine.

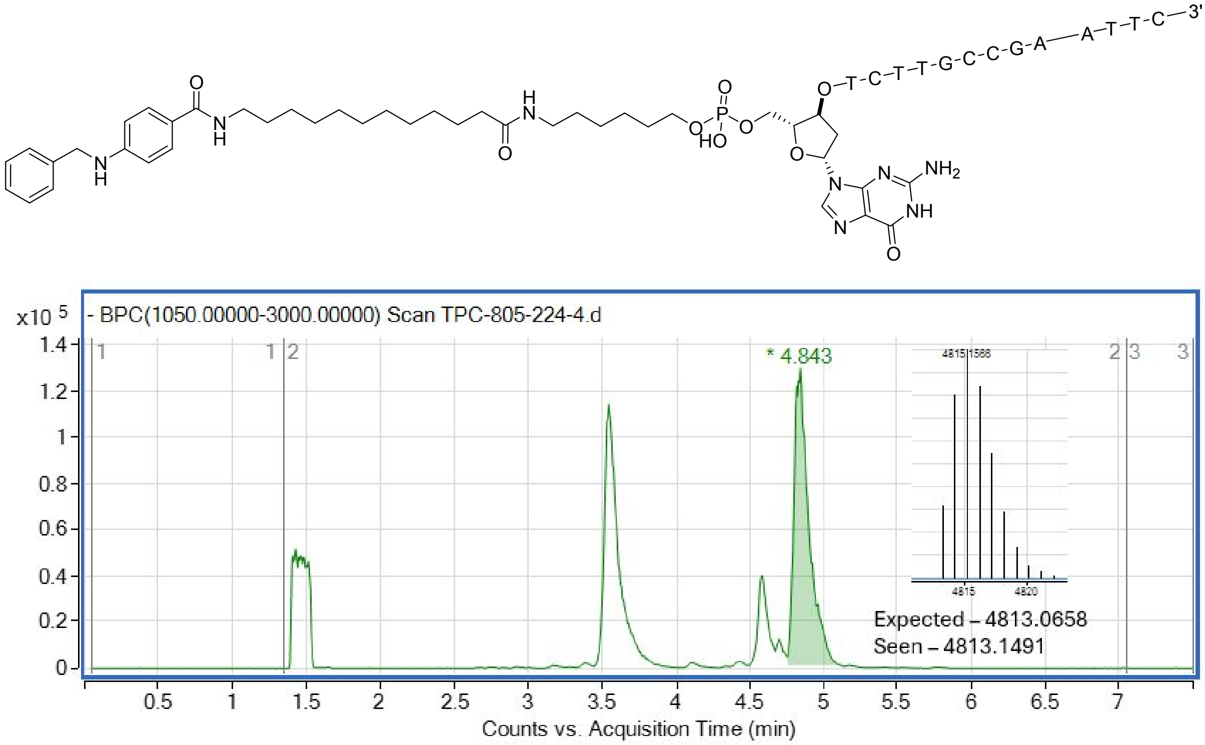

Figure S12 Chromatogram displaying double-stranded DNA product of Buchwald-Hartwig coupling between **HP2** and *N*-methyl-1-phenylmethanamine.

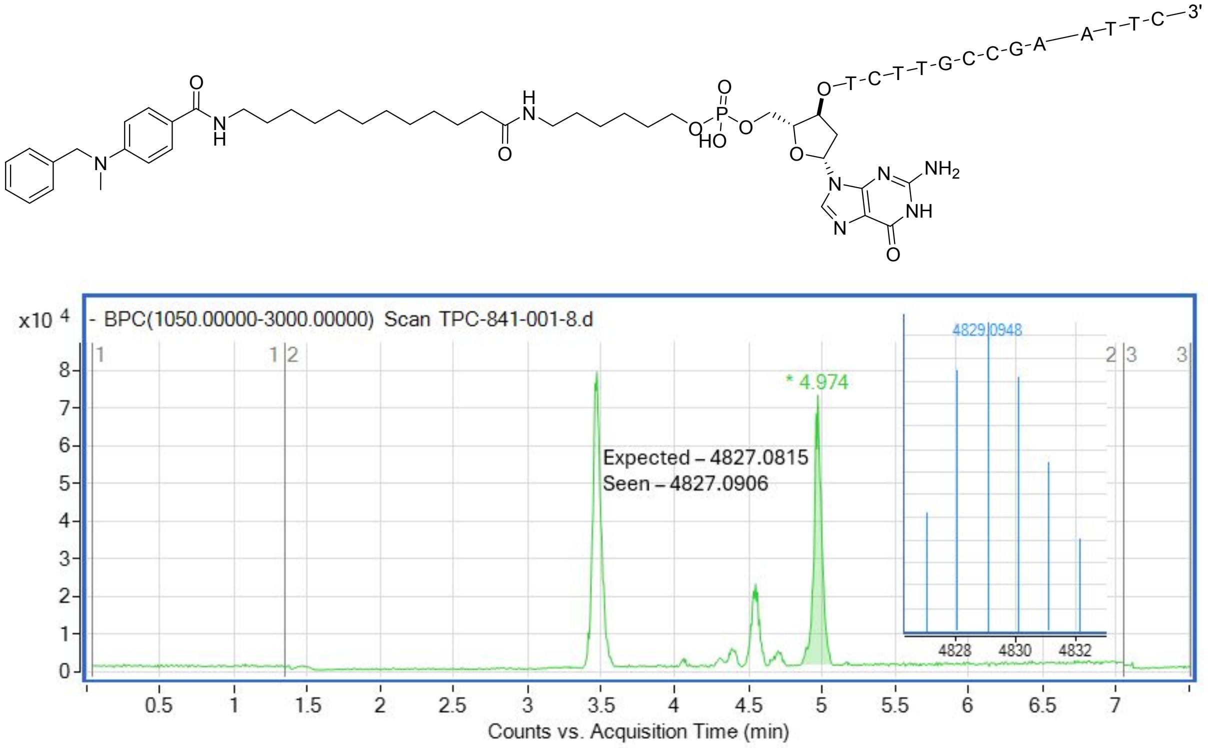

Figure S13 Chromatogram displaying double-stranded DNA product of Buchwald-Hartwig coupling between **HP2** and *p*-tolylmethanamine.

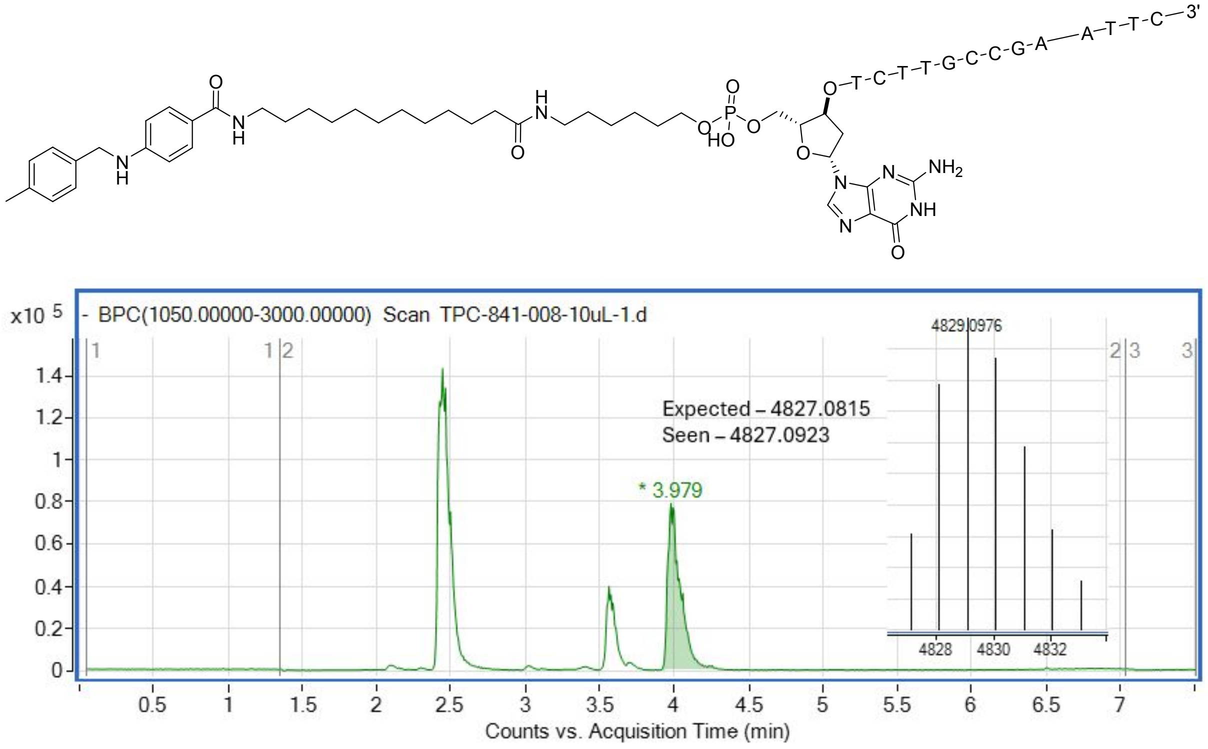

Figure S14 Chromatogram displaying double-stranded DNA product of Buchwald-Hartwig coupling between **HP2** and pyridin-2-ylmethanamine.

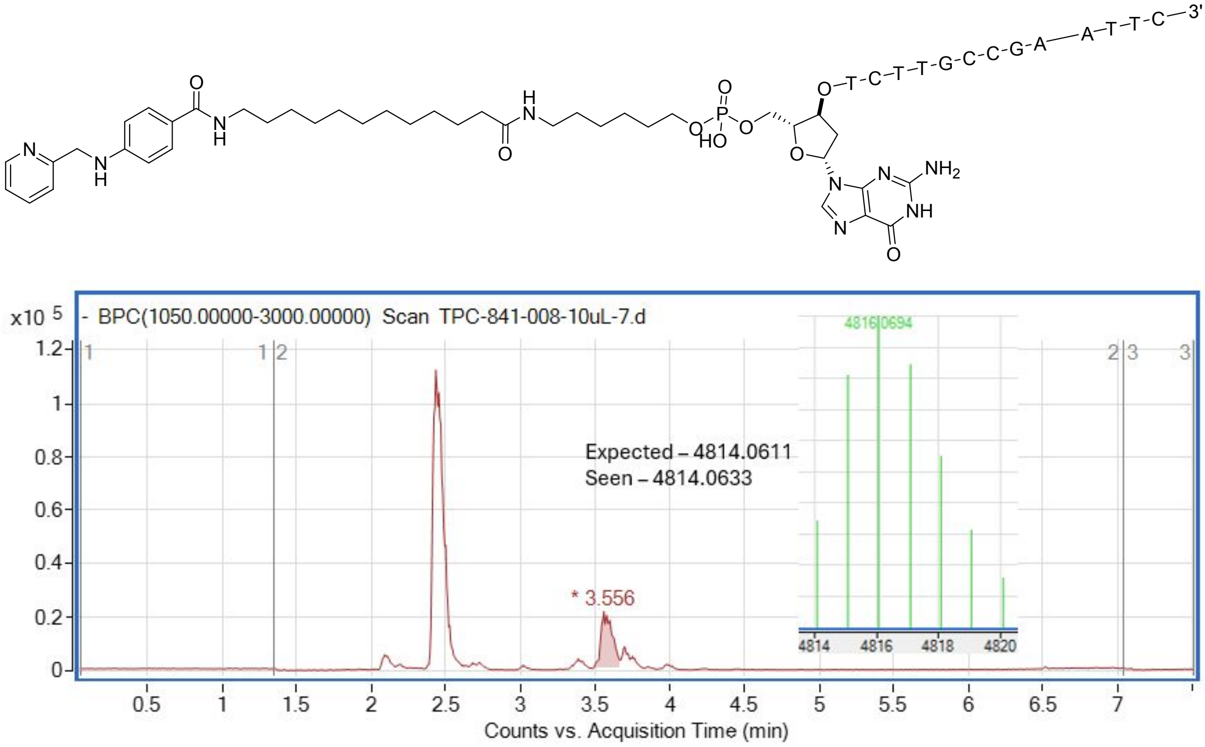

Figure S15 Chromatogram displaying double-stranded DNA product of Buchwald-Hartwig coupling between **HP2** and 2-phenylethan-1-amine.

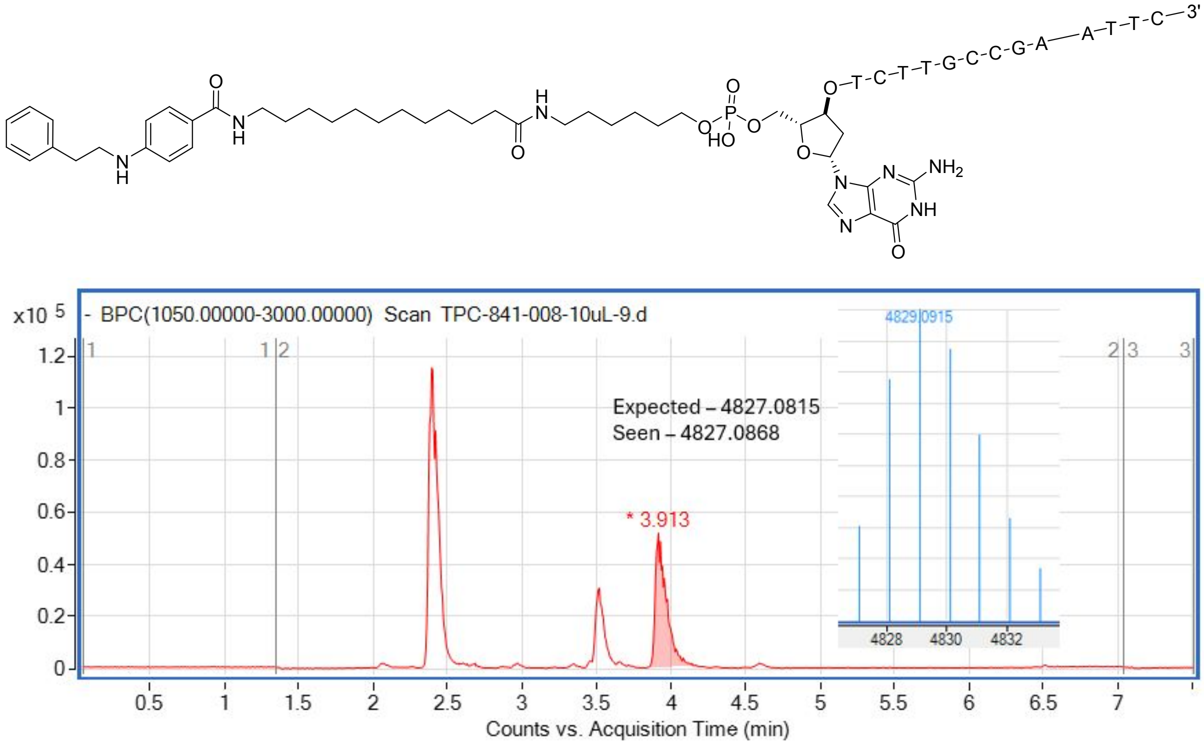

Figure S16 Chromatogram displaying double-stranded DNA product of Buchwald-Hartwig coupling between **HP2** and (4-fluorophenyl)methanamine.

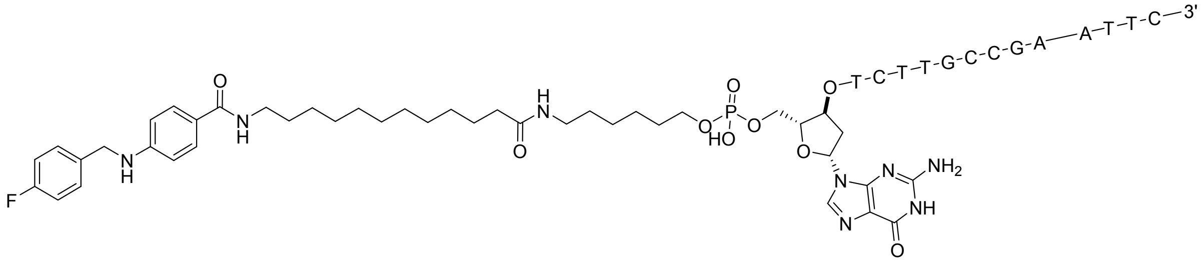

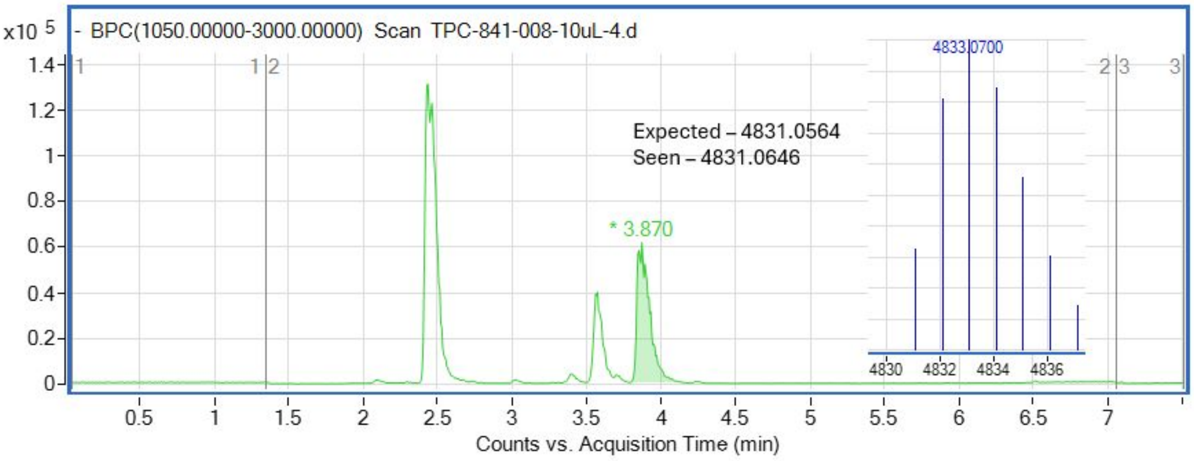

Figure S17 Chromatogram displaying double-stranded DNA product of Buchwald-Hartwig coupling between **HP2** and (3,4-dimethoxyphenyl)methanamine.

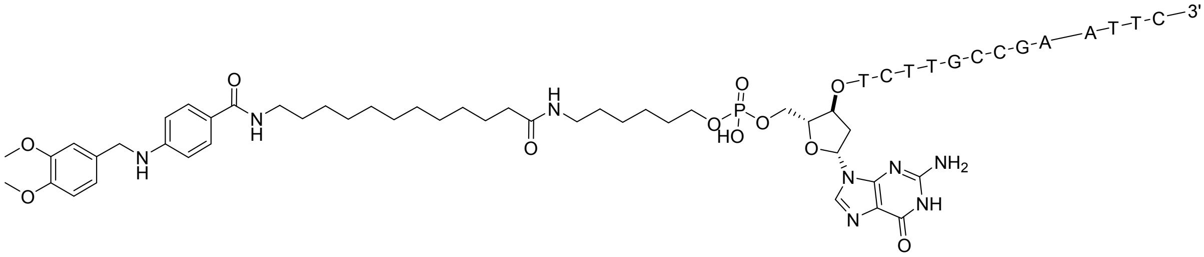

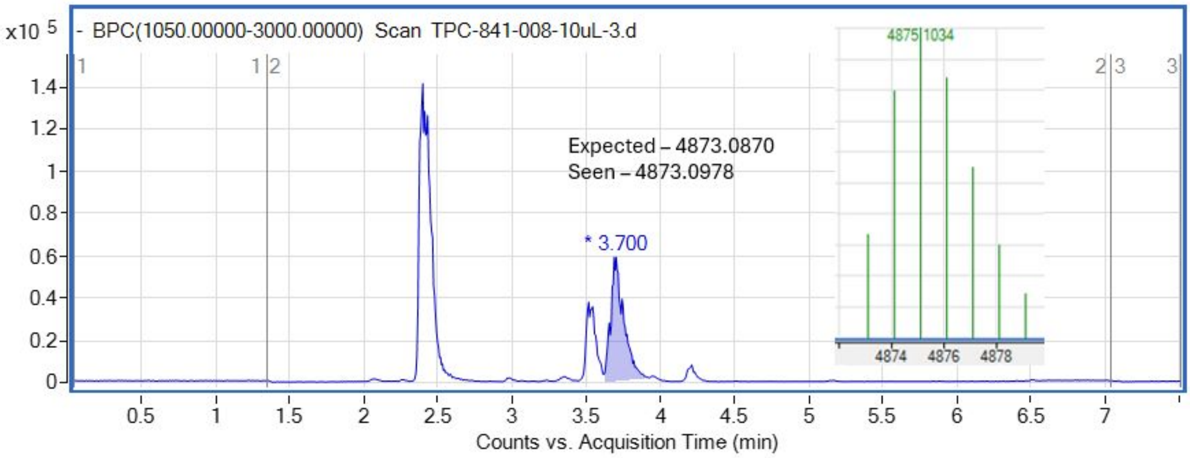

Figure S18 Chromatogram displaying double-stranded DNA product of Buchwald-Hartwig coupling between **HP2** and 1-(4-fluorophenyl)ethan-1-amine.

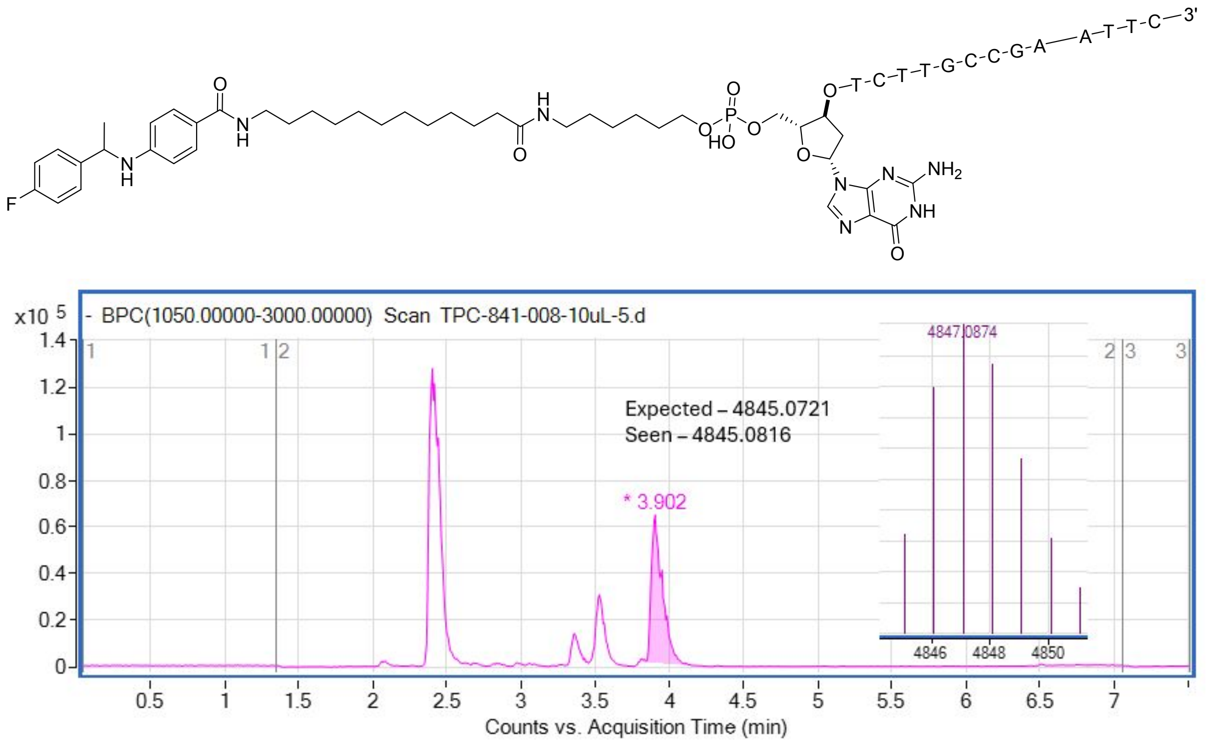

Figure S19 Chromatogram displaying double-stranded DNA product of Buchwald-Hartwig coupling between **HP2** and (2-fluorophenyl)methanamine.

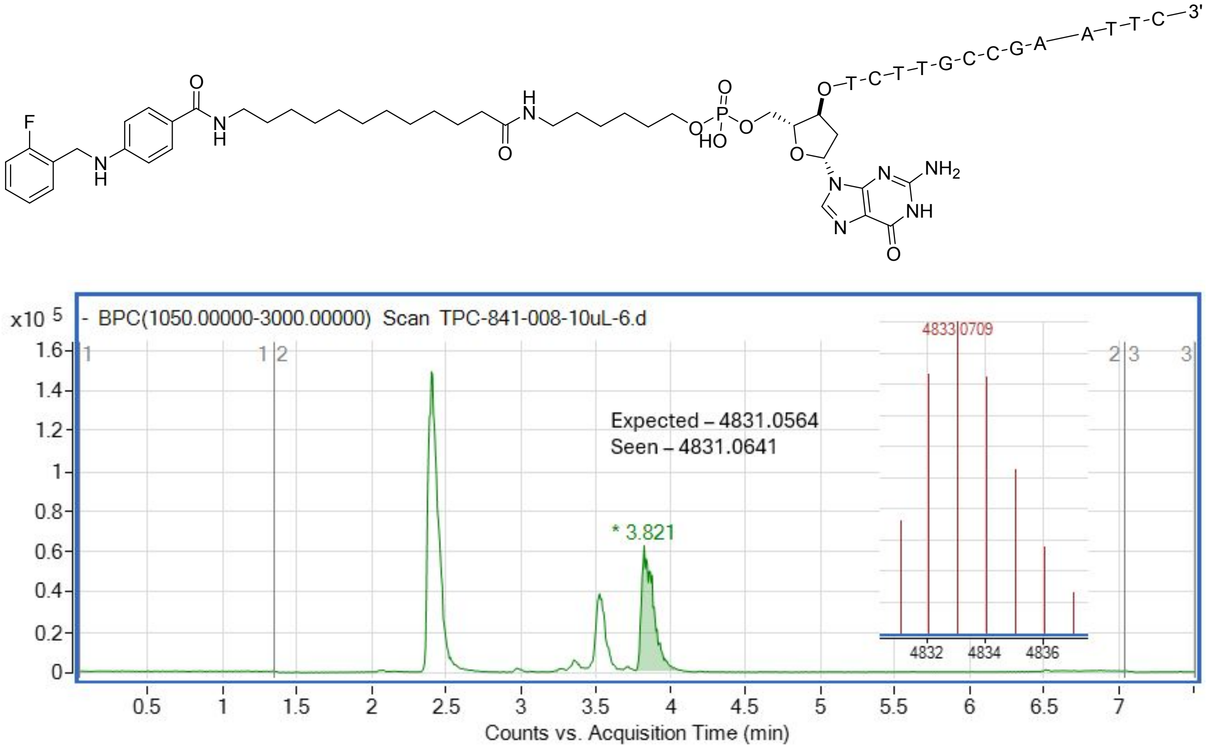

Figure S20 Chromatogram displaying double-stranded DNA product of Buchwald-Hartwig coupling between **HP2** and (4-methoxyphenyl)methanamine.

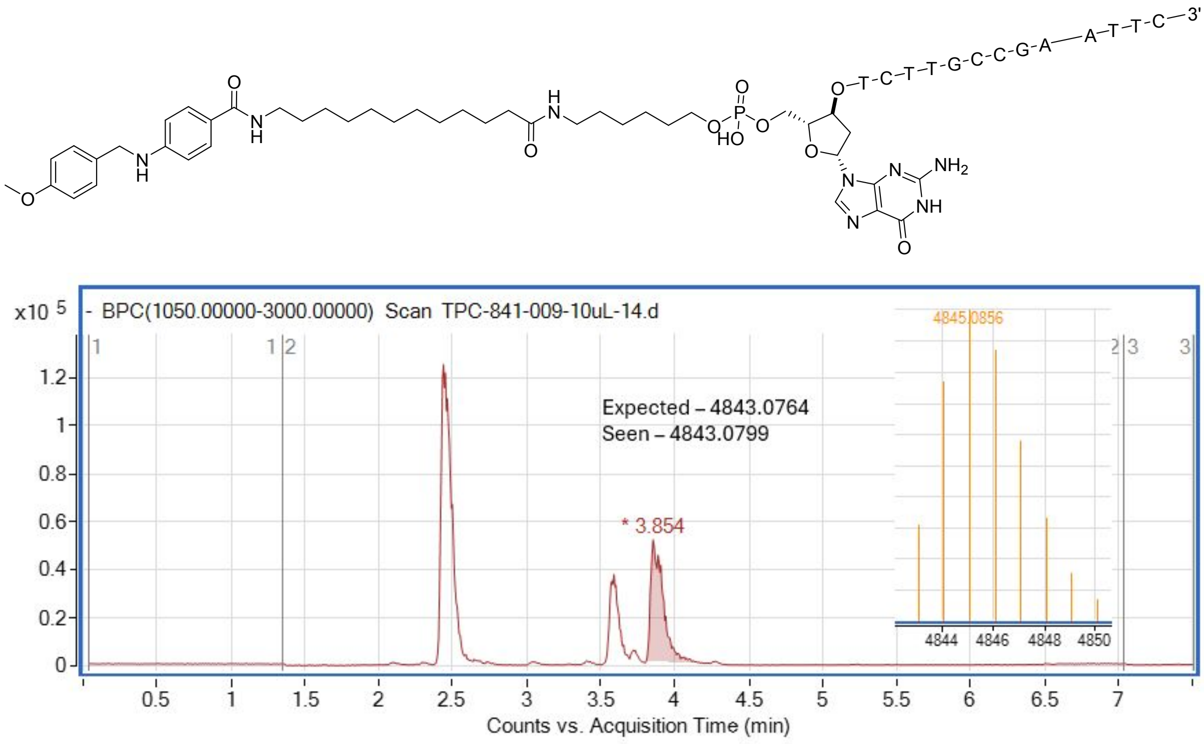

Figure S21 Chromatogram displaying double-stranded DNA product of Buchwald-Hartwig coupling between **HP2** and (2-methoxyphenyl)methanamine.

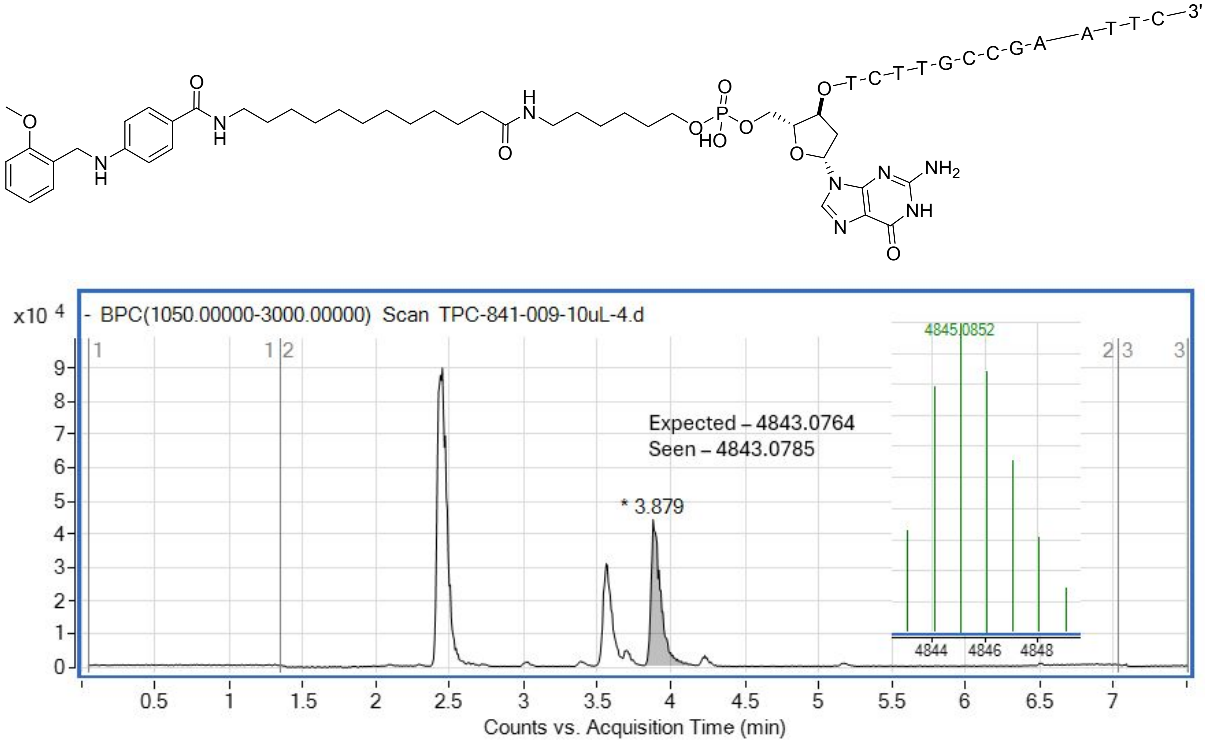

Figure S22 Chromatogram displaying double-stranded DNA product of Buchwald-Hartwig coupling between **HP2** and 1-(4-methoxyphenyl)-*N*-methylmethanamine.

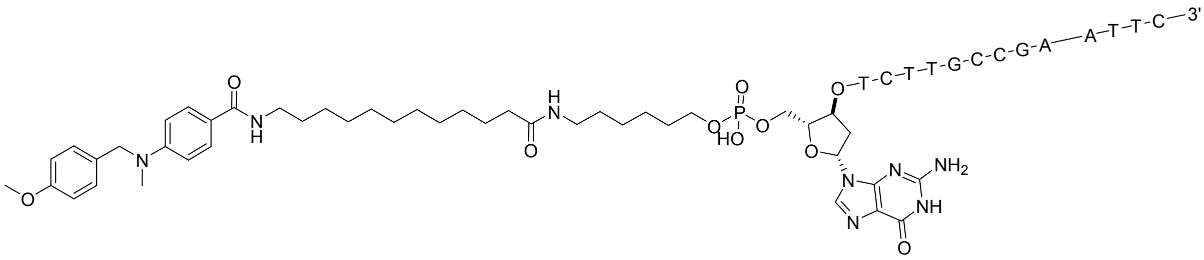

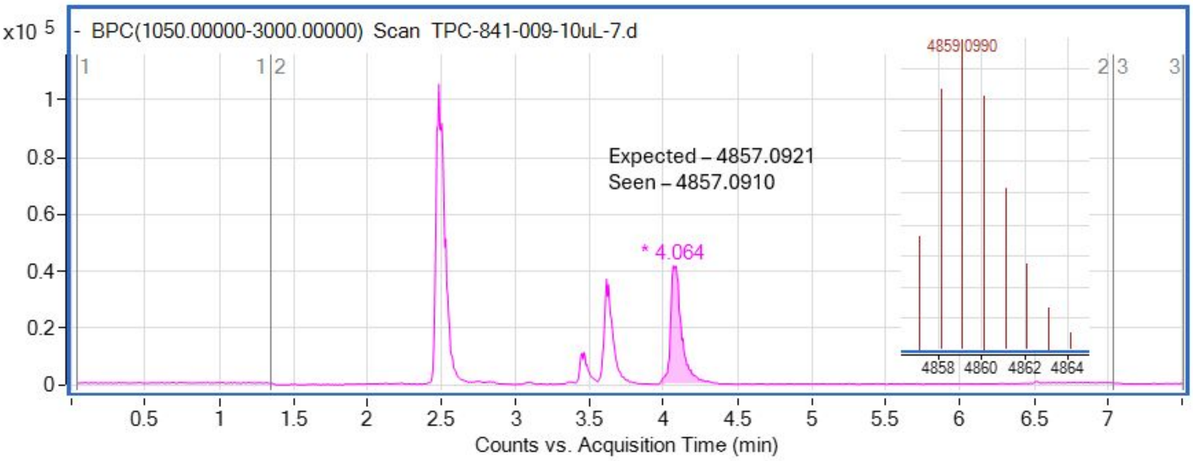

Figure S23 Chromatogram displaying double-stranded DNA product of Buchwald-Hartwig coupling between **HP2** and 3-(1*H*-imidazol-1-yl)propan-1-amine.

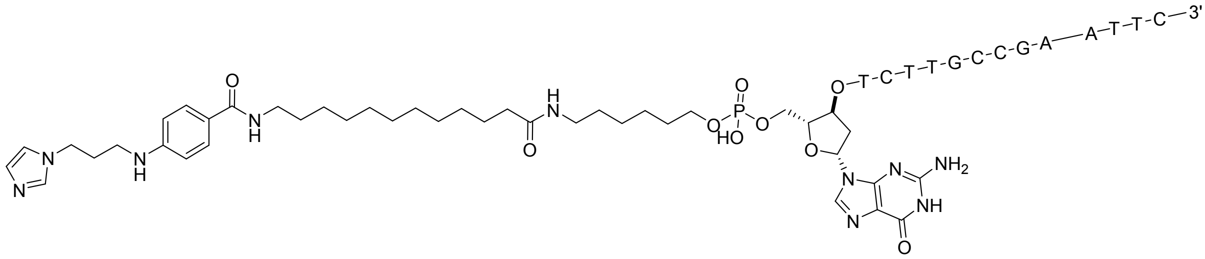

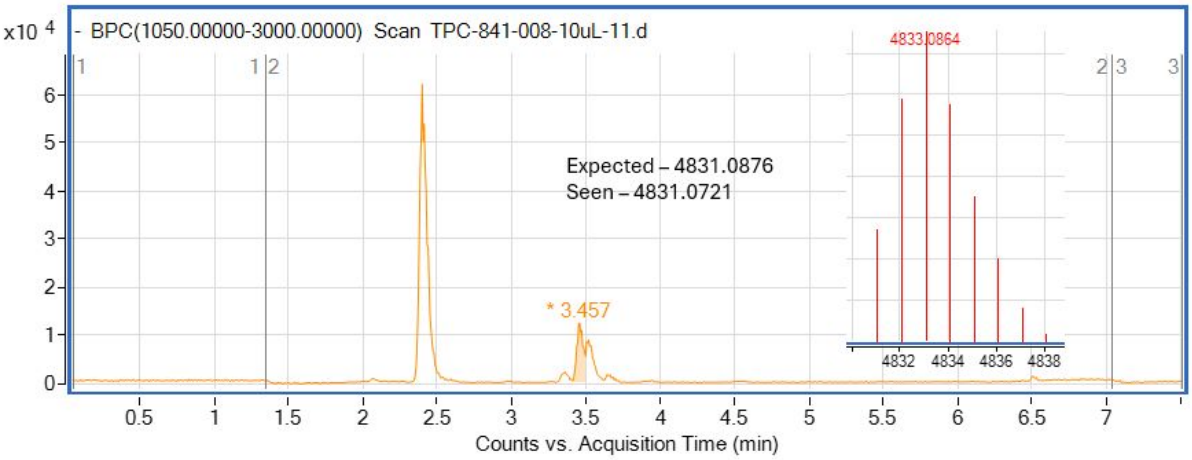

Figure S24 Chromatogram displaying double-stranded DNA product of Buchwald-Hartwig coupling between **HP2** and (*R*)-1-(4-methoxyphenyl)ethan-1-amine.

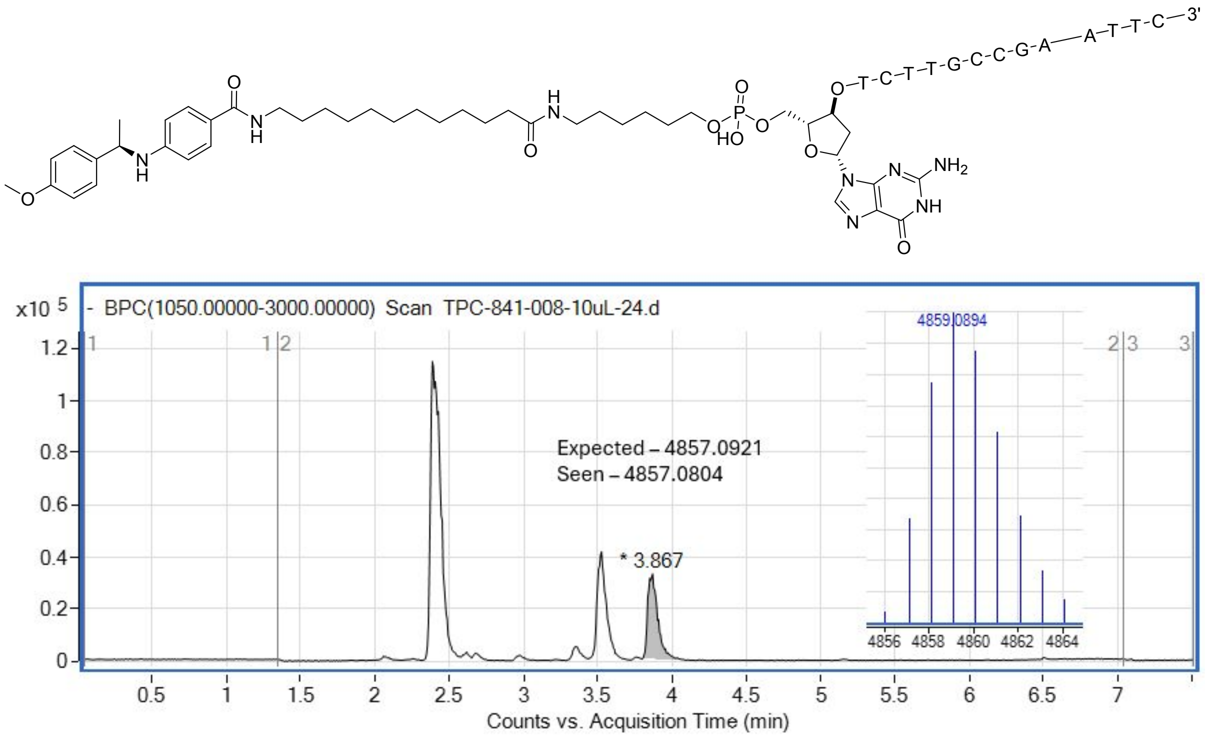

Figure S25 Chromatogram displaying double-stranded DNA product of Buchwald-Hartwig coupling between **HP2** and (2,4-dimethoxyphenyl)methanamine.

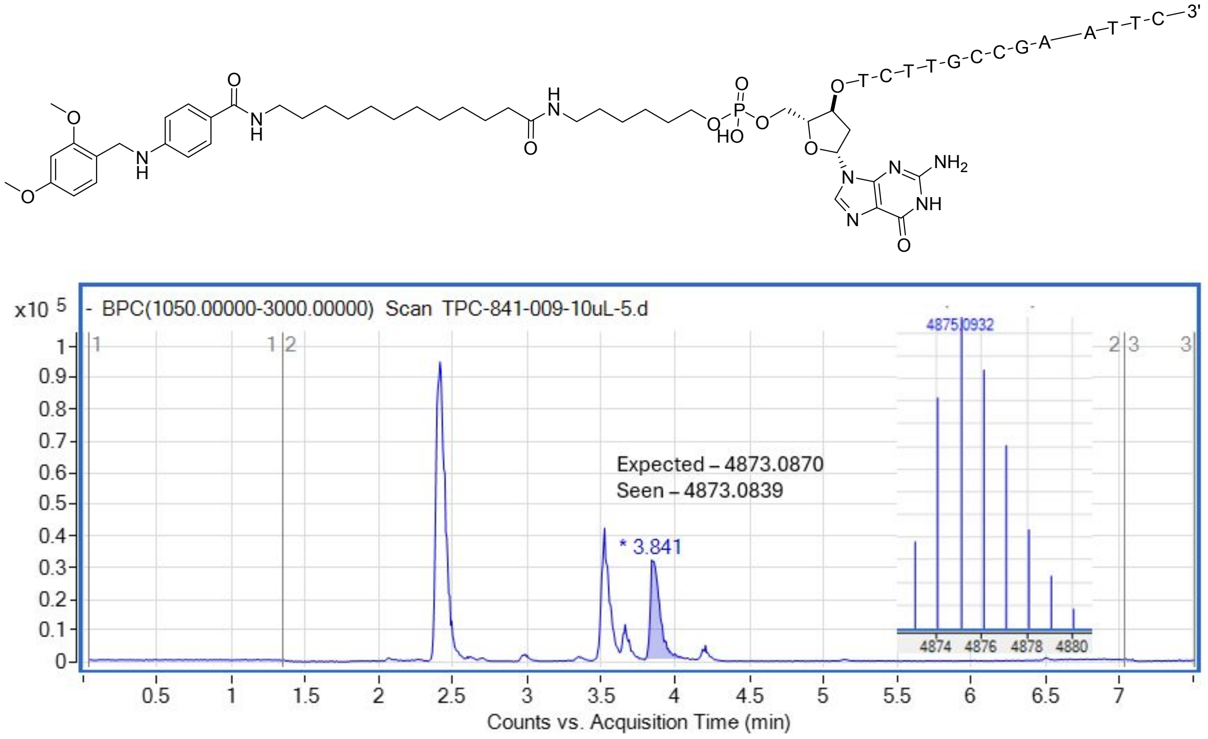

Figure S26 Chromatogram displaying double-stranded DNA product of Buchwald-Hartwig coupling between **HP2** and 4-(aminomethyl)-*N,N*-dimethylaniline.

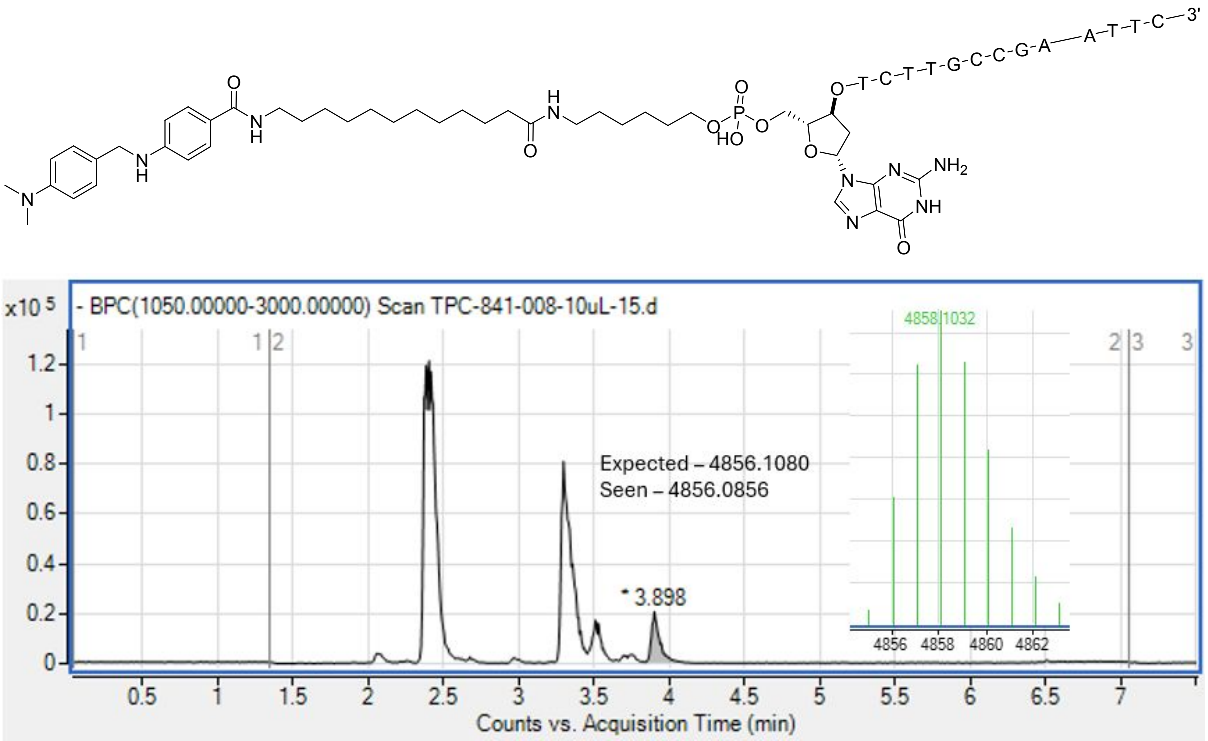

Figure S27 Chromatogram displaying double-stranded DNA product of Buchwald-Hartwig coupling between **HP2** and cyclohexylmethanamine.

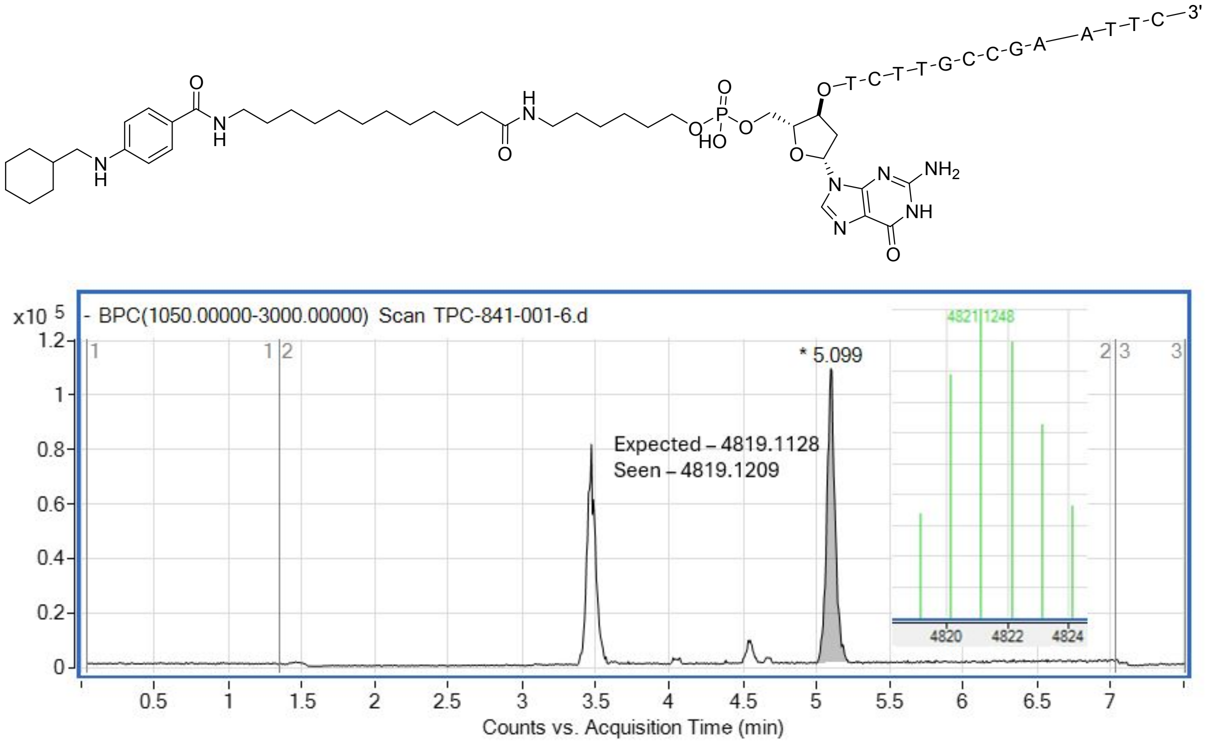

Figure S28 Chromatogram displaying double-stranded DNA product of Buchwald-Hartwig coupling between **HP2** and piperidine.

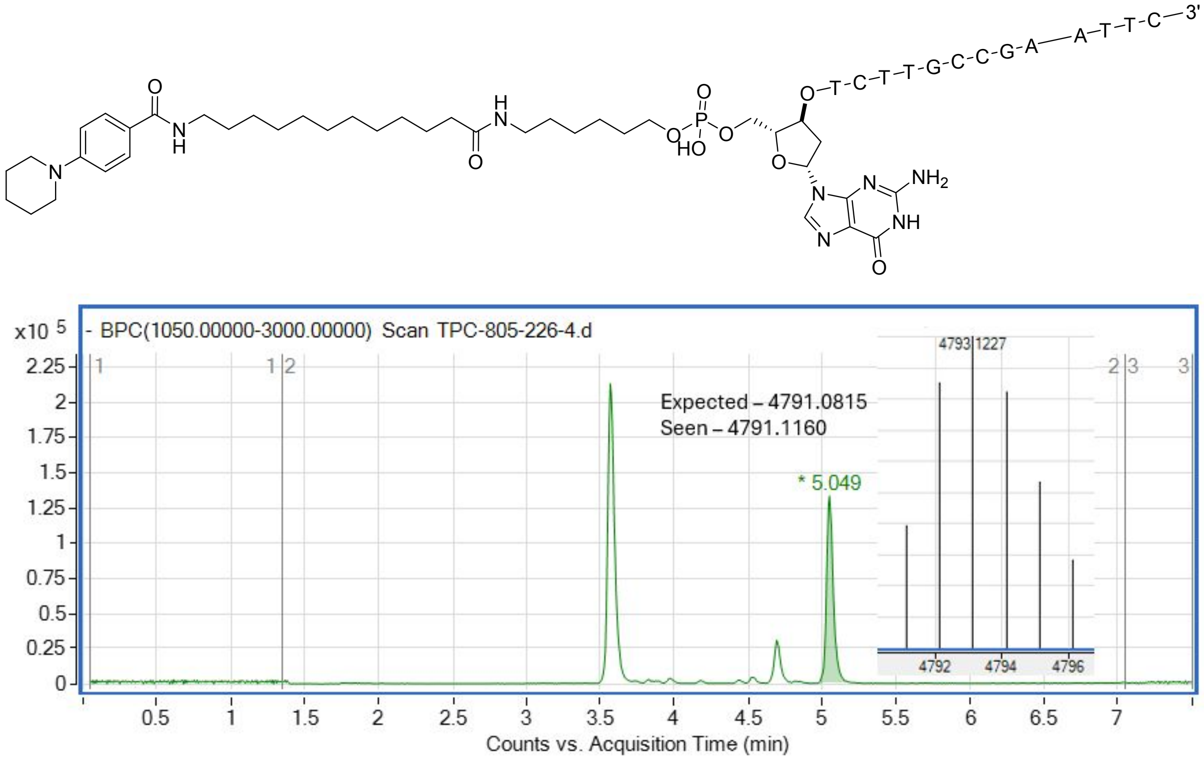

Figure S29 Chromatogram displaying double-stranded DNA product of Buchwald-Hartwig coupling between **HP2** and cyclohexanamine.

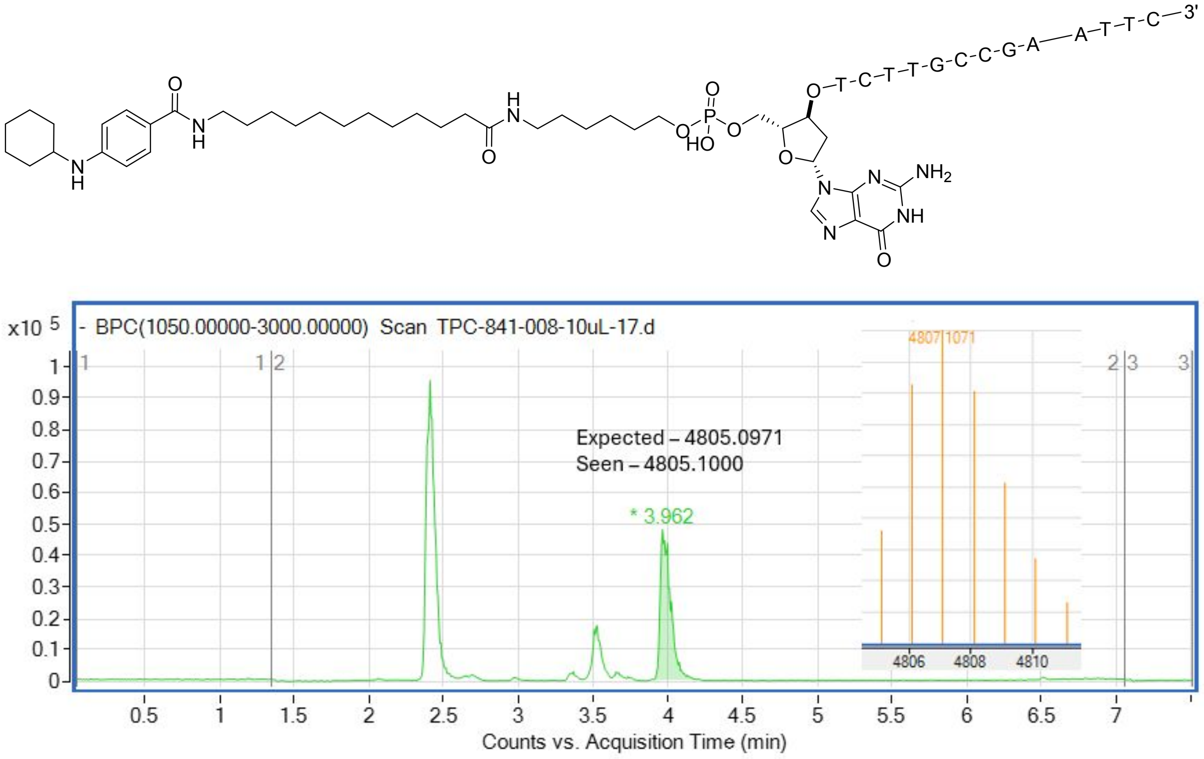

Figure S30 Chromatogram displaying double-stranded DNA product of Buchwald-Hartwig coupling between **HP2** and cyclopentanamine.

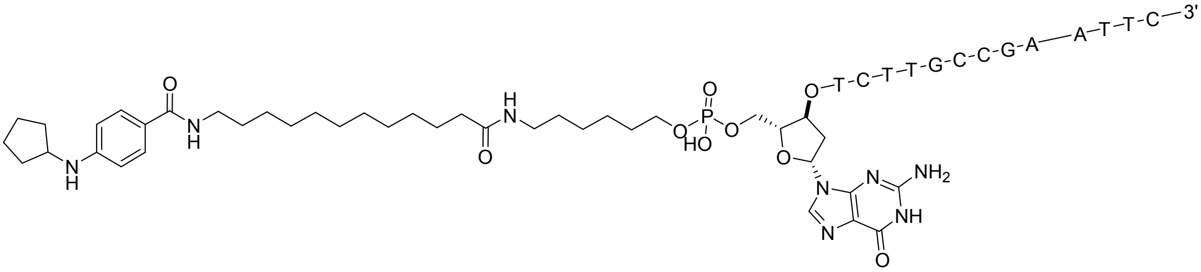

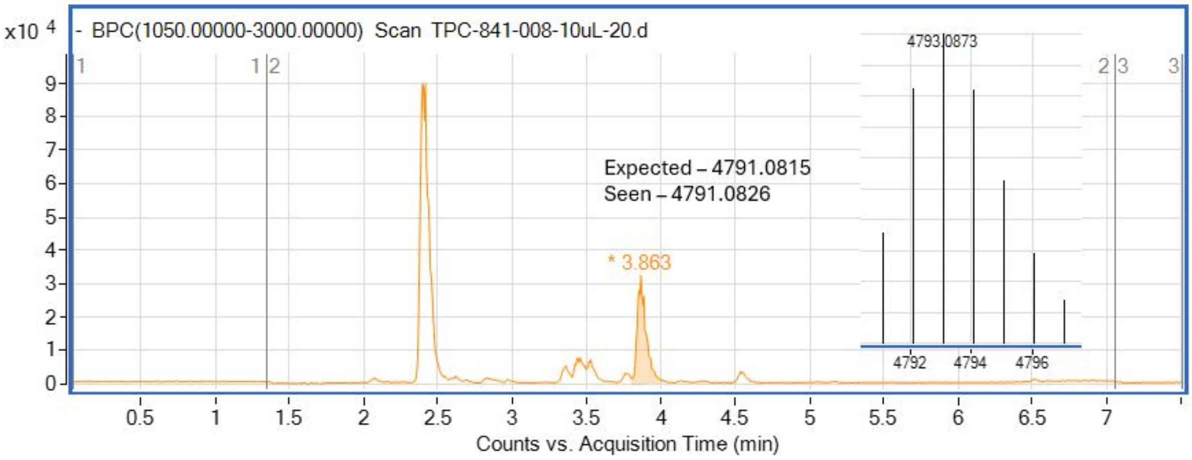

Figure S31 Chromatogram displaying double-stranded DNA product of Buchwald-Hartwig coupling between **HP2** and 3-methylbutan-1-amine.

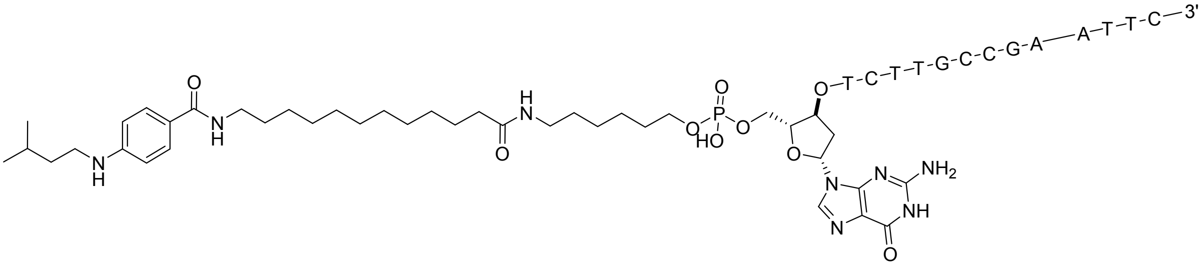

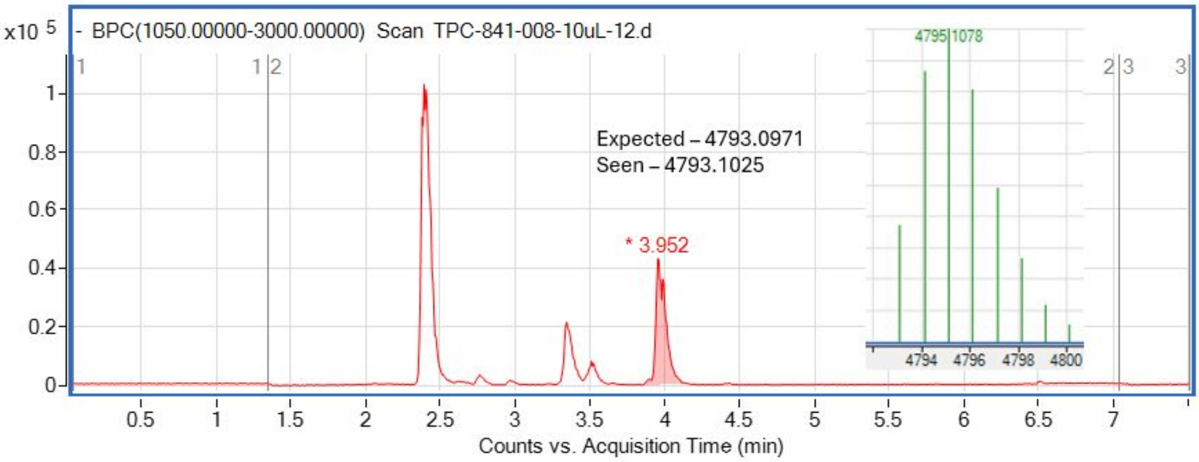

Figure S32 Chromatogram displaying double-stranded DNA product of Buchwald-Hartwig coupling between **HP2** and (*S*)-2-(trifluoromethyl)pyrrolidine.

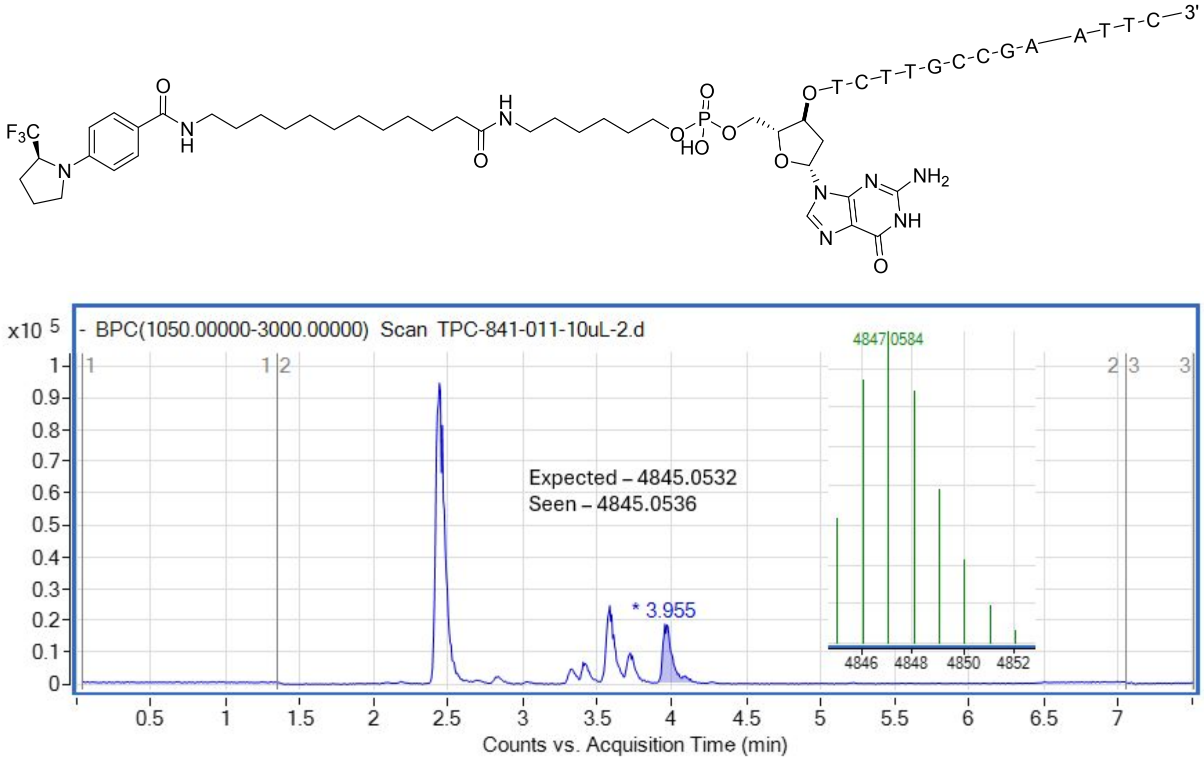

Figure S33 Chromatogram displaying double-stranded DNA product of Buchwald-Hartwig coupling between **HP2** and *N*-methylcyclohexanamine.

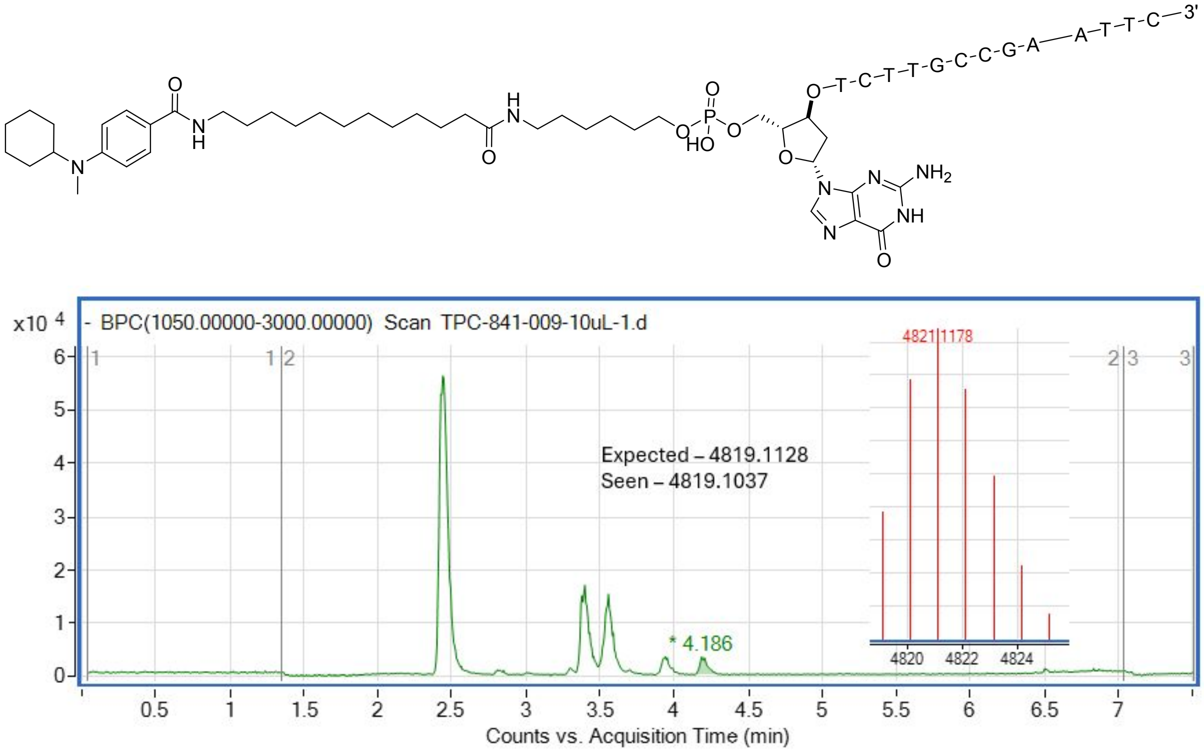

Figure S34 Chromatogram displaying double-stranded DNA product of Buchwald-Hartwig coupling between **HP2** and (2*S*,6*R*)-2,6-dimethylmorpholine.

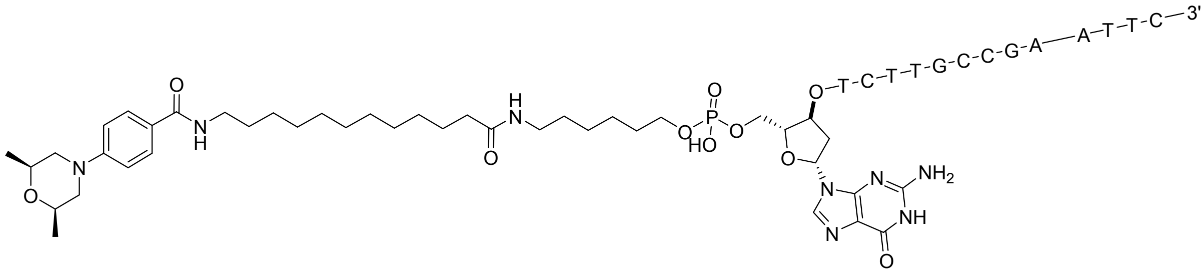

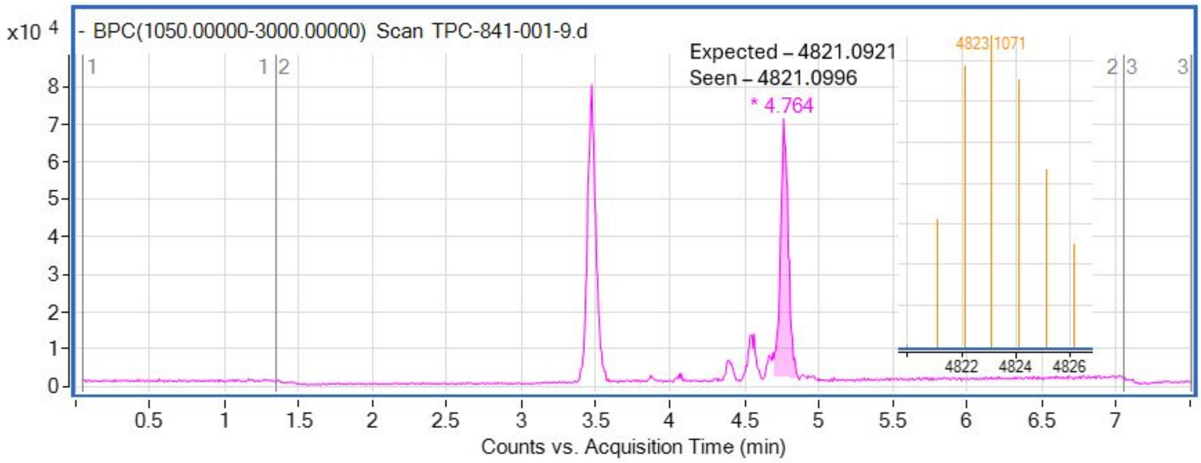

Figure S35 Chromatogram displaying double-stranded DNA product of Buchwald-Hartwig coupling between **HP2** and 2-(4-methylpiperazin-1-yl)ethan-1-amine.

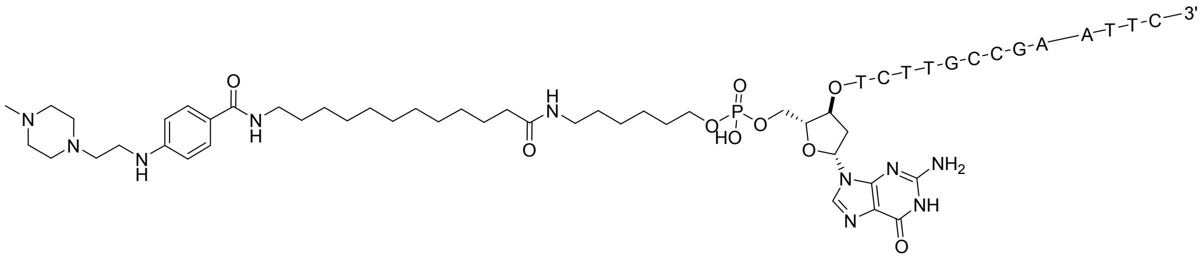

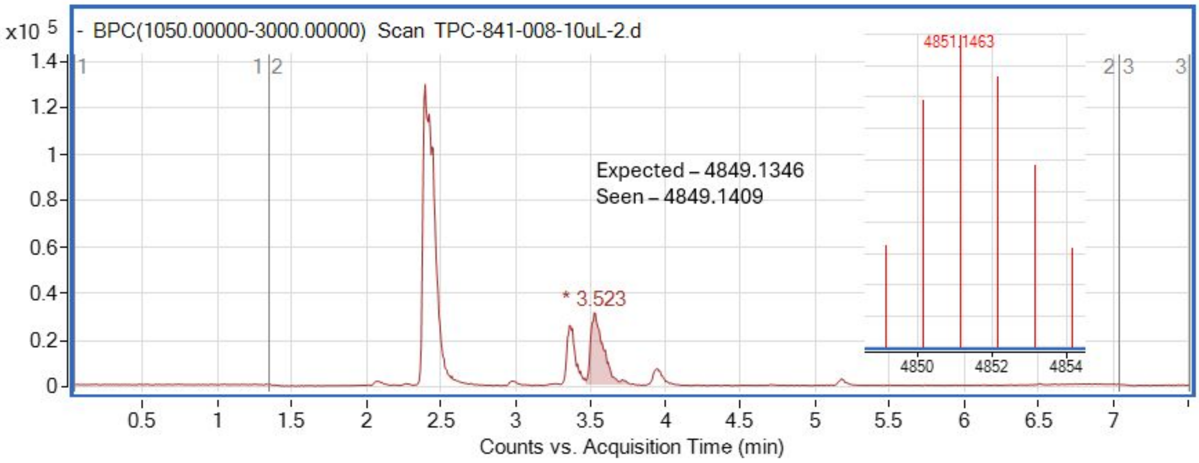

Figure S36 Chromatogram displaying double-stranded DNA product of Buchwald-Hartwig coupling between **HP2** and 2-(piperidin-1-yl)ethan-1-amine.

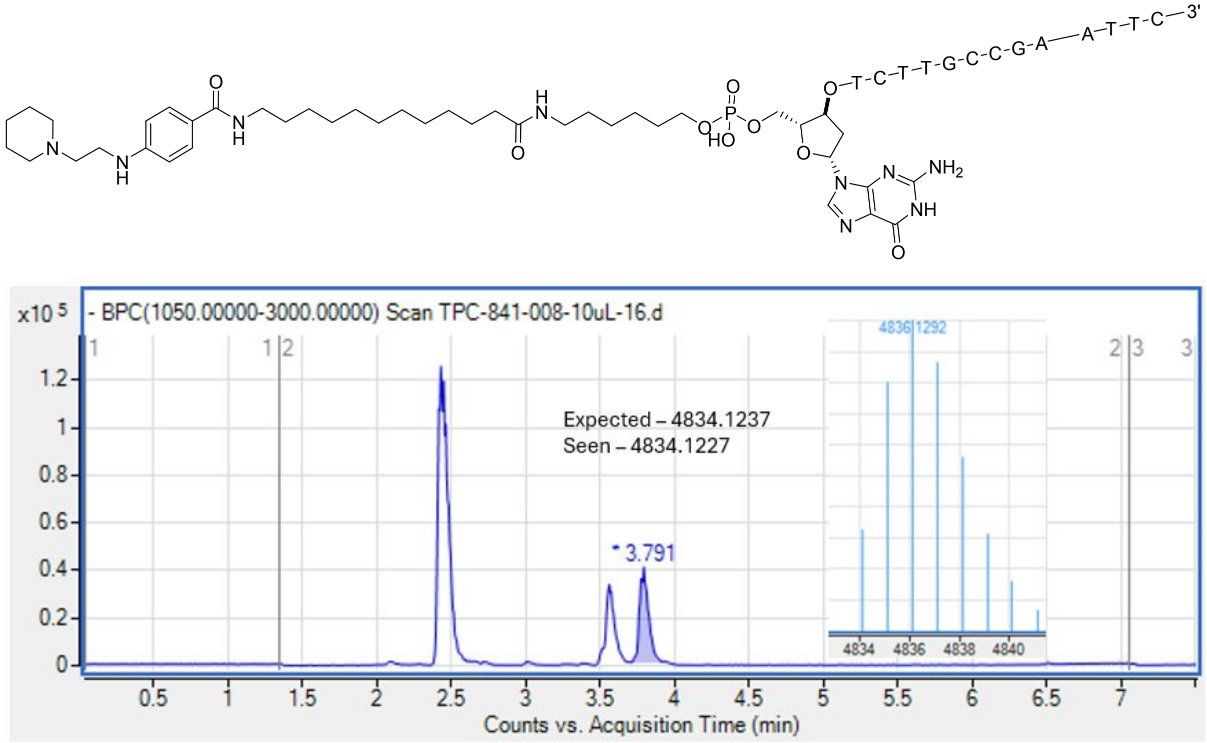

Figure S37 Chromatogram displaying double-stranded DNA product of Buchwald-Hartwig coupling between **HP2** and morpholine.

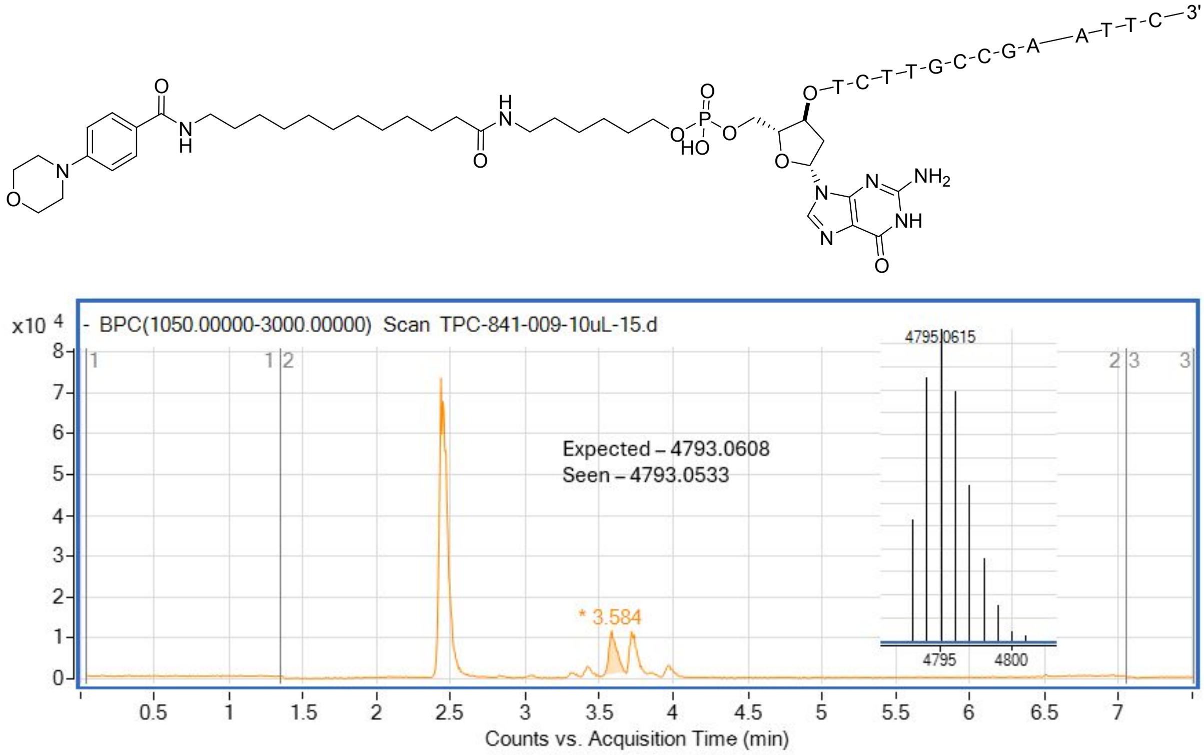

Figure S38 Chromatogram displaying double-stranded DNA product of Buchwald-Hartwig coupling between **HP2** and (*S*)-*N,N*-dimethylpyrrolidin-3-amine.

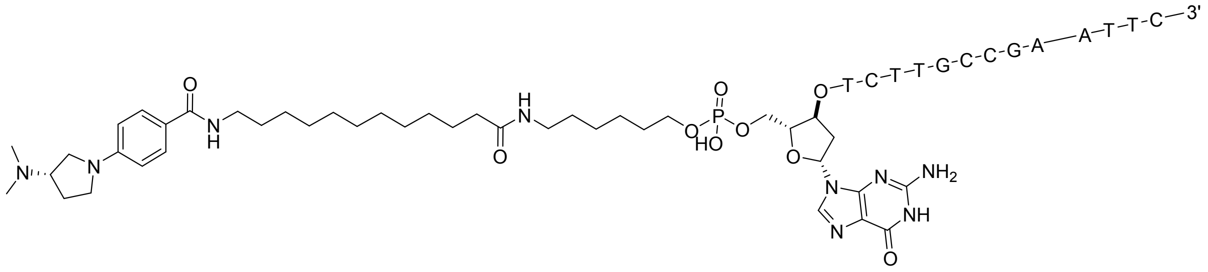

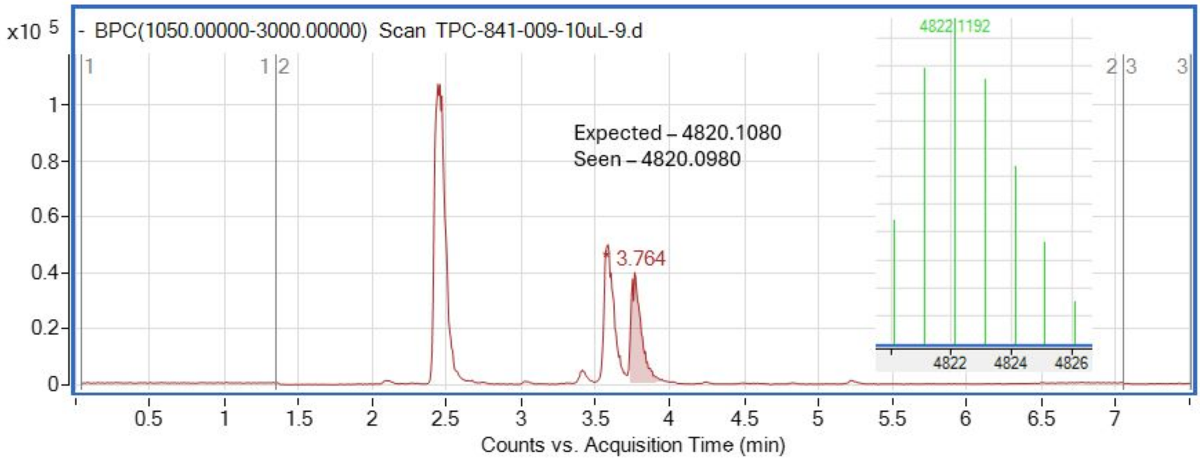

Figure S39 Chromatogram displaying double-stranded DNA product of Buchwald-Hartwig coupling between **HP2** and 3-morpholinopropan-1-amine.

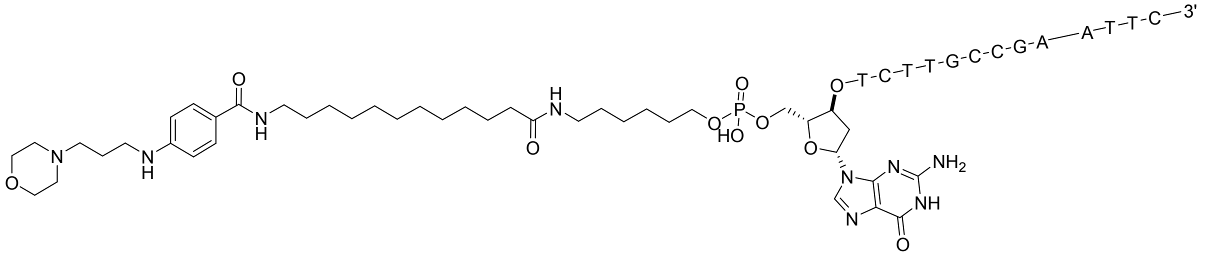

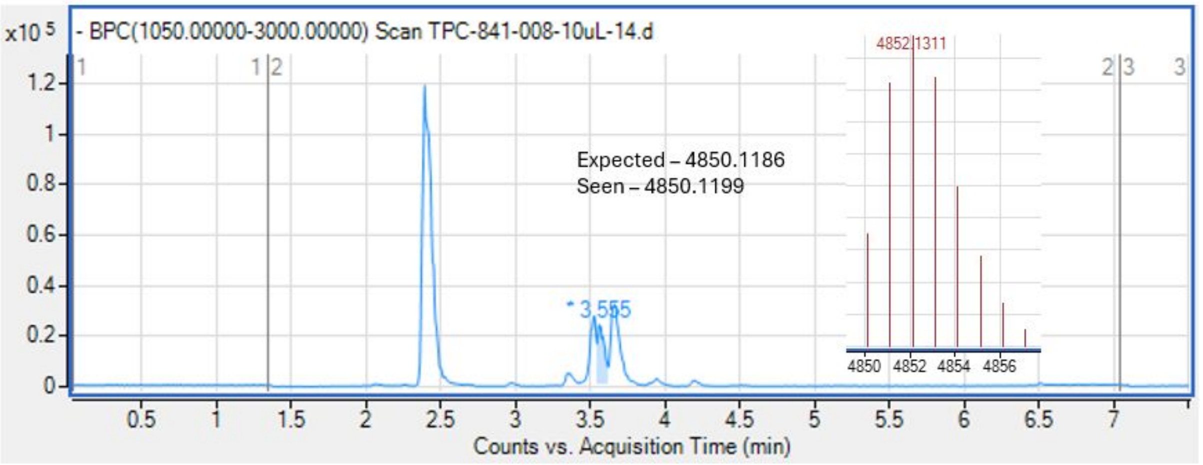

Figure S40 Chromatogram displaying double-stranded DNA product of Buchwald-Hartwig coupling between **HP2** and 2-methoxy-4-morpholinoaniline.

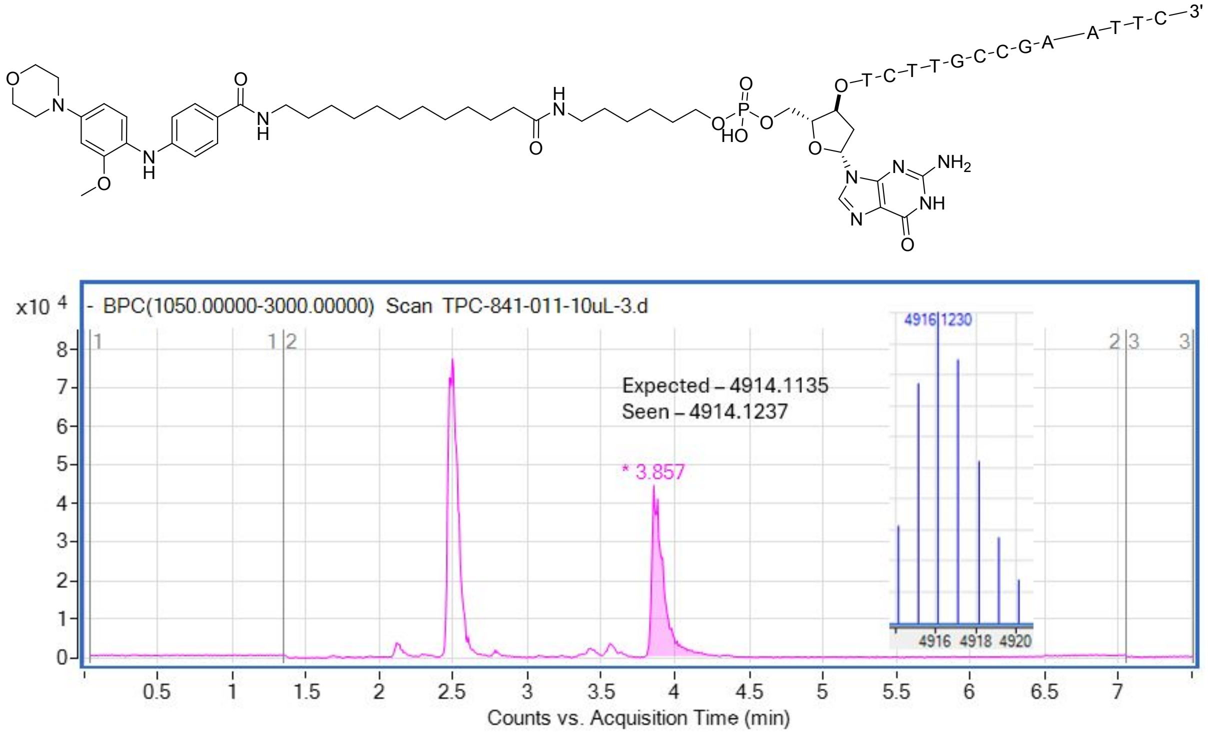

Figure S41 Chromatogram displaying double-stranded DNA product of Buchwald-Hartwig coupling between **HP2** and 1-methyl-1*H*-indol-5-amine.

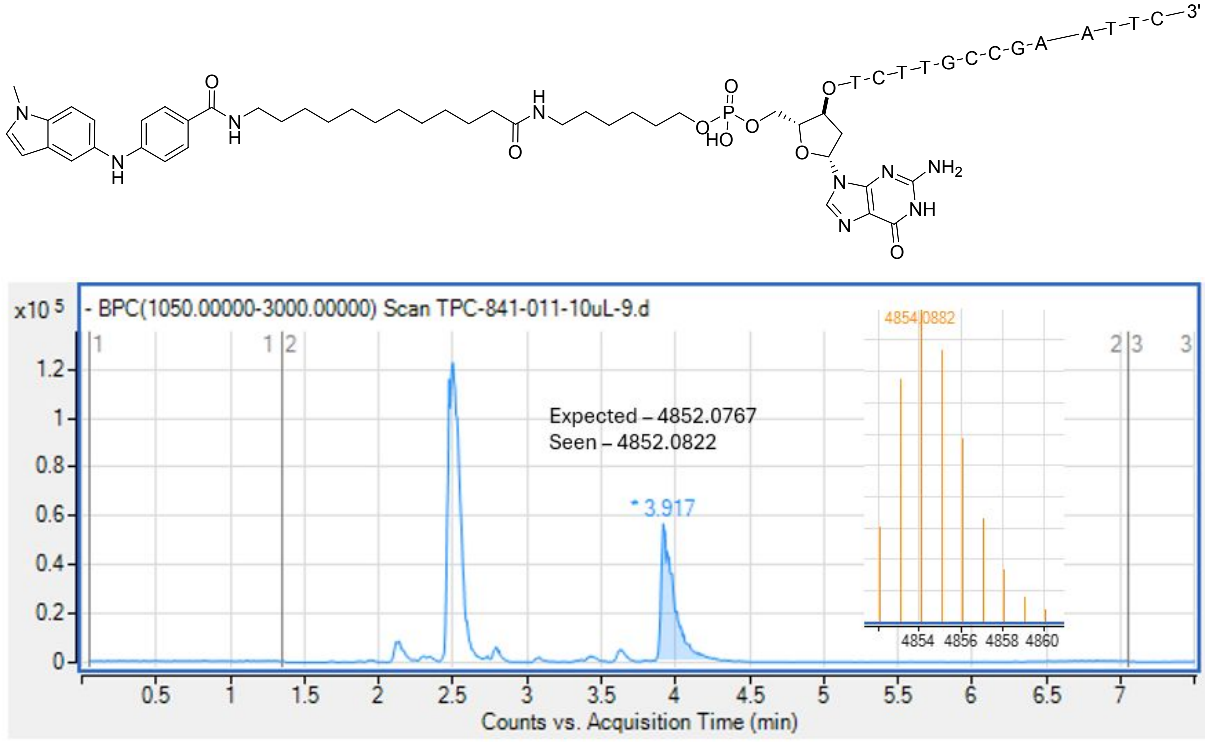

Figure S42 Chromatogram displaying double-stranded DNA product of Buchwald-Hartwig coupling between **HP2** and 1-methyl-1*H*-indazol-6-amine.

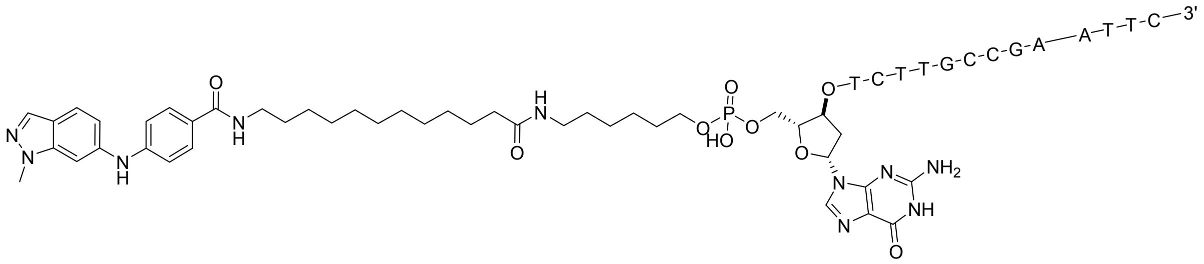

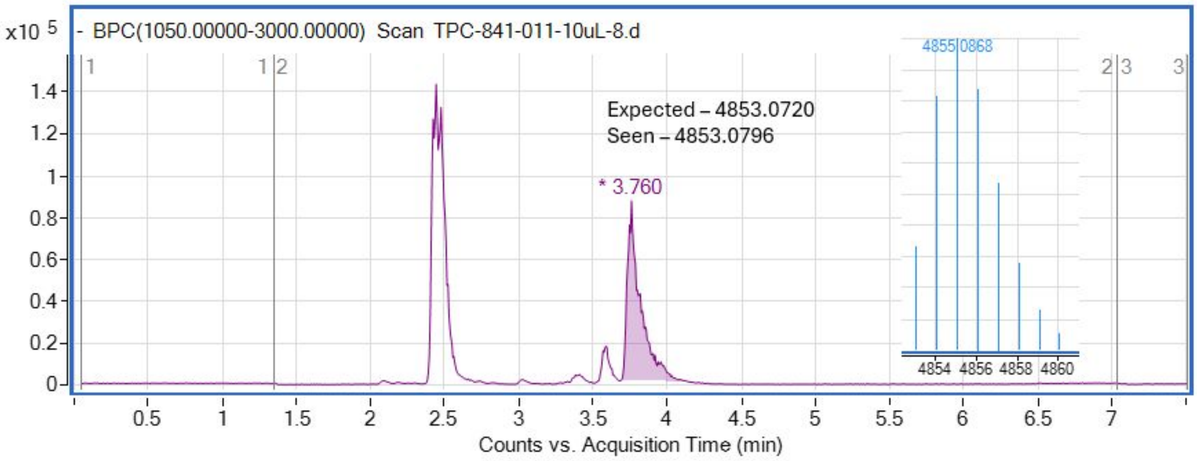

Figure S43 Chromatogram displaying double-stranded DNA product of Buchwald-Hartwig coupling between **HP2** and 2-amino-5-methoxybenzonitrile.

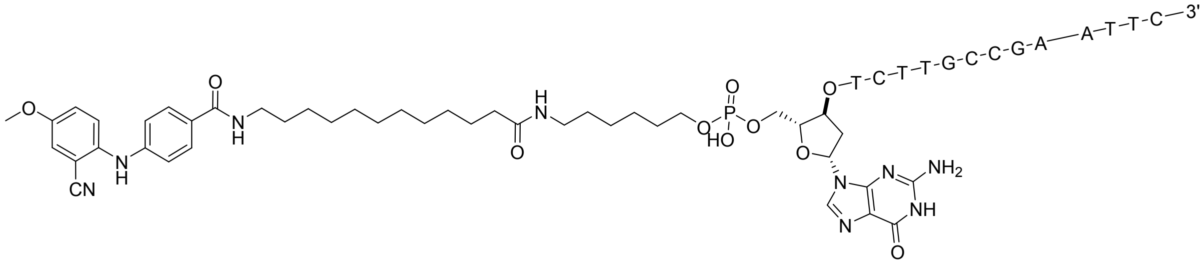

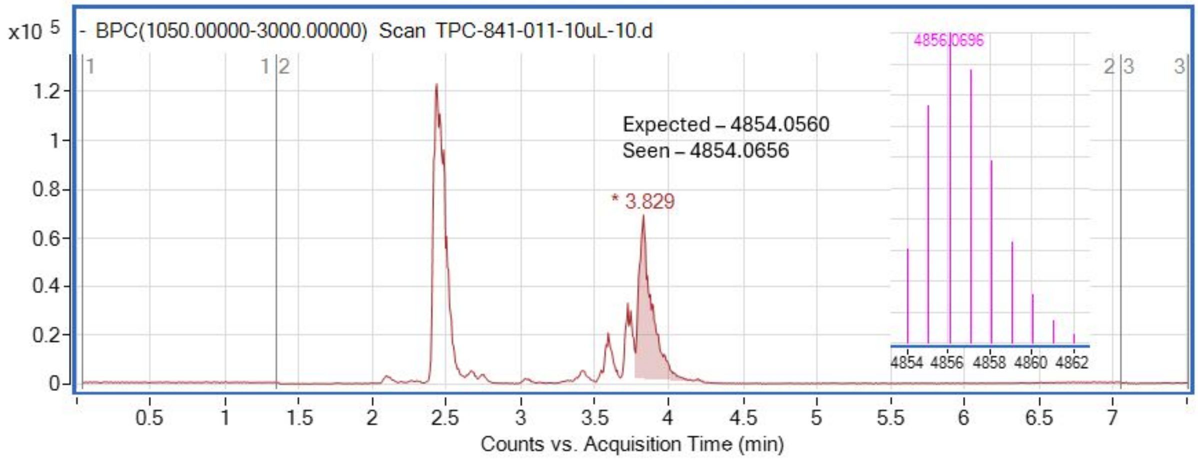

Figure S44 Chromatogram displaying double-stranded DNA product of Buchwald-Hartwig coupling between **HP2** and 2-methoxyaniline.

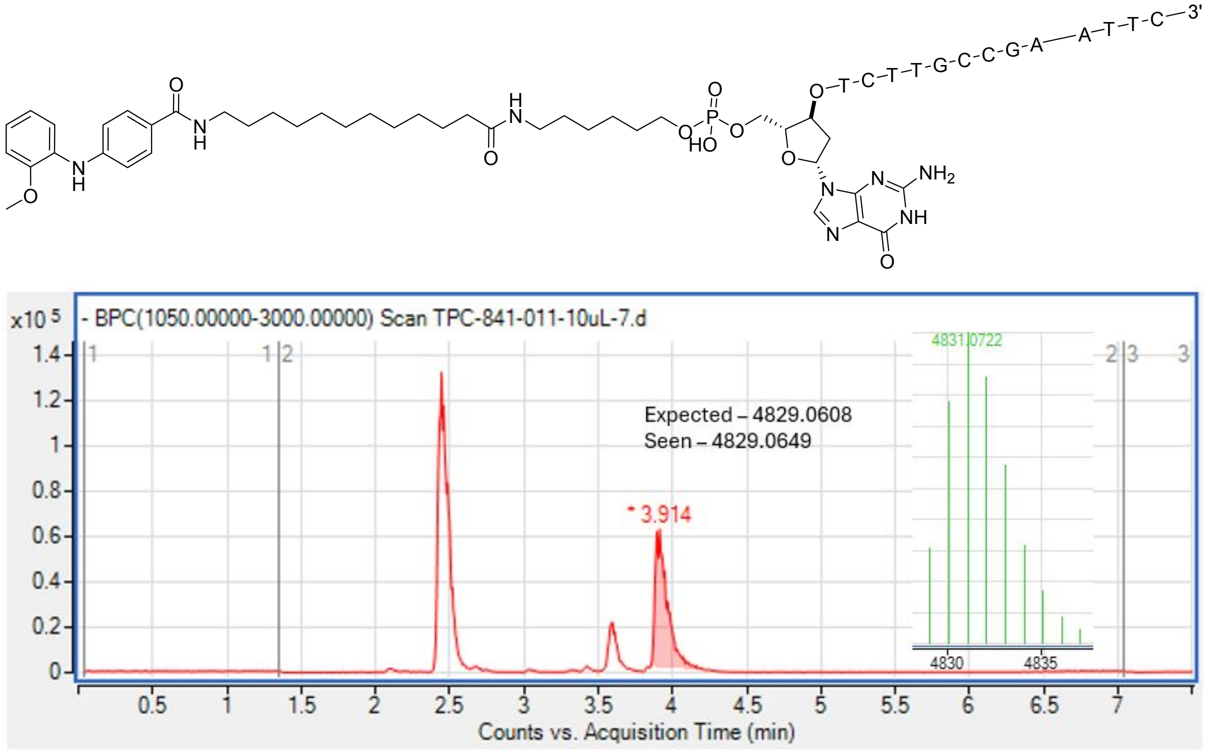

Figure S45 Chromatogram displaying double-stranded DNA product of Buchwald-Hartwig coupling between **HP2** and pyridin-2-amine.

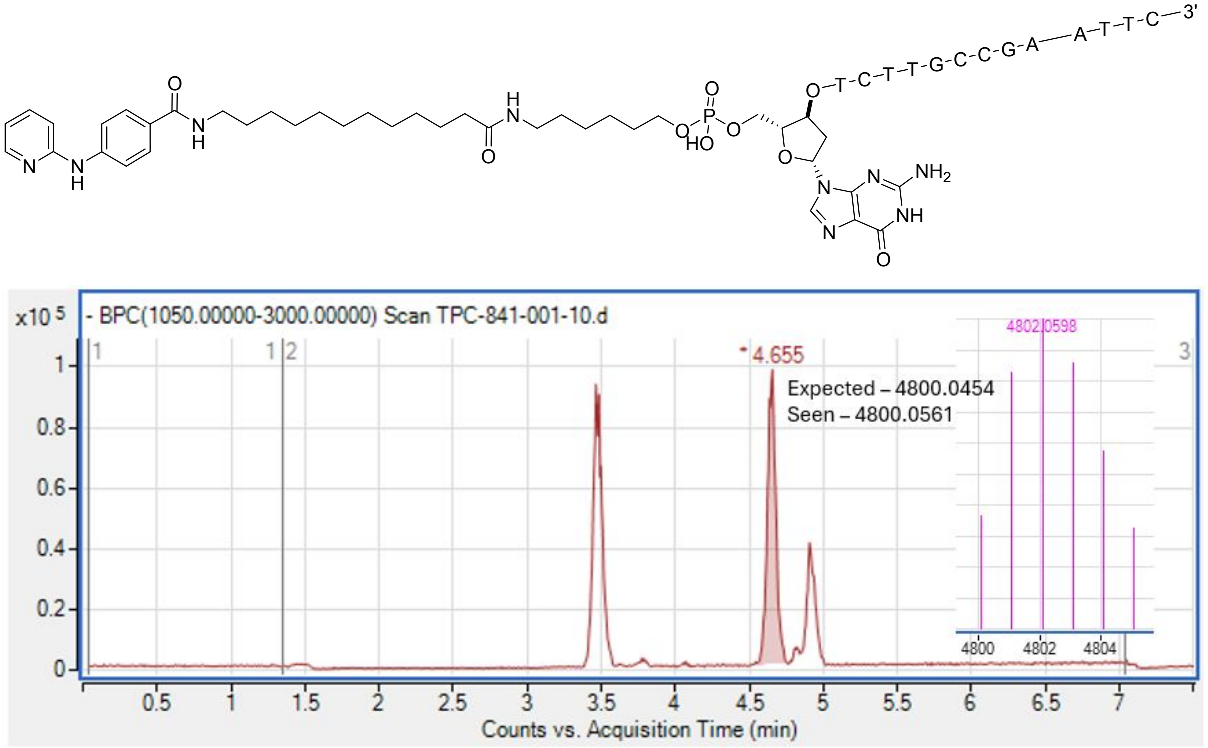

Figure S46 Chromatogram displaying double-stranded DNA product of Buchwald-Hartwig coupling between **HP2** and 2-(4-aminophenyl)acetonitrile.

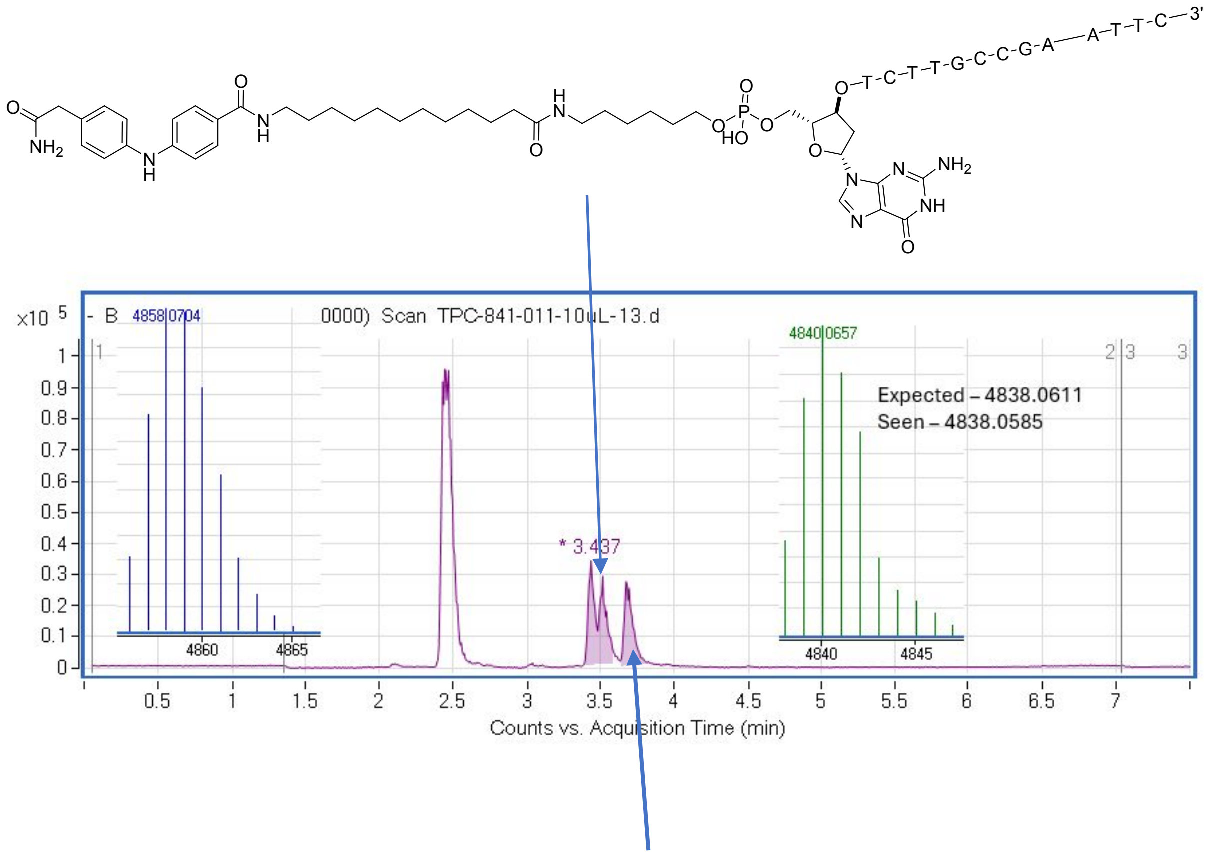

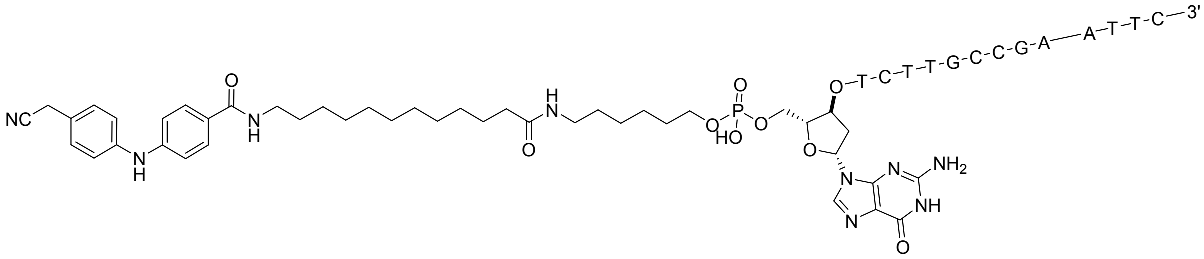

Figure S47 Chromatogram displaying double-stranded DNA product of Buchwald-Hartwig coupling between **HP2** and indoline.

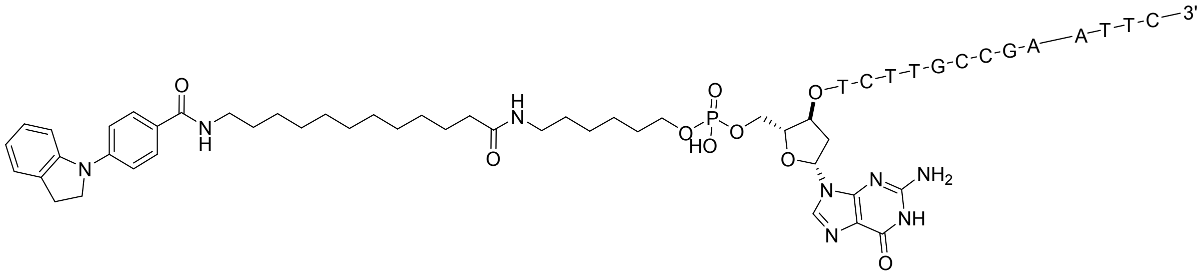

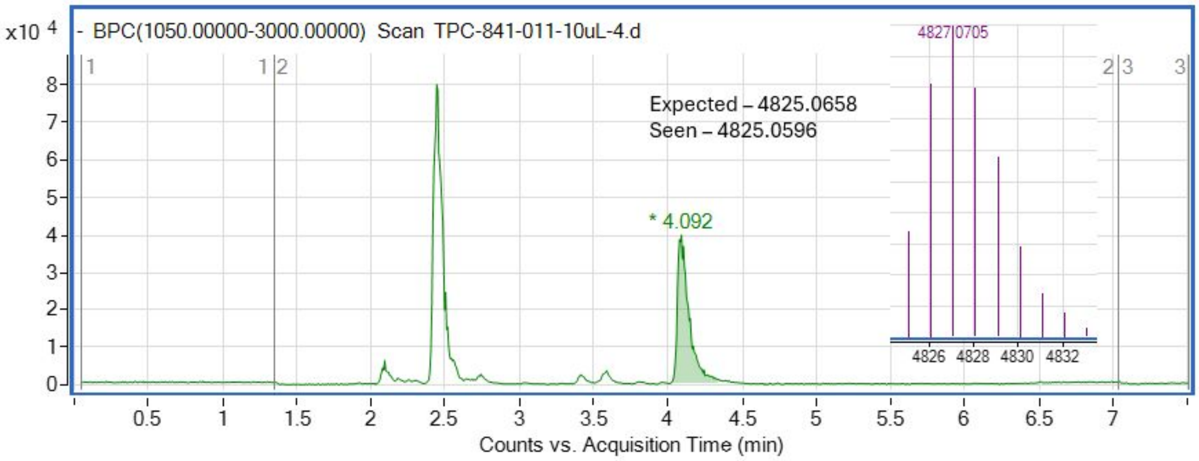

Figure S48 Chromatogram displaying double-stranded DNA product of Buchwald-Hartwig coupling between **HP2** and 2-phenylacetamide.

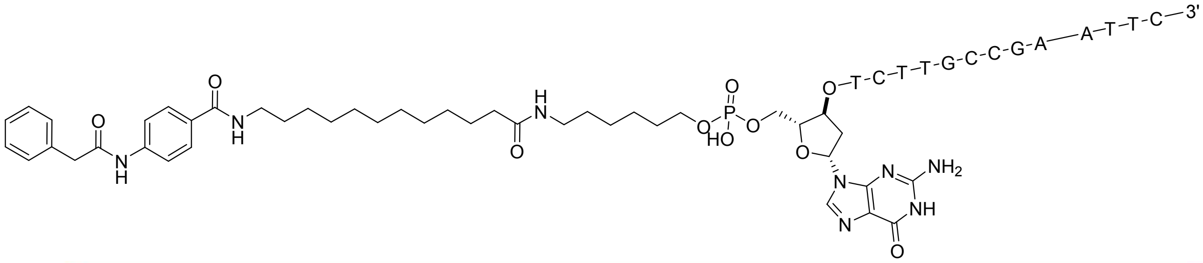

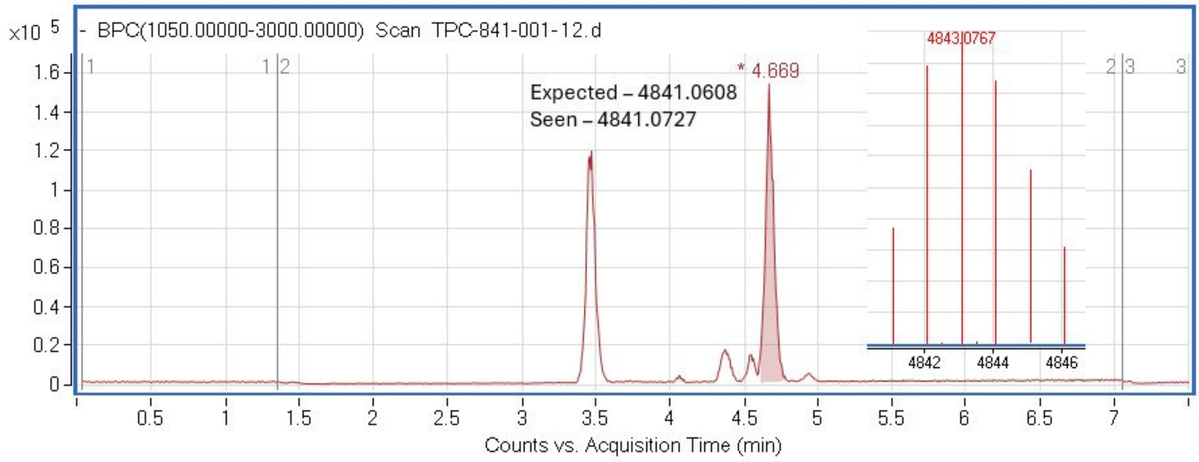

Figure S49 Chromatogram displaying double-stranded DNA product of Buchwald-Hartwig coupling between **HP2** and 1-(3-methoxyphenyl)piperazine.

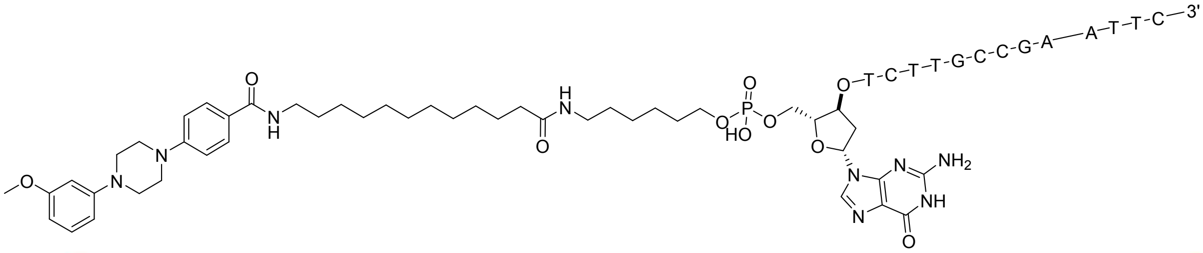

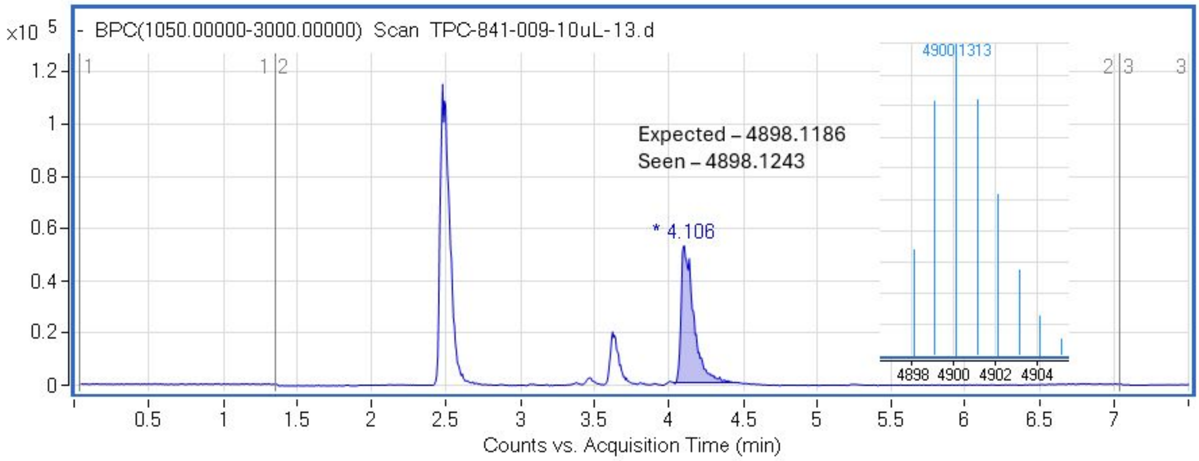

Figure S50 Chromatogram displaying double-stranded DNA product of Buchwald-Hartwig coupling between **HP2** and 1-(*o*-tolyl)urea.

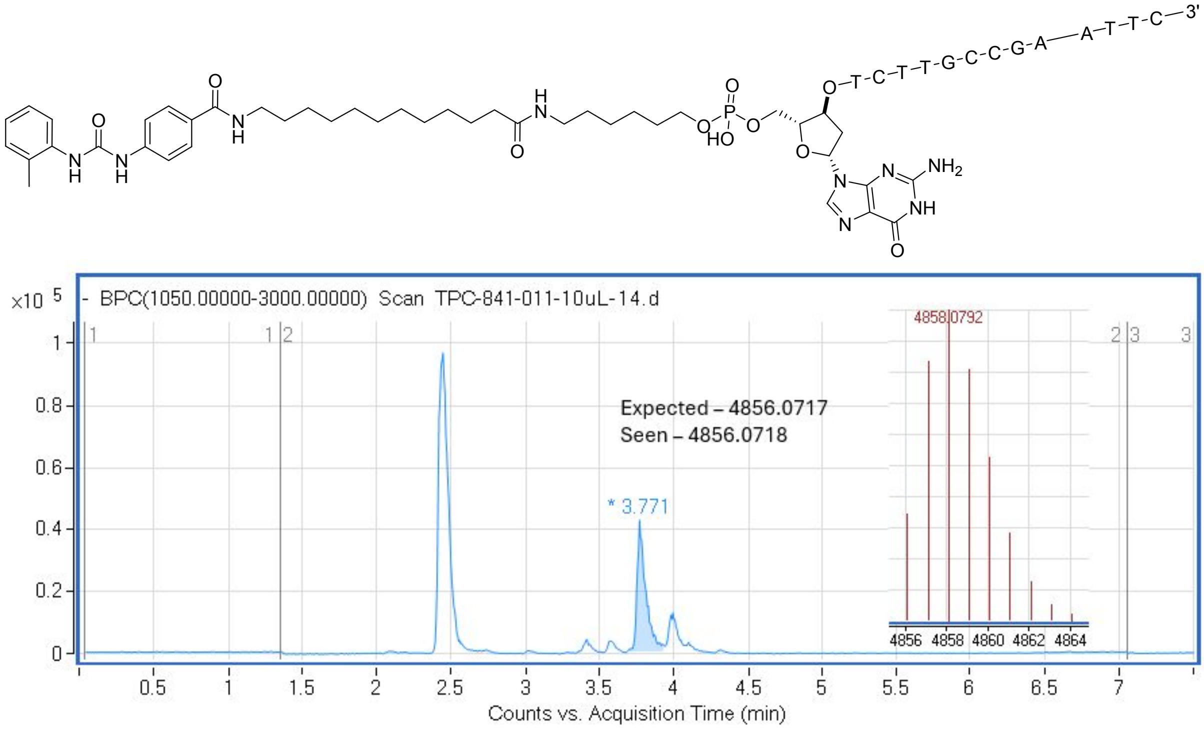

Figure S51 Chromatogram displaying double-stranded DNA product of Buchwald-Hartwig coupling between **HP2** and 1-(4-fluorobenzyl)urea.

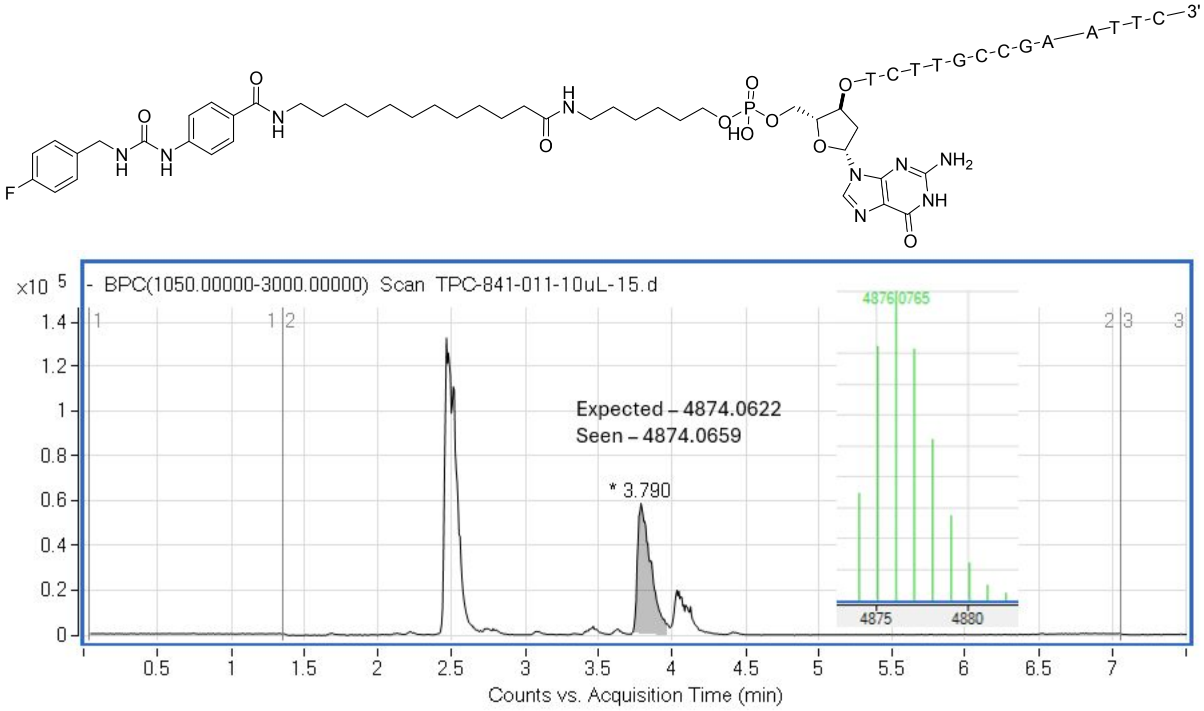

Figure S52 Chromatogram displaying double-stranded DNA product of Buchwald-Hartwig coupling between **HP2** and 1,2,3,4-tetrahydroisoquinoline.

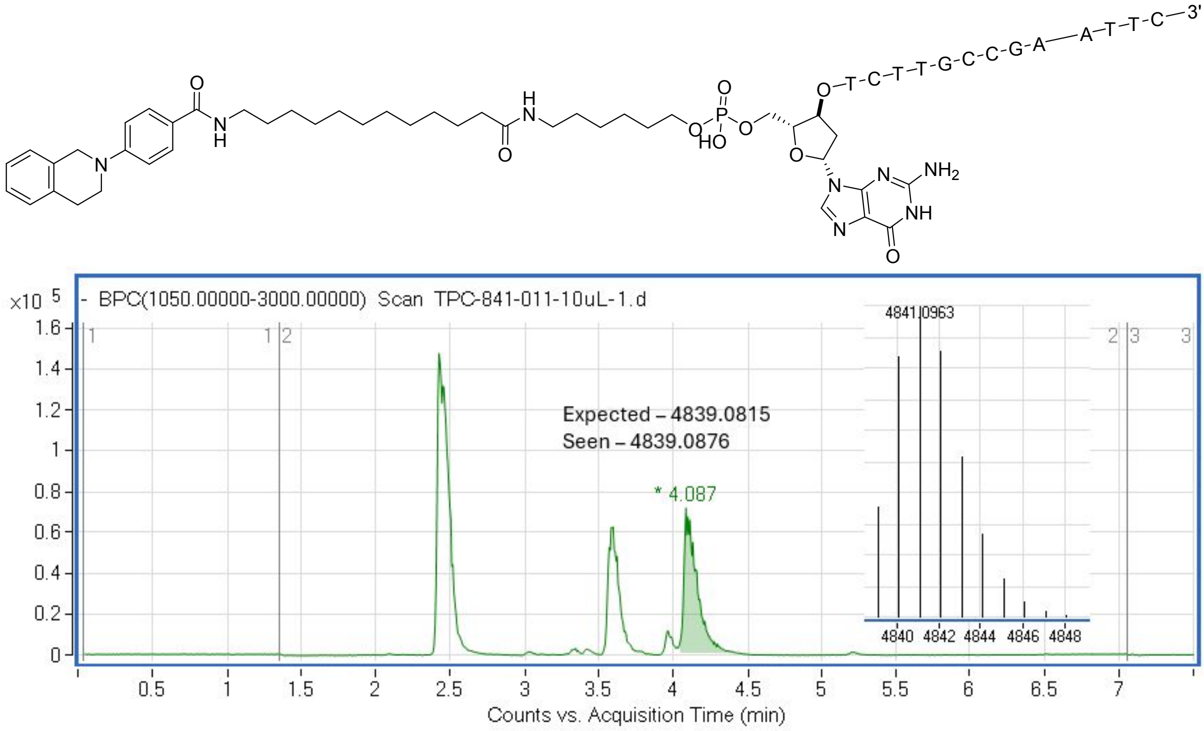

Figure S53 Chromatogram displaying double-stranded DNA product of Buchwald-Hartwig coupling between **HP2** and 1-(2-fluorophenyl)piperazine.

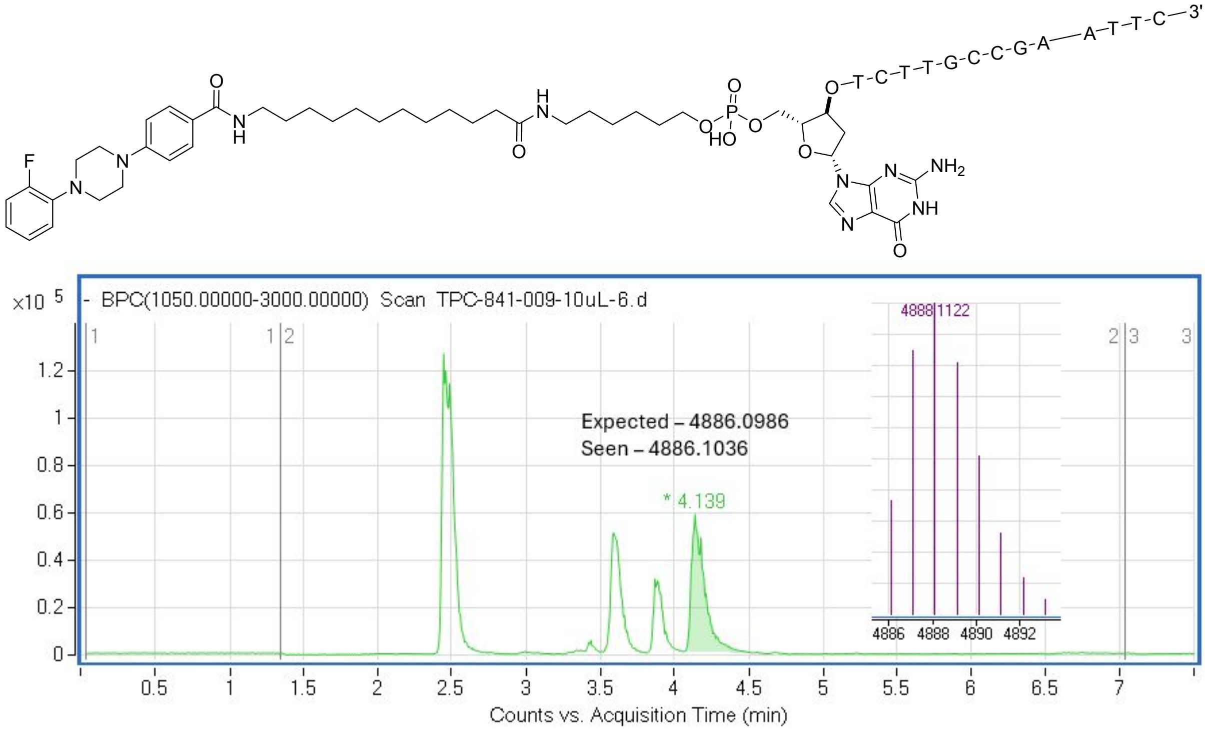

On-DNA Buchwald-Hartwig Amination with DNA-Conjugated Aryl Halides (Table 7)

Synthesis and Characterisation of DNA Headpieces for Table 7

DNA headpieces were prepared from **greasy amine DNA HP** according to the general on-DNA DMT-MM forward amide coupling procedure.

Figure S54 Chromatogram displaying double-stranded DNA headpiece for Buchwald-Hartwig reactions, comprising both the complementary strand and 4-bromophenyl-conjugated DNA-greasy amine linker.

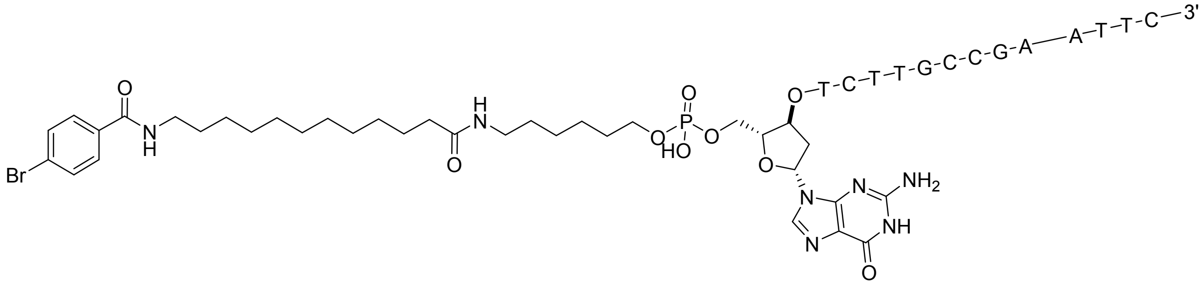

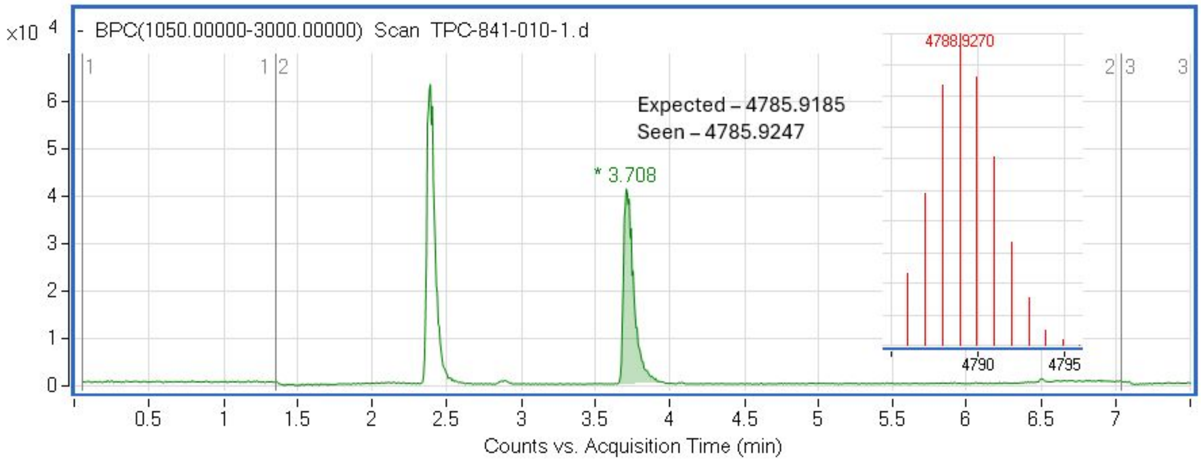

Figure S55 Chromatogram displaying double-stranded DNA headpiece for Buchwald-Hartwig reactions, comprising both the complementary strand and 5-iodopicolinate-conjugated DNA-greasy amine linker.

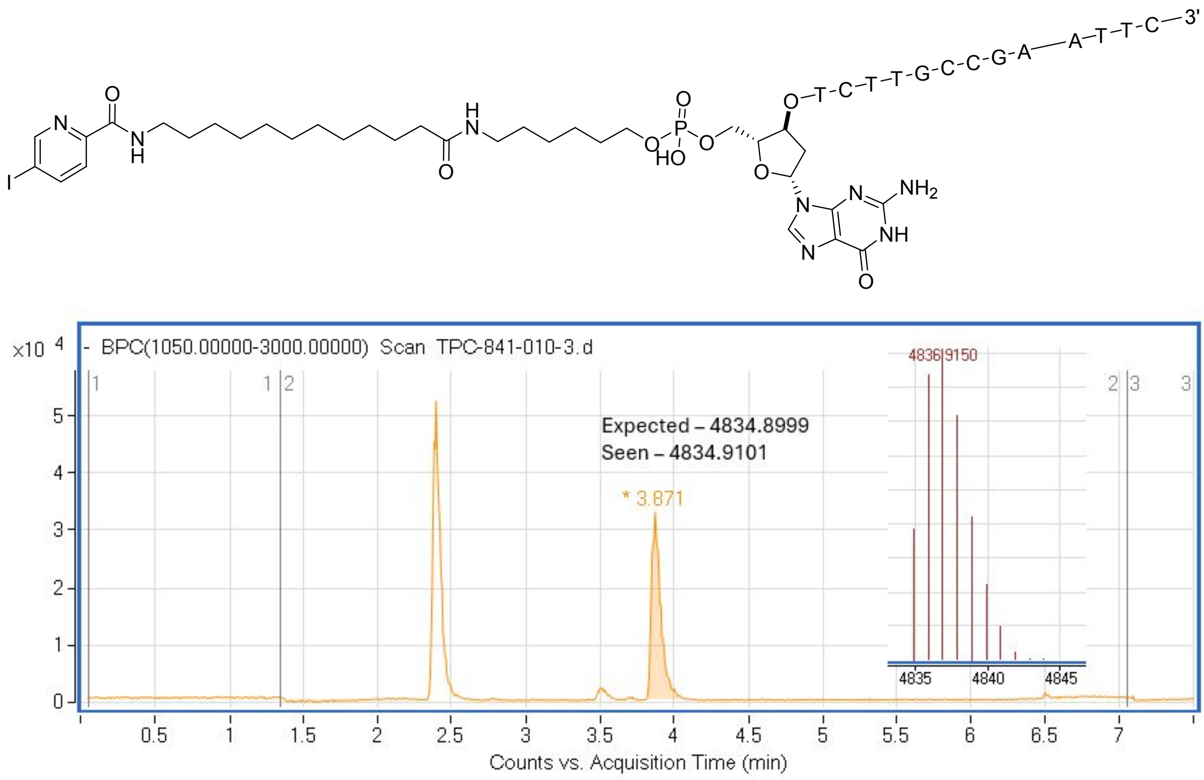

Figure S56 Chromatogram displaying double-stranded DNA headpiece for Buchwald-Hartwig reactions, comprising both the complementary strand and 3-iodophenyl-conjugated DNA-greasy amine linker.

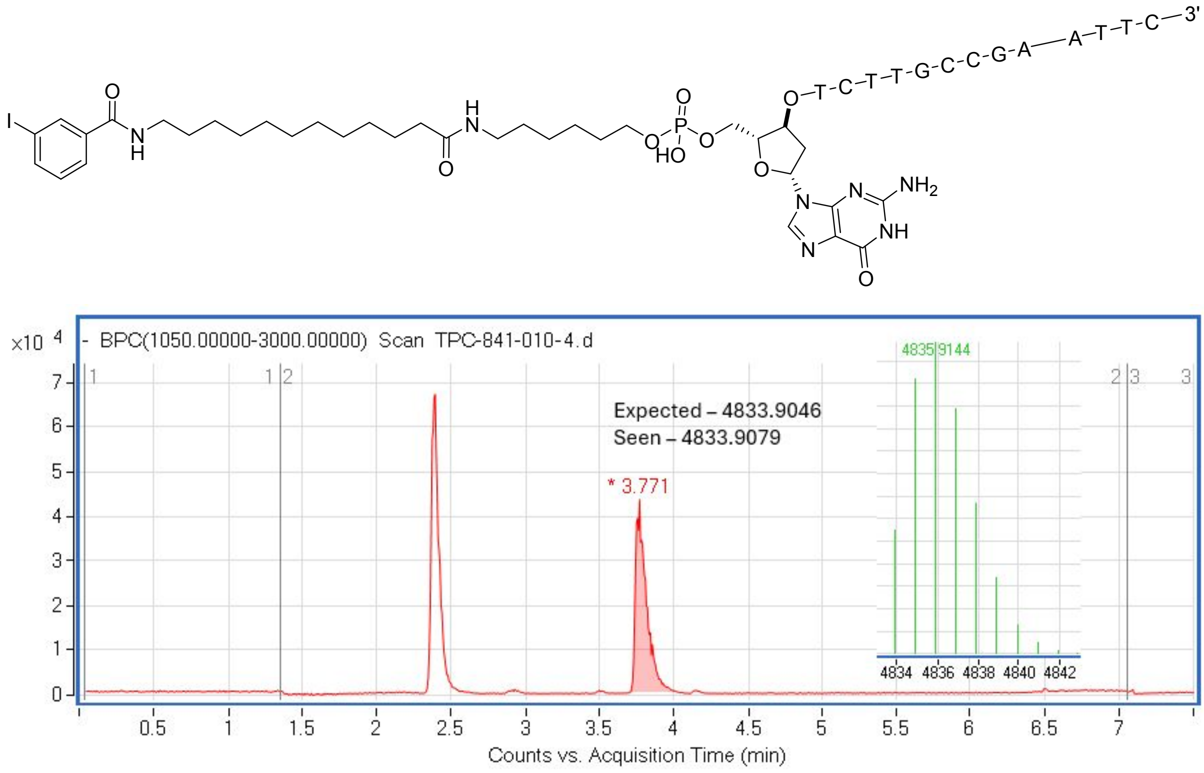

Figure S57 Chromatogram displaying double-stranded DNA headpiece for Buchwald-Hartwig reactions, comprising both the complementary strand and 2-(4-iodophenyl)acetate-conjugated DNA-greasy amine linker.

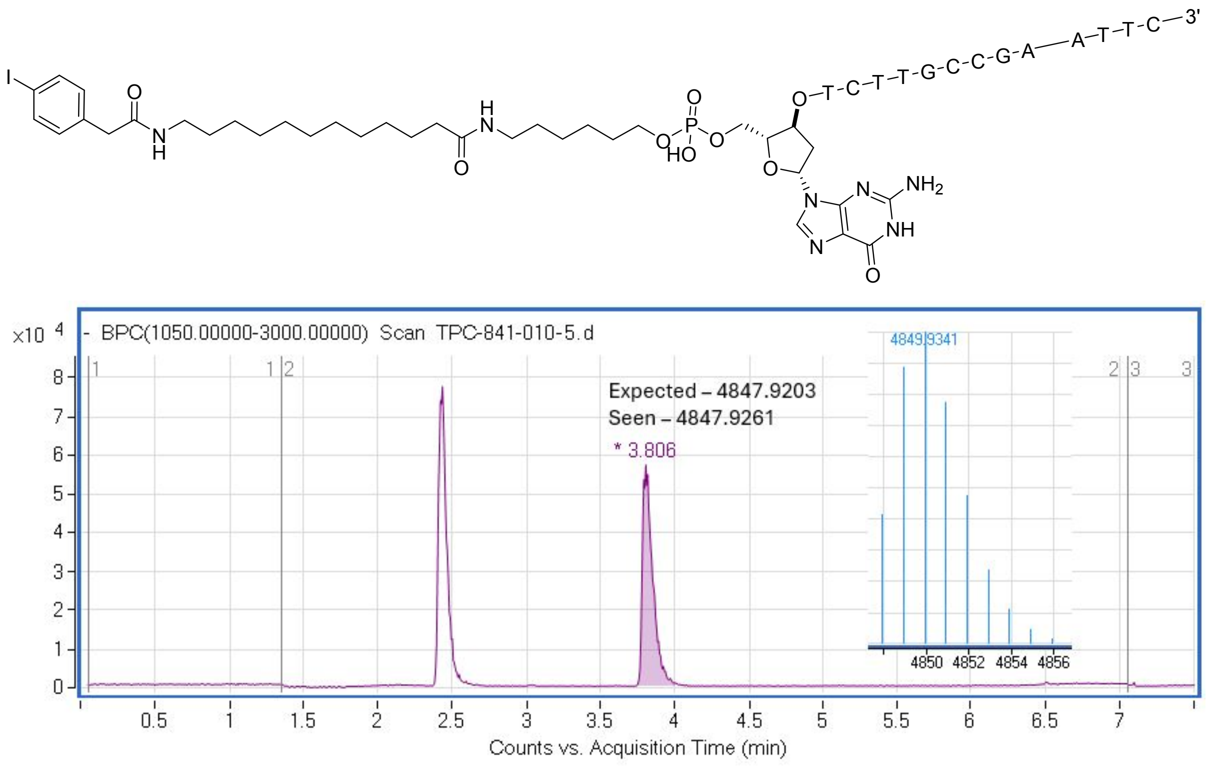

Figure S58 Chromatogram displaying double-stranded DNA headpiece for Buchwald-Hartwig reactions, comprising both the complementary strand and 5-bromo-2-(trifluoromethyl)phenyl-conjugated DNA-greasy amine linker.

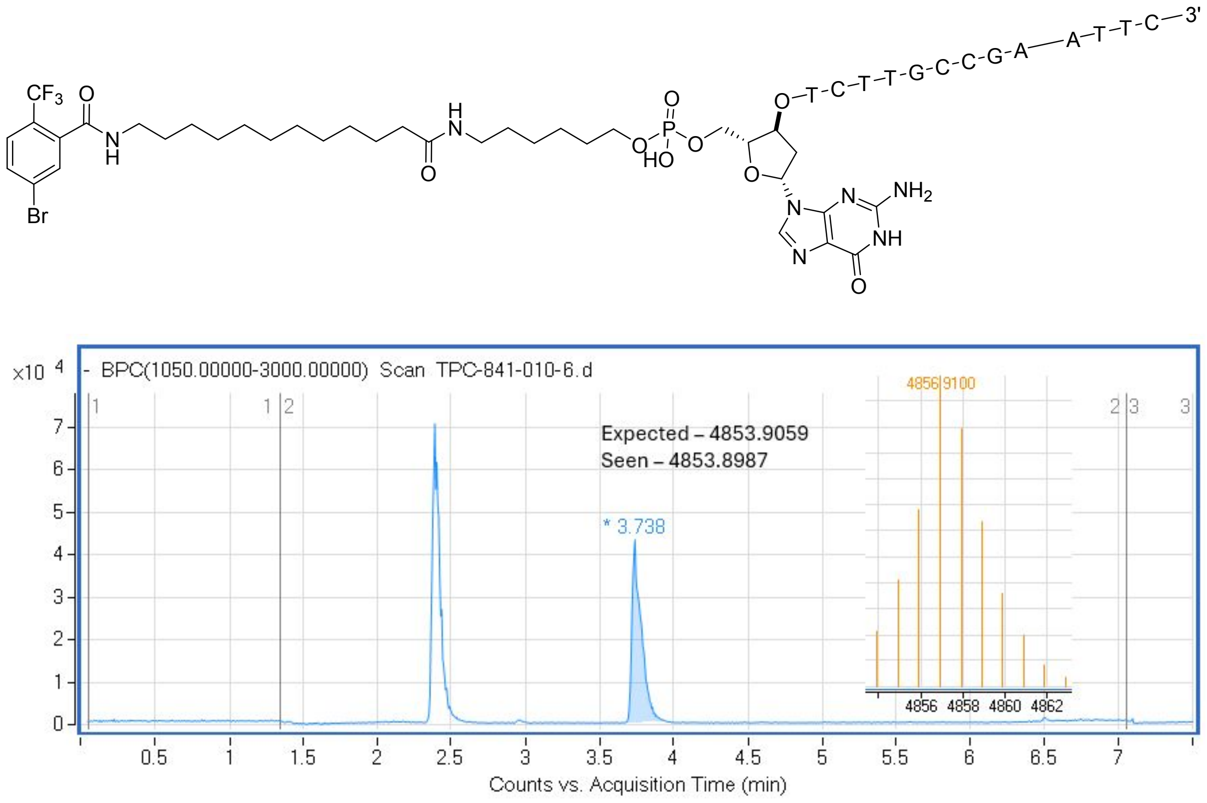

Figure S59 Chromatogram displaying double-stranded DNA headpiece for Buchwald-Hartwig reactions, comprising both the complementary strand and 4-bromo-2-fluorophenyl-conjugated DNA-greasy amine linker.

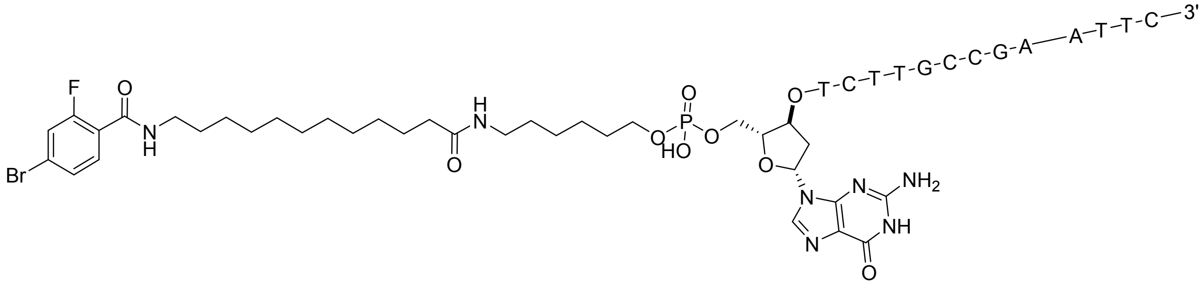

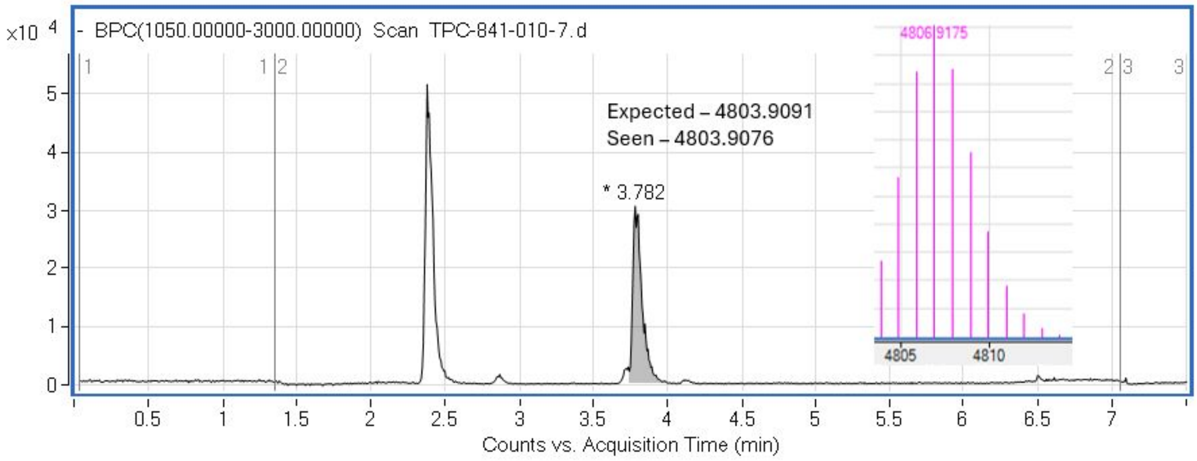

6x6 Buchwald-Hartwig Amination (Table 7) Chromatograms

Span 60 (20 µL, 5% v/v in THF) was added to 0.5 mL Eppendorf™ Polypropylene DNA LoBind Tube and concentrated using a Genevac™ Concentrator EZ-2 Plus at 50 °C for 2 h. Upon removal of THF, DEPC-treated water (20 µL). Double-stranded DNA (4 µL, 1 nmol), amine (9 µmol), and *t*-BuOK (6 µL, 5 M in H_2O) were added to the Eppendorf and vortexed for 30s. [(Crotyl)PdCl]$_2$ (3 µL, 81 mM in THF) and *t*-BuXPhos (3 µL, 162 mM in THF) were added and the tube vortexed for 30 s. The sample was heated using a STARLAB® Thermomixer-Mixer HC at 1200 rpm at 70 °C for 1 h. After this time the reaction was allowed to cool, then sodium diethyldithiocarbamic acid (6 µL, 1 M in H_2O) was added and the reaction was heated at 60 ˚C for a further 30 mins. The reaction mixture was allowed to cool, diluted with DEPC-treated water (2 x 100 µL) and filtered through a hydrophilic PTFE syringe filter. The reaction was then precipitated according to the general ethanol precipitation procedure and analysed by mass spectrometry.

Figure S60 Chromatogram displaying double-stranded DNA product of Buchwald-Hartwig coupling between 4-bromophenyl-conjugated DNA-greasy amine linker and phenylmethanamine.

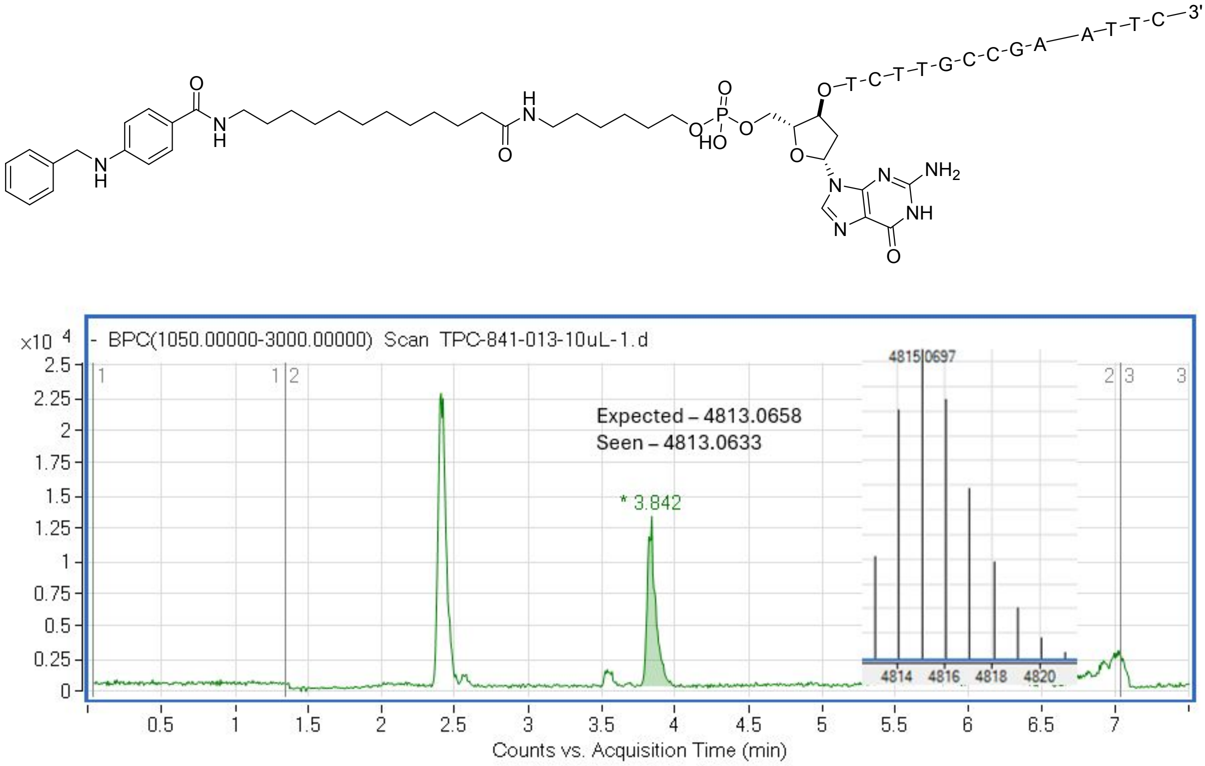

Figure S61 Chromatogram displaying double-stranded DNA product of Buchwald-Hartwig coupling between 4-bromophenyl-conjugated DNA-greasy amine linker and (2-methoxypyridin-4-yl)methanamine.

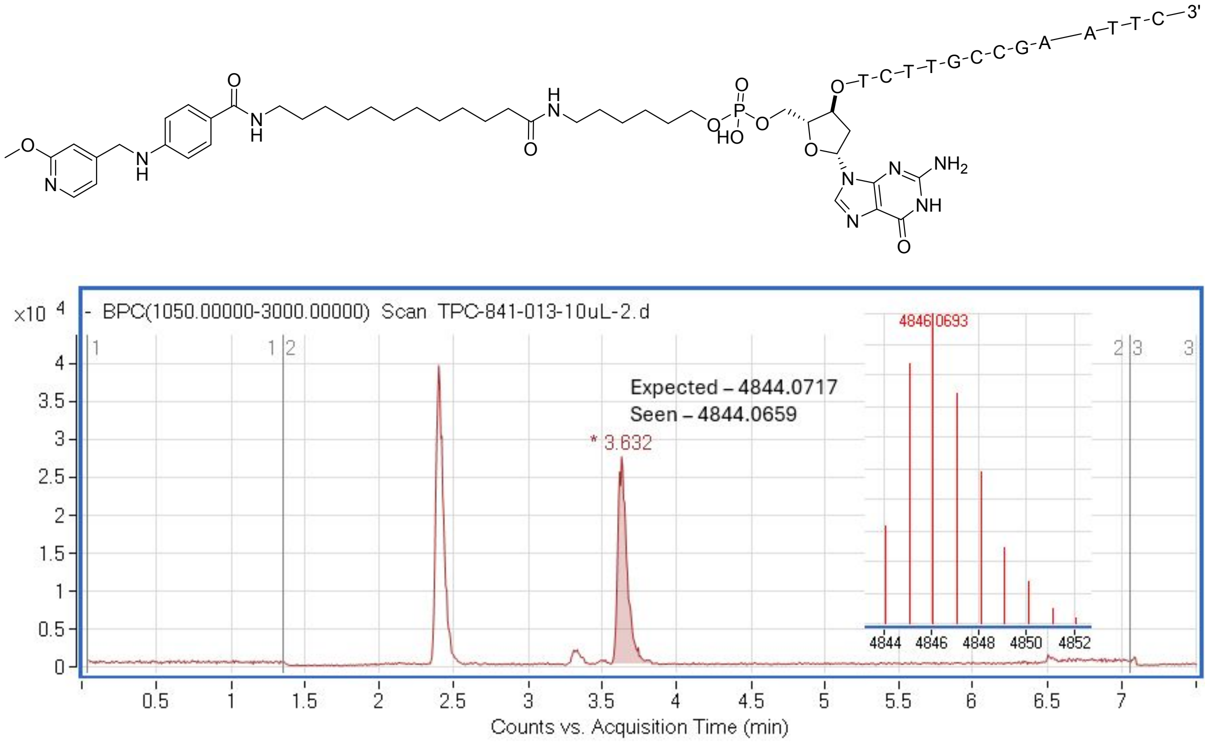

Figure S62 Chromatogram displaying double-stranded DNA product of Buchwald-Hartwig coupling between 4-bromophenyl-conjugated DNA-greasy amine linker and cyclohexylmethanamine.

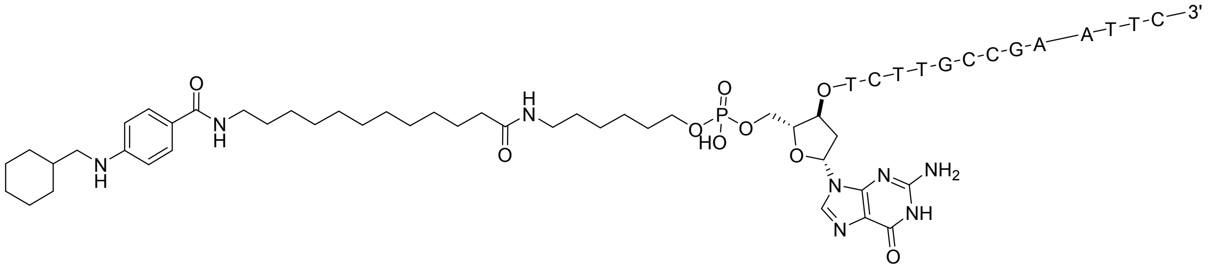

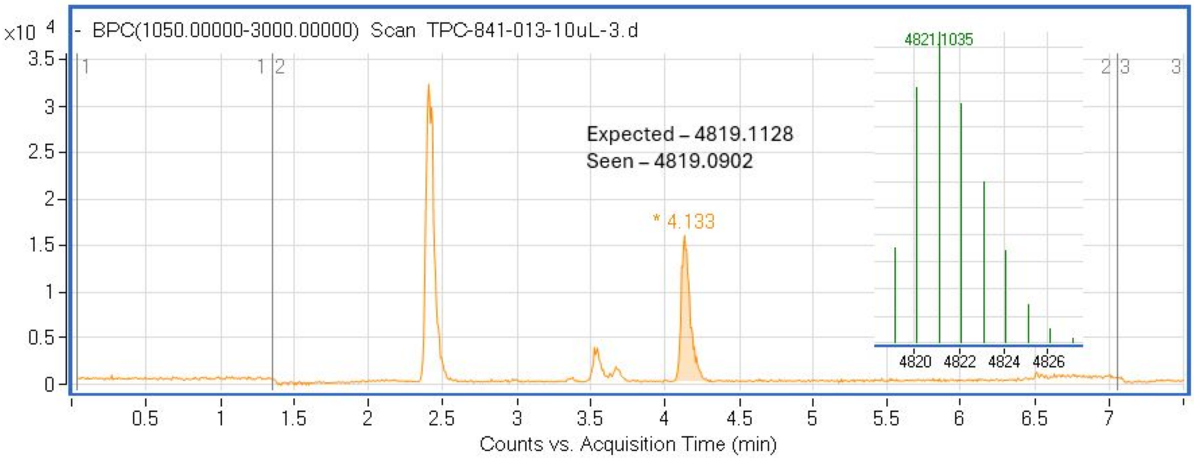

Figure S63 Chromatogram displaying double-stranded DNA product of Buchwald-Hartwig coupling between 4-bromophenyl-conjugated DNA-greasy amine linker and piperidine.

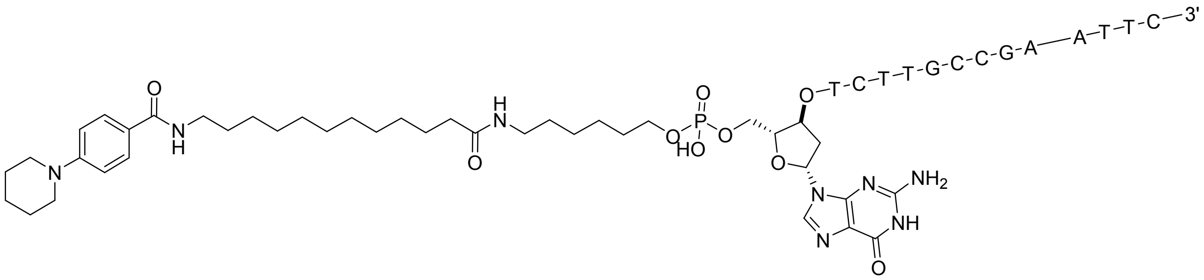

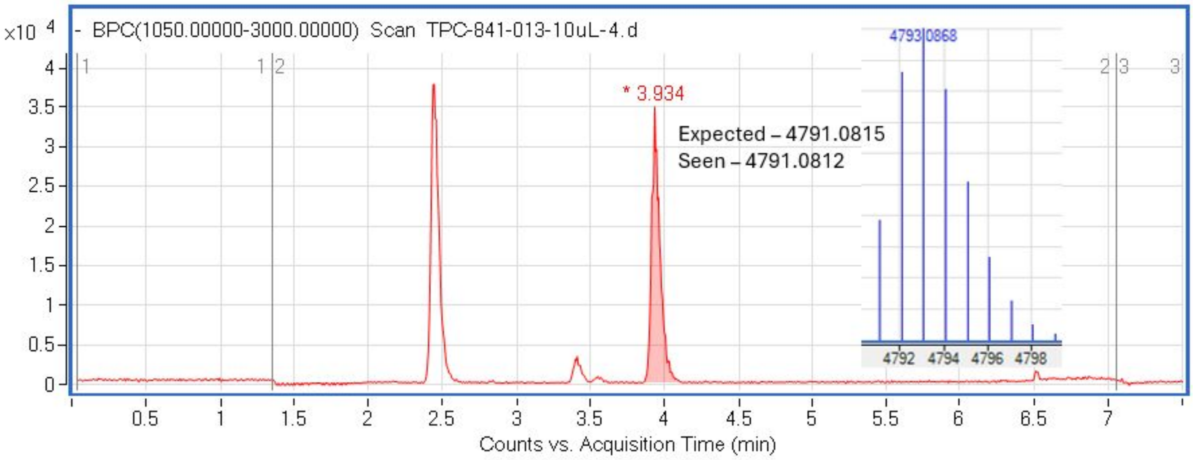

Figure S64 Chromatogram displaying double-stranded DNA product of Buchwald-Hartwig coupling between 4-bromophenyl-conjugated DNA-greasy amine linker and *N*-methyl-1-phenylmethanamine.

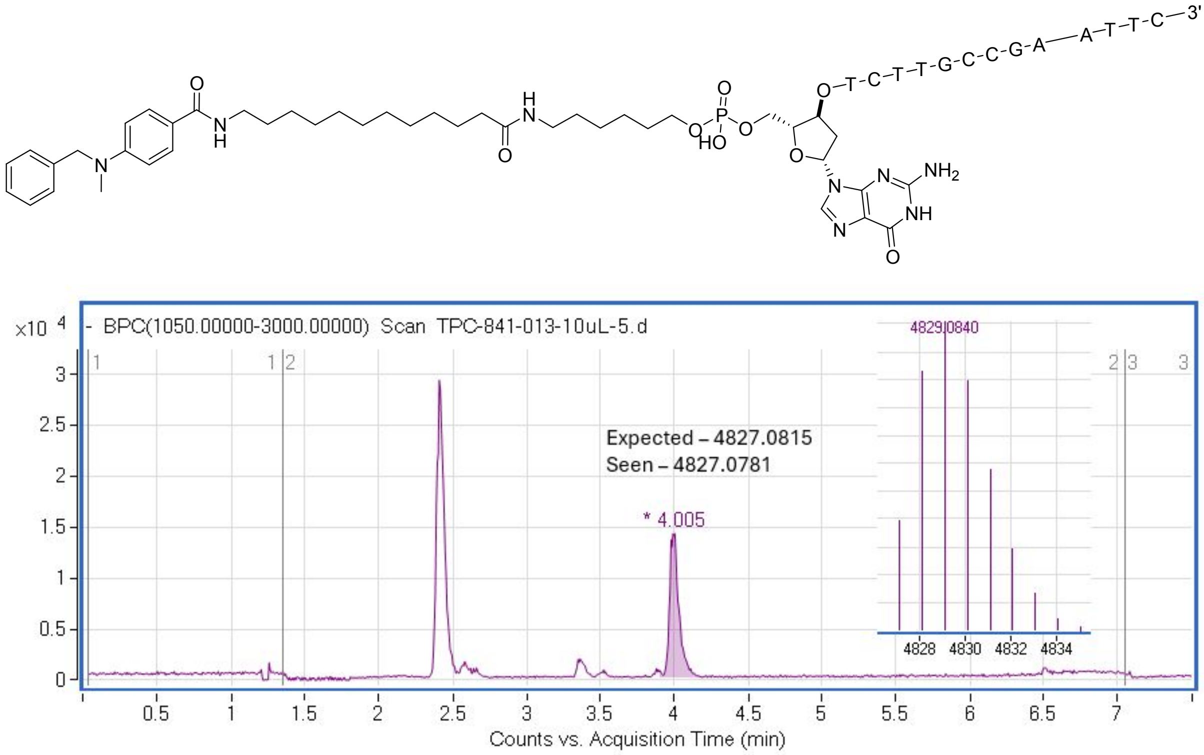

Figure S65 Chromatogram displaying double-stranded DNA product of Buchwald-Hartwig coupling between 5-iodopicolinate-conjugated DNA-greasy amine linker and phenylmethanamine.

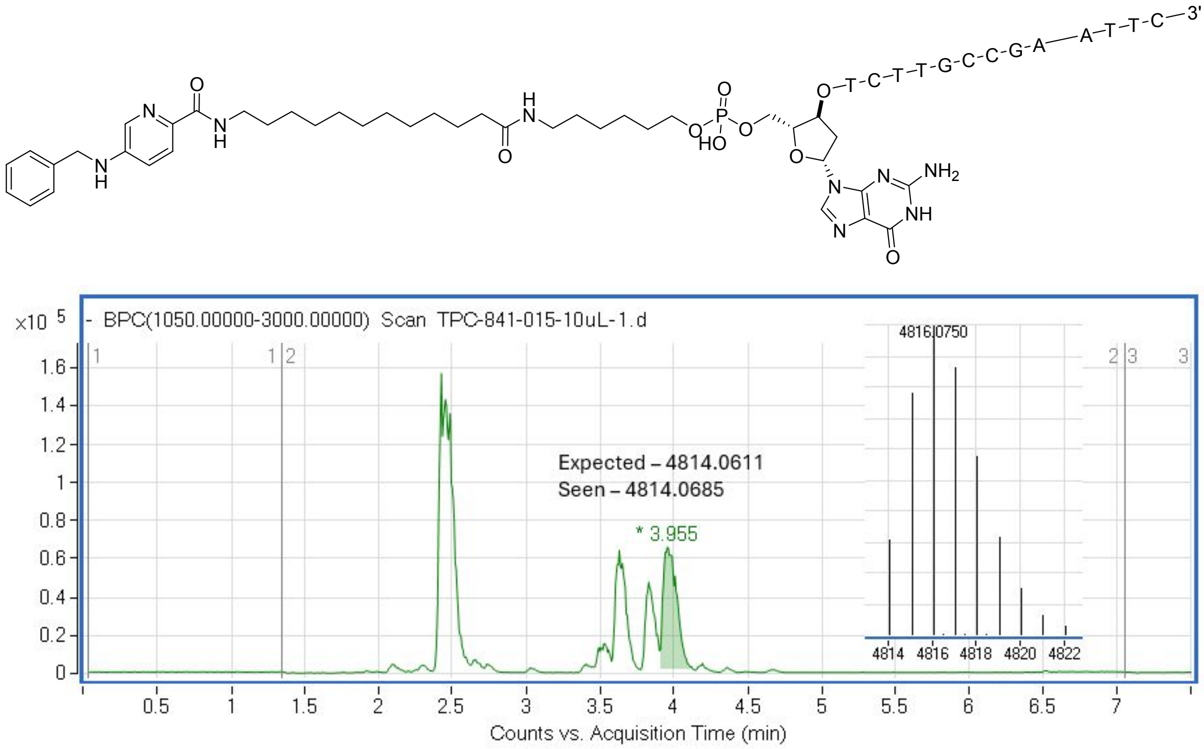

Figure S66 Chromatogram displaying double-stranded DNA product of Buchwald-Hartwig coupling between 5-iodopicolinate-conjugated DNA-greasy amine linker and (2-methoxypyridin-4-yl)methanamine.

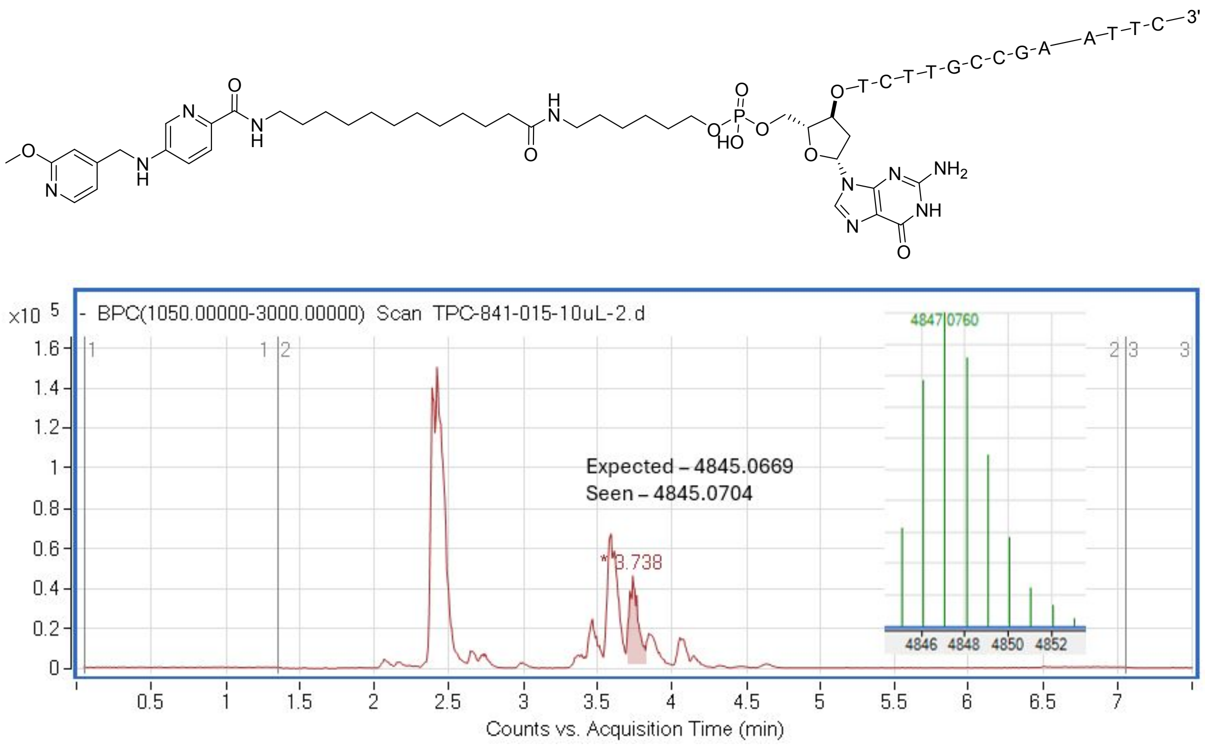

Figure S67 Chromatogram displaying double-stranded DNA product of Buchwald-Hartwig coupling between 5-iodopicolinate-conjugated DNA-greasy amine linker and cyclohexylmethanamine.

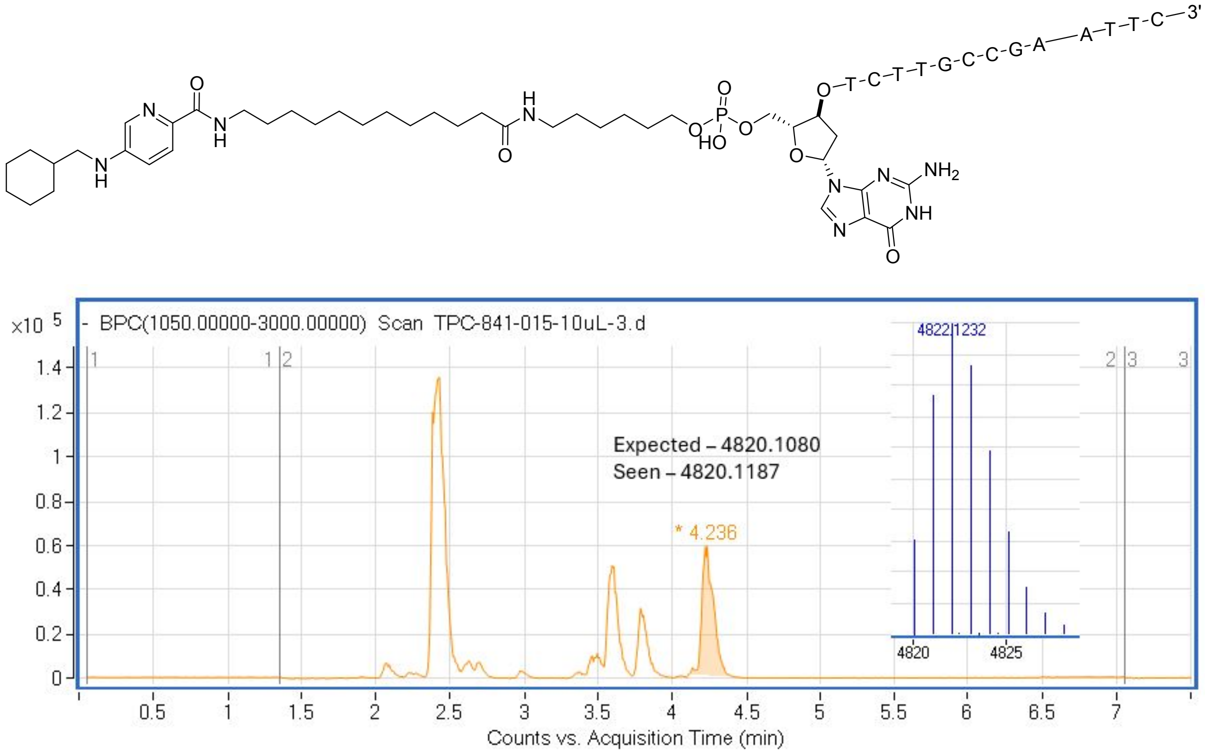

Figure S68 Chromatogram displaying double-stranded DNA product of Buchwald-Hartwig coupling between 5-iodopicolinate-conjugated DNA-greasy amine linker and piperidine.

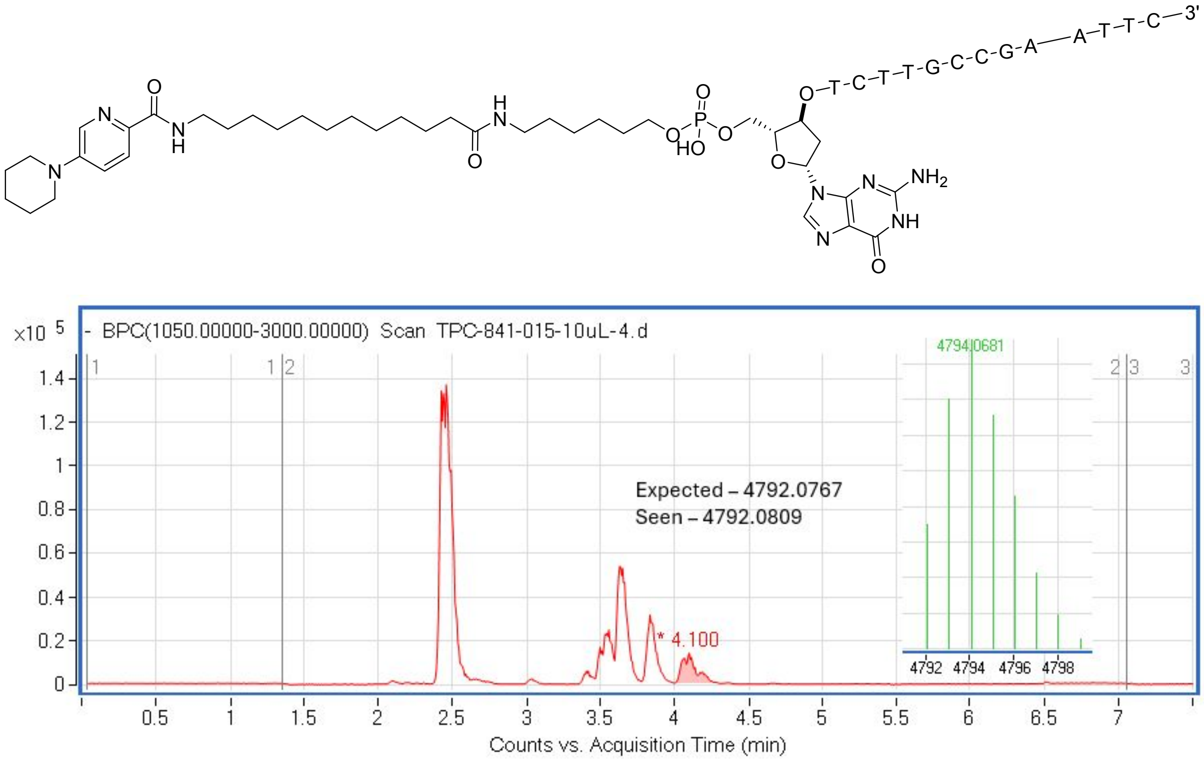

Figure S69 Chromatogram displaying double-stranded DNA product of Buchwald-Hartwig coupling between 5-iodopicolinate-conjugated DNA-greasy amine linker and *N*-methyl-1-phenylmethanamine.

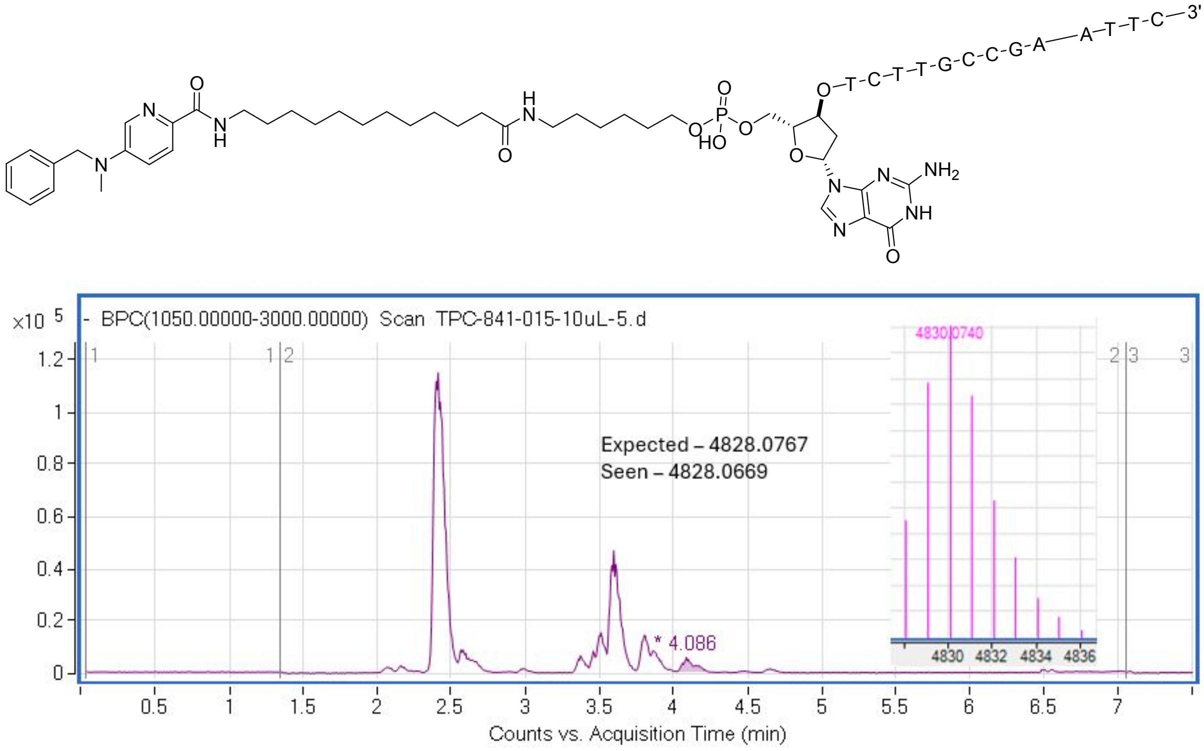

Figure S70 Chromatogram displaying double-stranded DNA product of Buchwald-Hartwig coupling between 3-iodophenyl-conjugated DNA-greasy amine linker and phenylmethanamine.

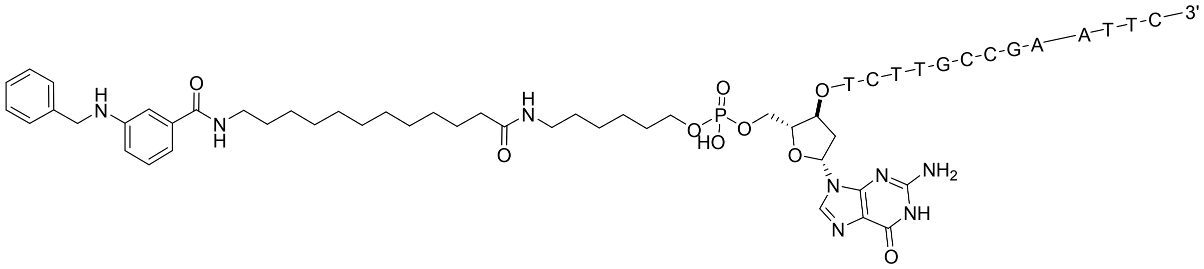

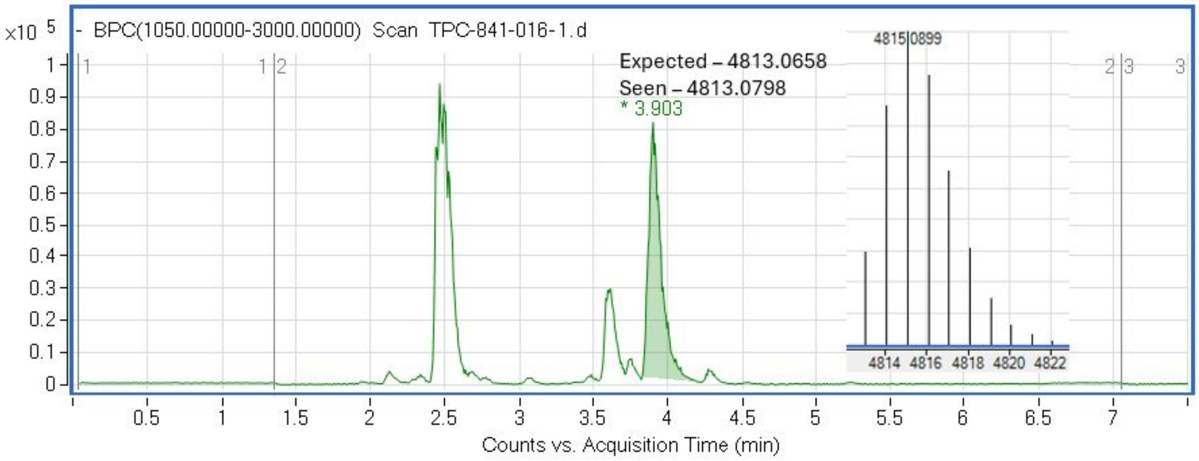

Figure S71 Chromatogram displaying double-stranded DNA product of Buchwald-Hartwig coupling between 3-iodophenyl-conjugated DNA-greasy amine linker and (2-methoxypyridin-4-yl)methanamine.

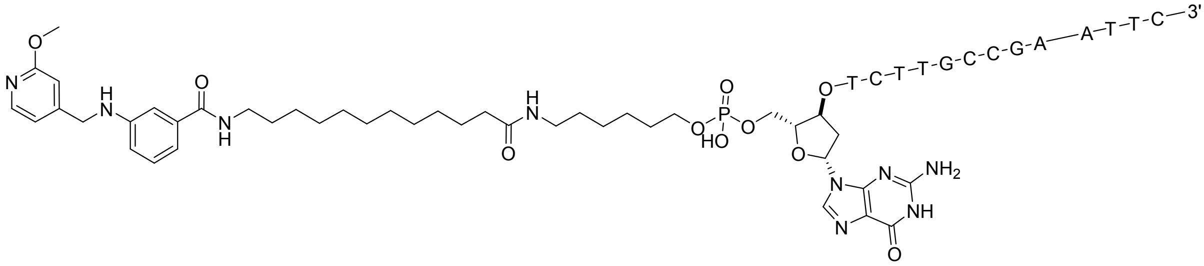

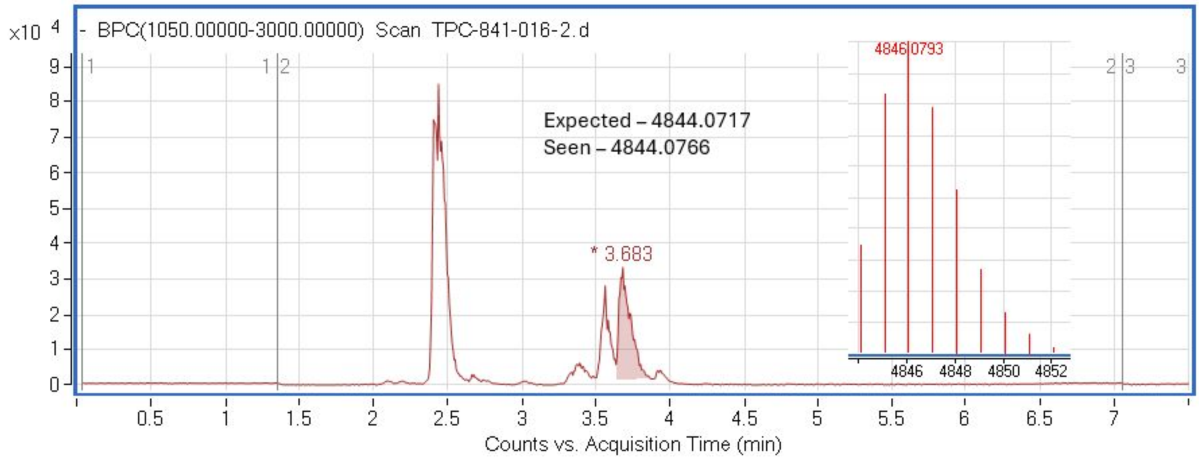

Figure S72 Chromatogram displaying double-stranded DNA product of Buchwald-Hartwig coupling between 3-iodophenyl-conjugated DNA-greasy amine linker and cyclohexylmethanamine.

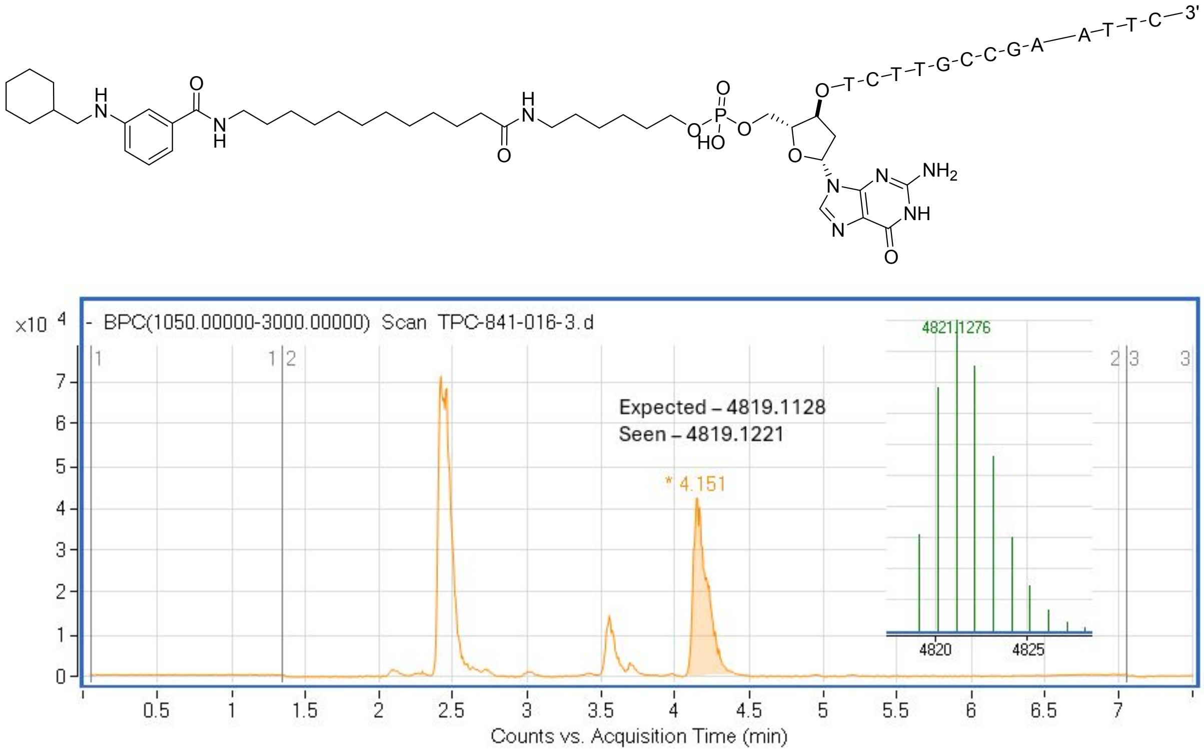

Figure S73 Chromatogram displaying double-stranded DNA product of Buchwald-Hartwig coupling between 3-iodophenyl-conjugated DNA-greasy amine linker and piperidine.

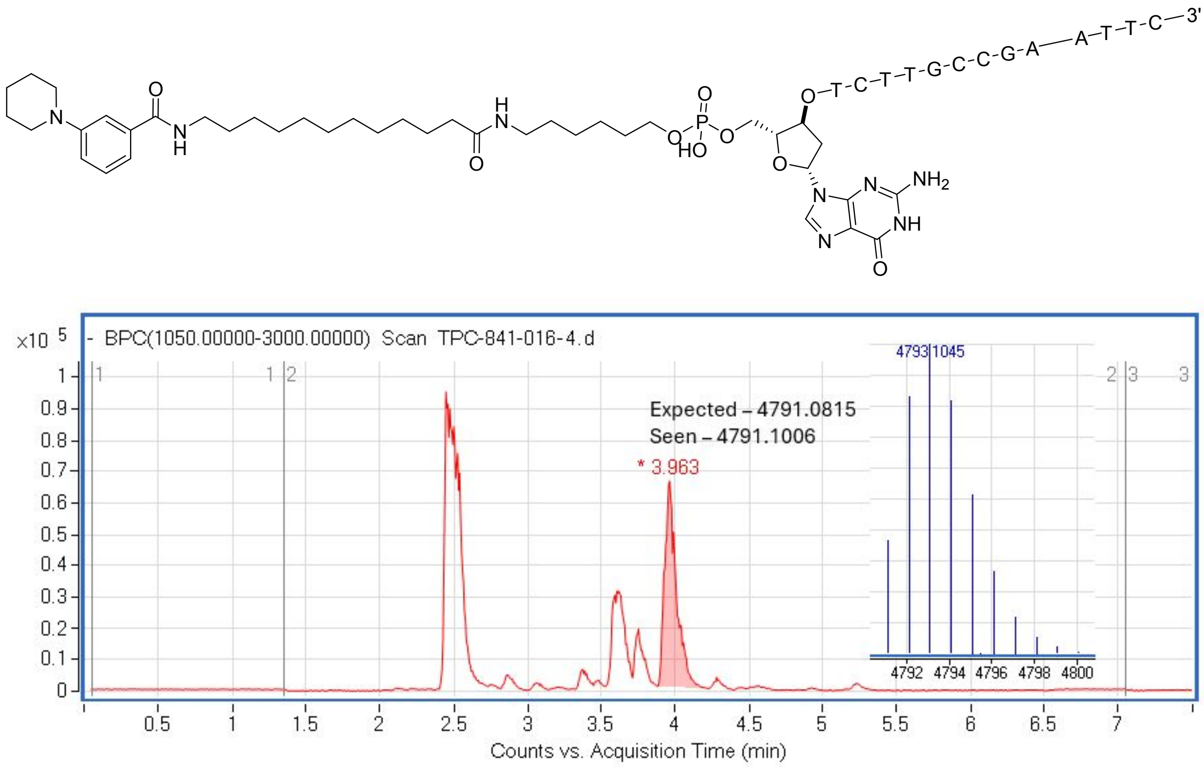

Figure S74 Chromatogram displaying double-stranded DNA product of Buchwald-Hartwig coupling between 3-iodophenyl-conjugated DNA-greasy amine linker and *N*-methyl-1-phenylmethanamine.

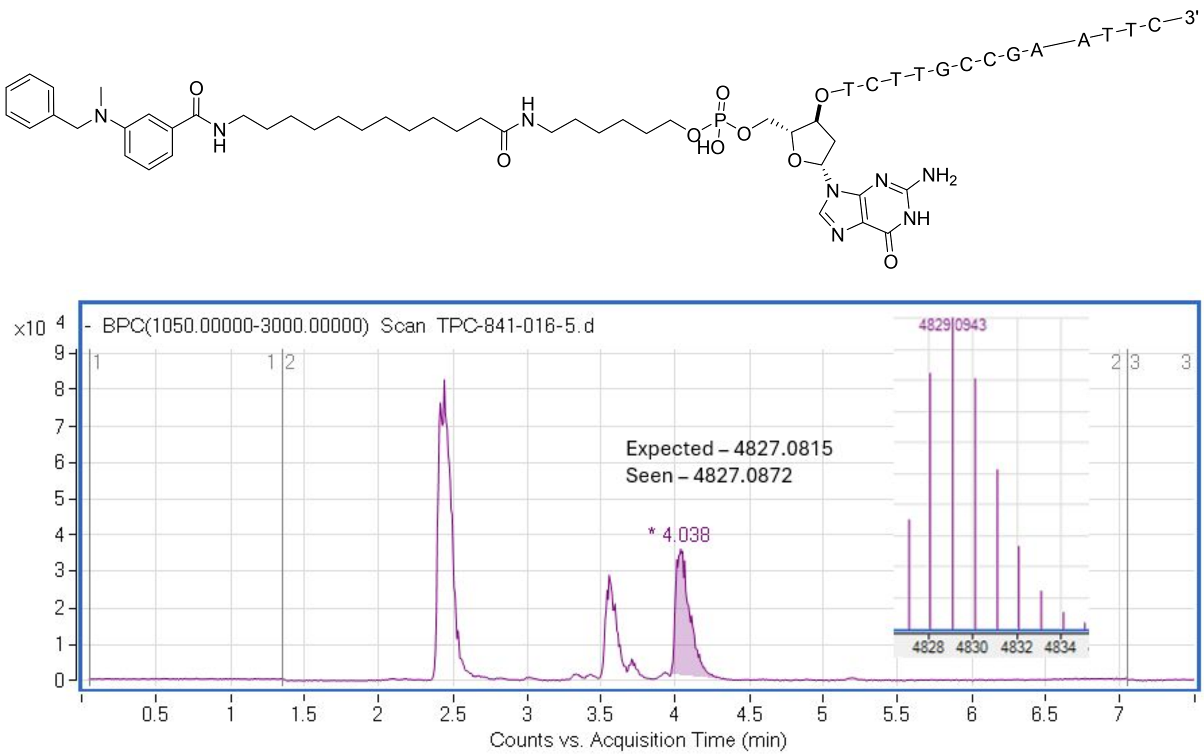

Figure S75 Chromatogram displaying double-stranded DNA product of Buchwald-Hartwig coupling between 2-(4-iodophenyl)acetate-conjugated DNA-greasy amine linker and phenylmethanamine.

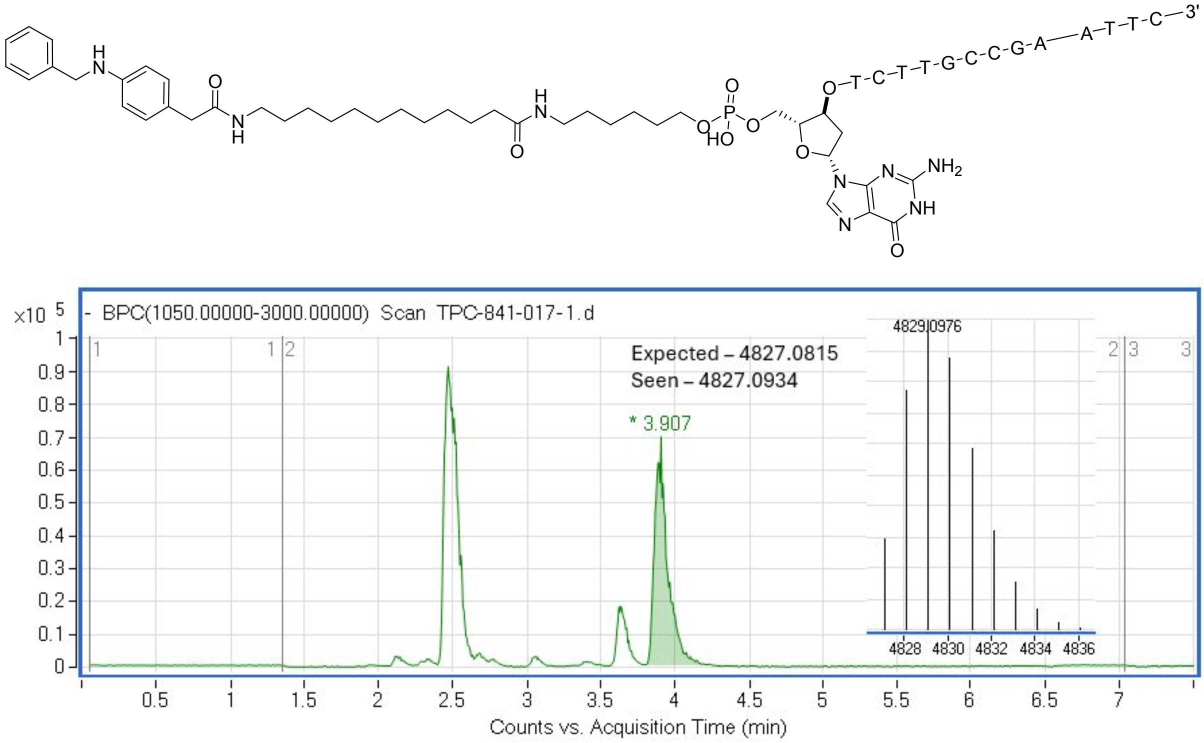

Figure S76 Chromatogram displaying double-stranded DNA product of Buchwald-Hartwig coupling between 2-(4-iodophenyl)acetate-conjugated DNA-greasy amine linker and (2-methoxypyridin-4-yl)methanamine.

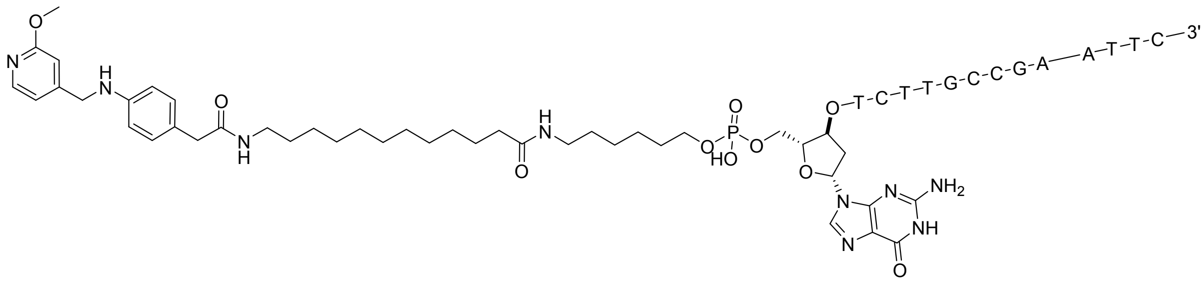

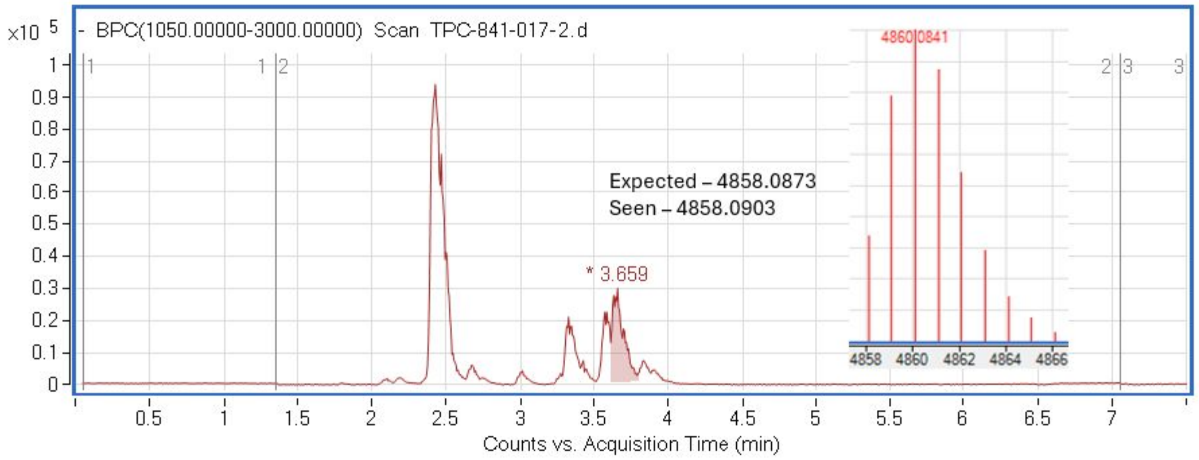

Figure S77 Chromatogram displaying double-stranded DNA product of Buchwald-Hartwig coupling between 2-(4-iodophenyl)acetate-conjugated DNA-greasy amine linker and cyclohexylmethanamine.

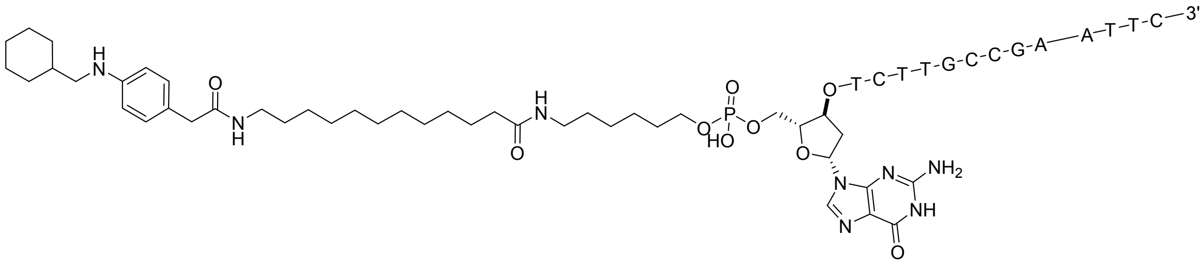

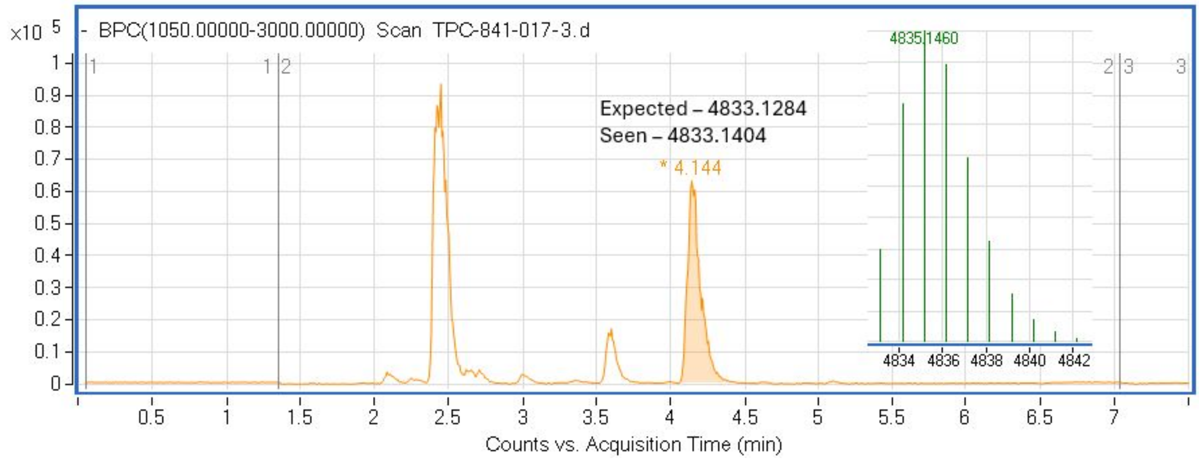

Figure S78 Chromatogram displaying double-stranded DNA product of Buchwald-Hartwig coupling between 2-(4-iodophenyl)acetate-conjugated DNA-greasy amine linker and piperidine.

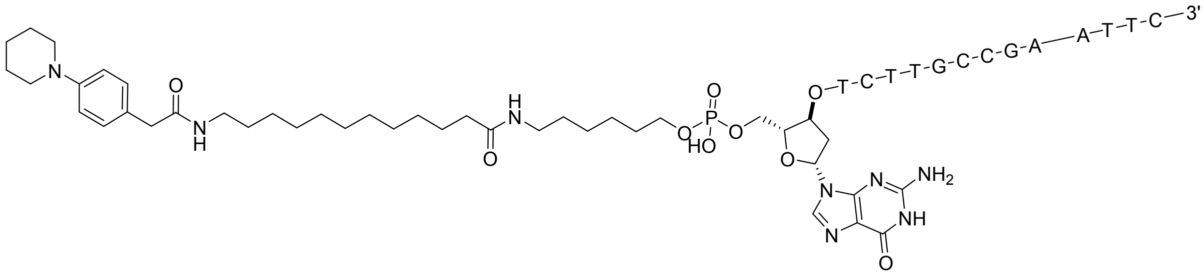

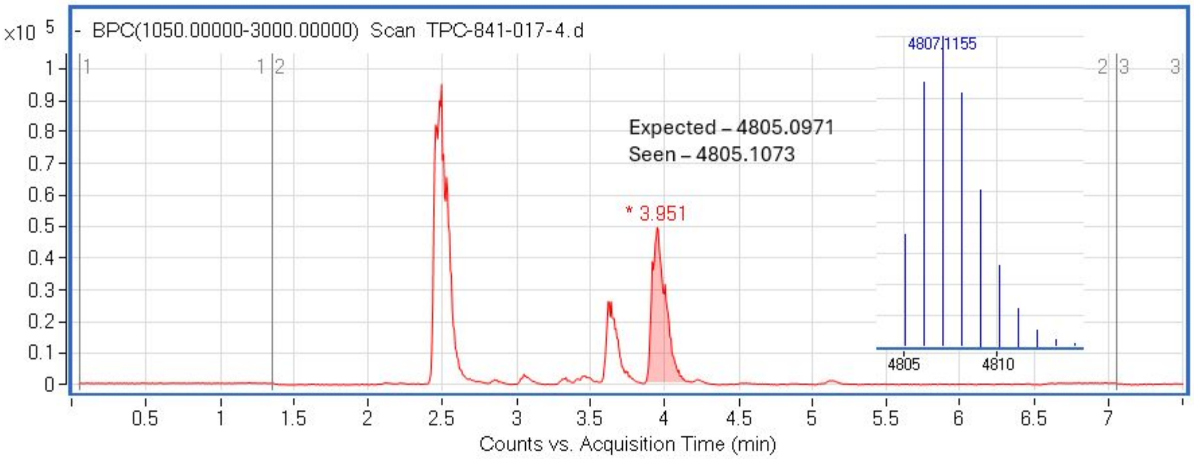

Figure S79 Chromatogram displaying double-stranded DNA product of Buchwald-Hartwig coupling between 2-(4-iodophenyl)acetate-conjugated DNA-greasy amine linker and *N*-methyl-1-phenylmethanamine.

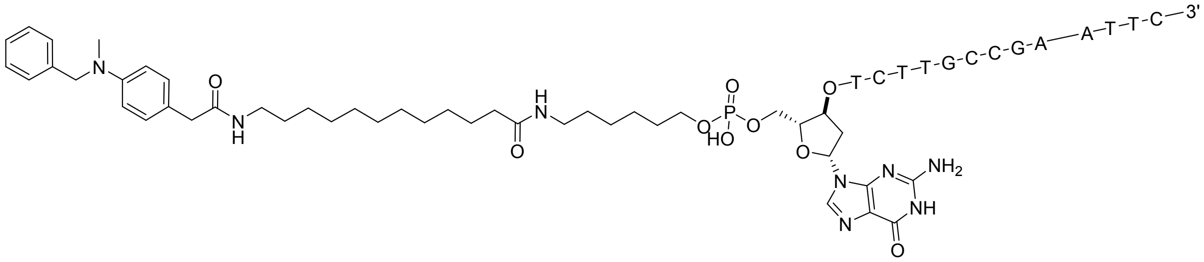

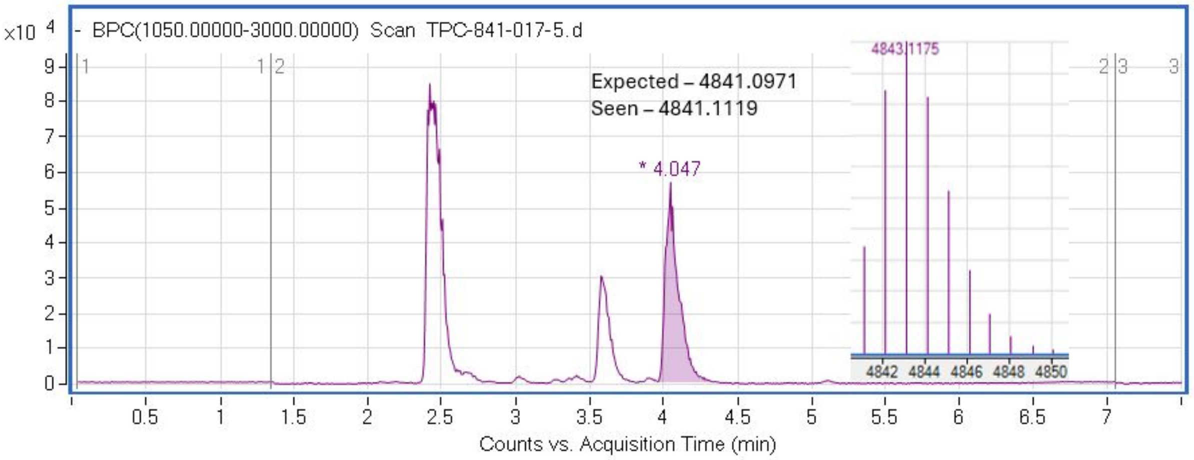

Figure S80 Chromatogram displaying double-stranded DNA product of Buchwald-Hartwig coupling 5-bromo-2-(trifluoromethyl)phenyl-conjugated DNA-greasy amine linker and phenylmethanamine.

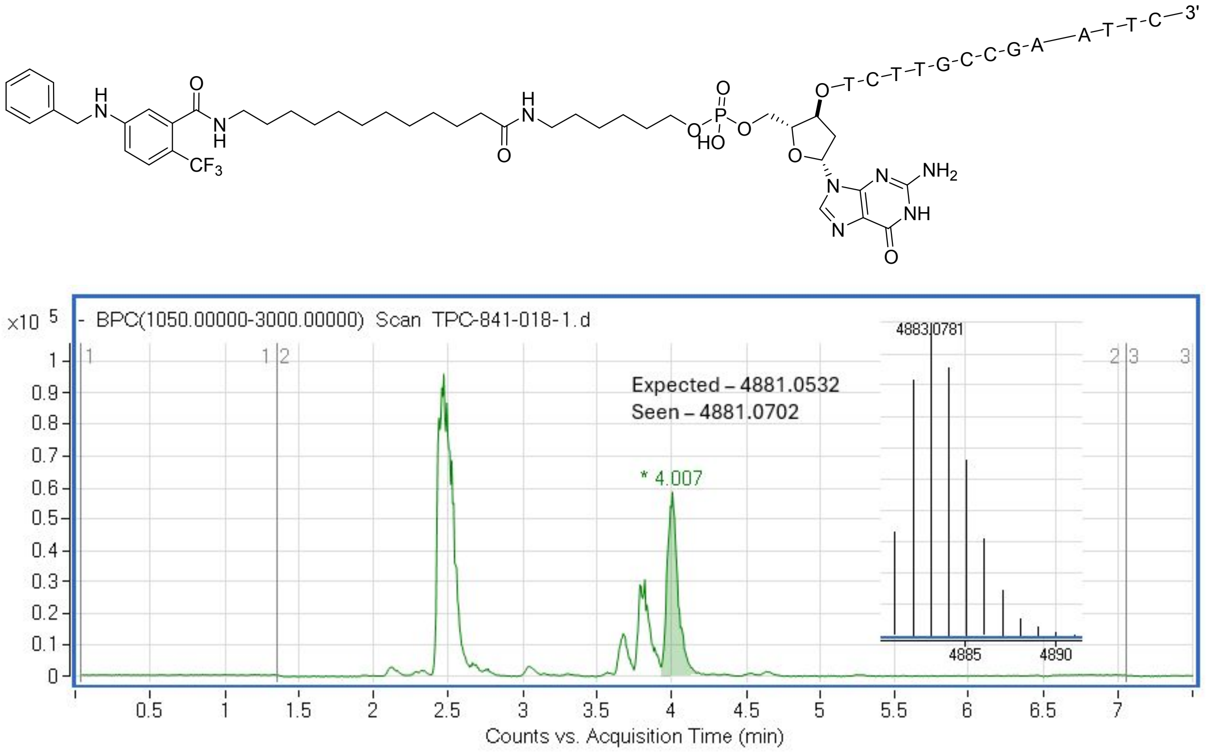

Figure S81 Chromatogram displaying double-stranded DNA product of Buchwald-Hartwig coupling 5-bromo-2-(trifluoromethyl)phenyl-conjugated DNA-greasy amine linker and (2-methoxypyridin-4-yl)methanamine.

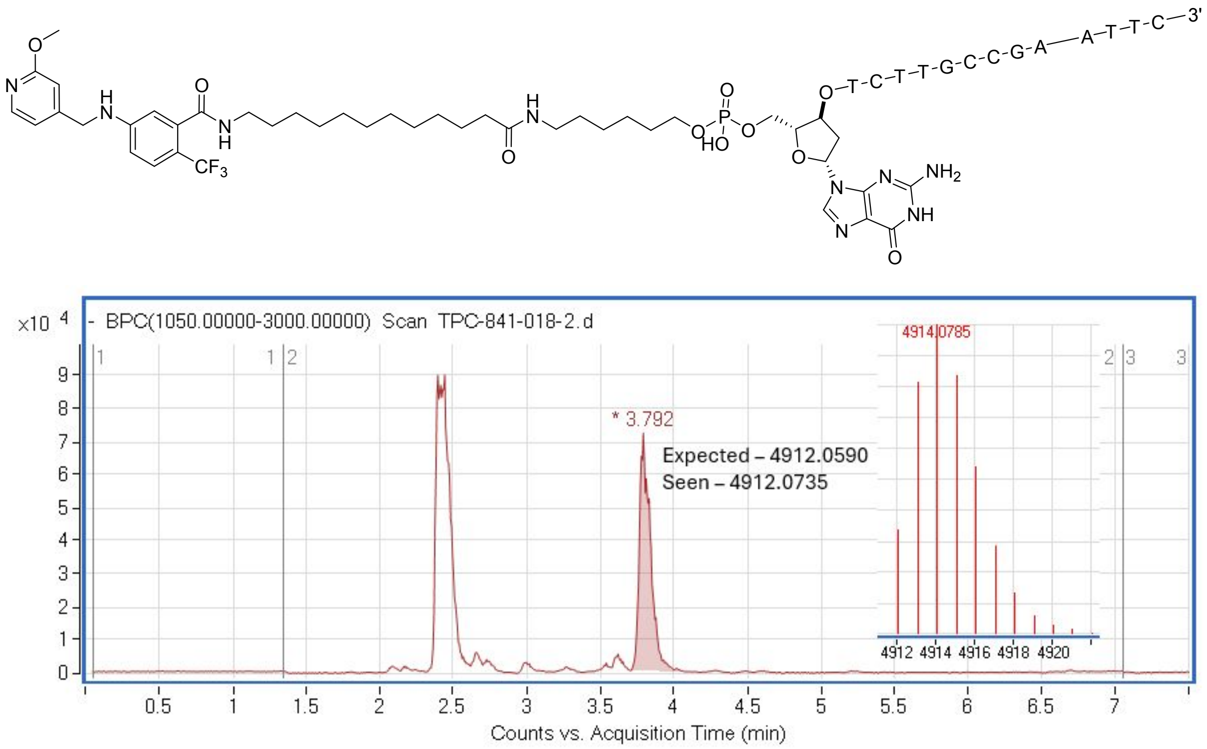

Figure S82 Chromatogram displaying double-stranded DNA product of Buchwald-Hartwig coupling 5-bromo-2-(trifluoromethyl)phenyl-conjugated DNA-greasy amine linker and cyclohexylmethanamine.

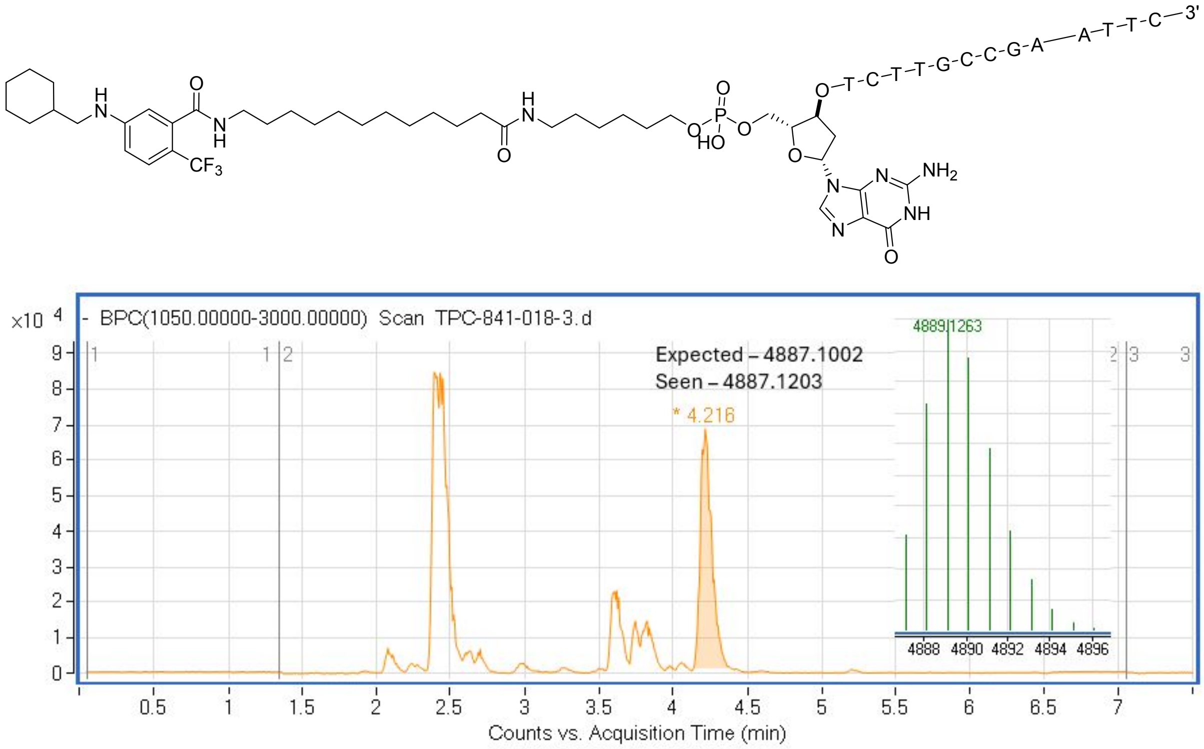

Figure S83 Chromatogram displaying double-stranded DNA product of Buchwald-Hartwig coupling 5-bromo-2-(trifluoromethyl)phenyl-conjugated DNA-greasy amine linker and piperidine.

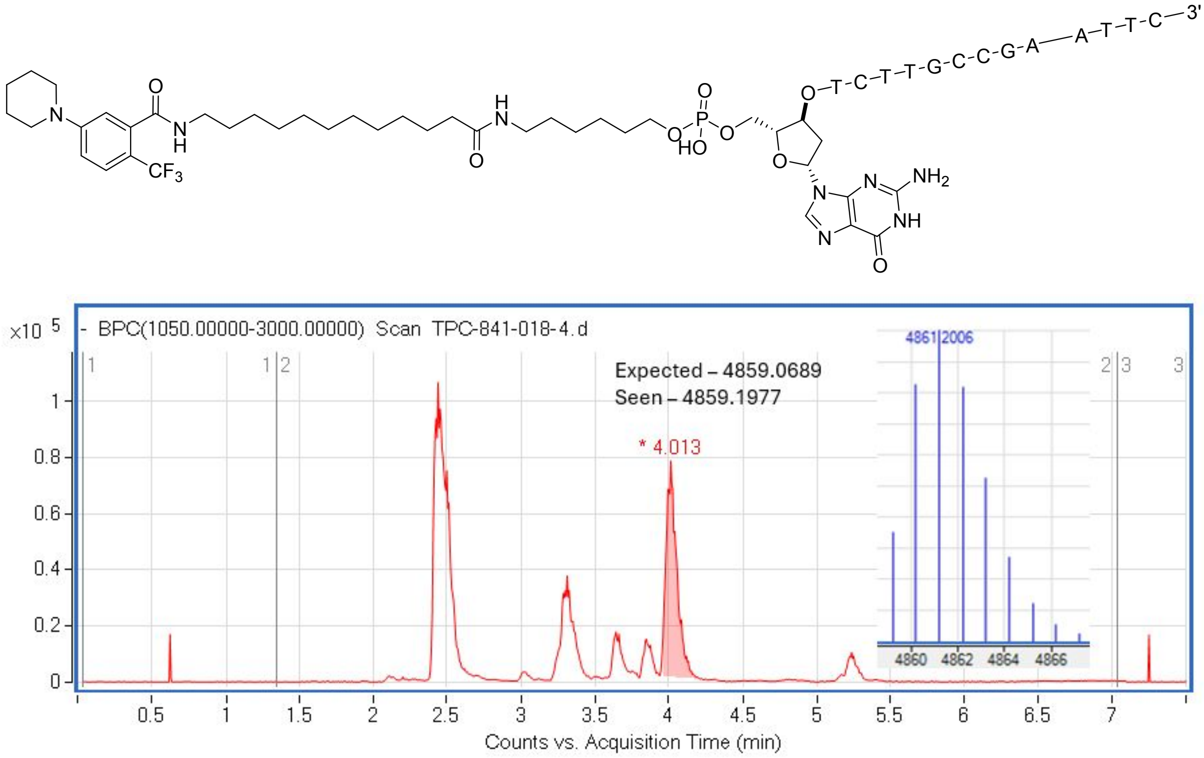

Figure S84 Chromatogram displaying double-stranded DNA product of Buchwald-Hartwig coupling 5-bromo-2-(trifluoromethyl)phenyl-conjugated DNA-greasy amine linker and *N*-methyl-1-phenylmethanamine.

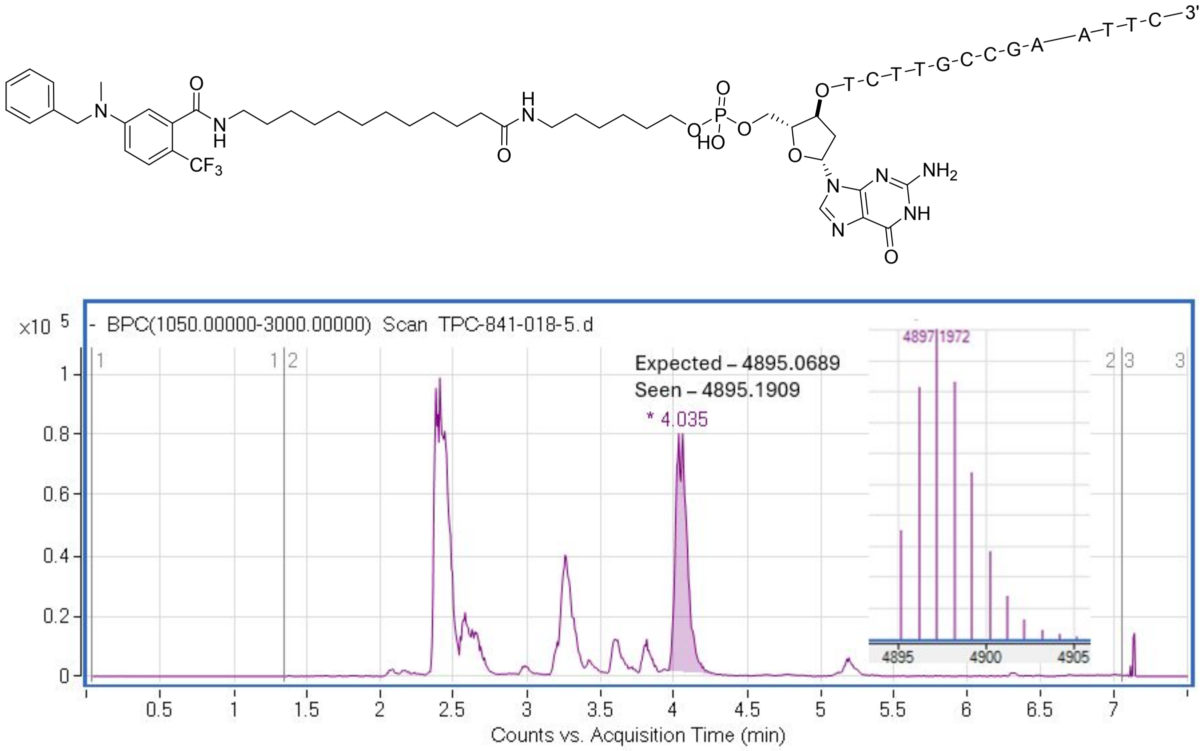

Figure S85 Chromatogram displaying double-stranded DNA product of Buchwald-Hartwig coupling 4-bromo-2-fluorophenyl-conjugated DNA-greasy amine linker and phenylmethanamine.

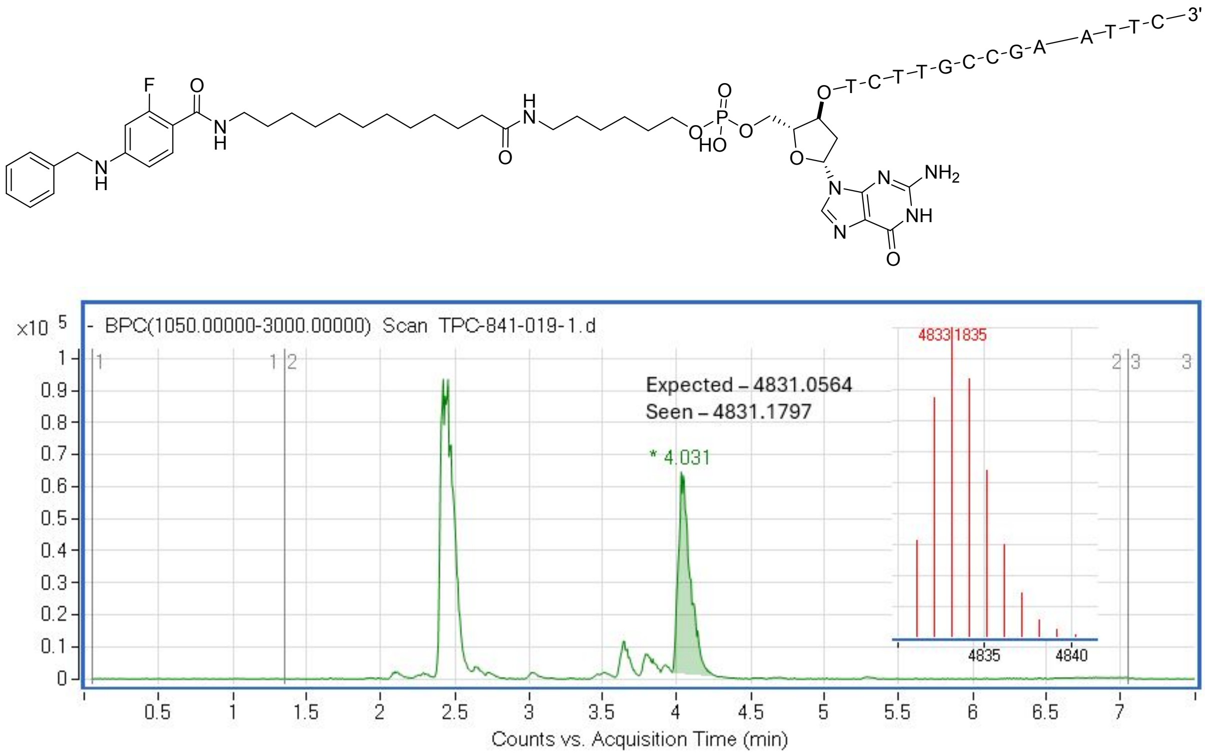

Figure S86 Chromatogram displaying double-stranded DNA product of Buchwald-Hartwig coupling 4-bromo-2-fluorophenyl-conjugated DNA-greasy amine linker and (2-methoxypyridin-4-yl)methanamine.

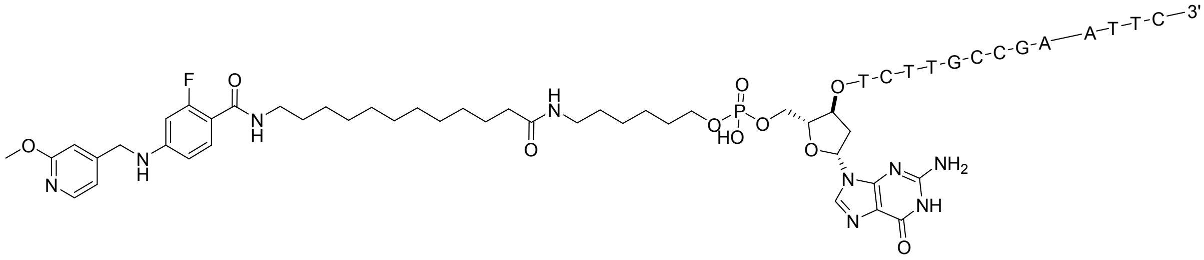

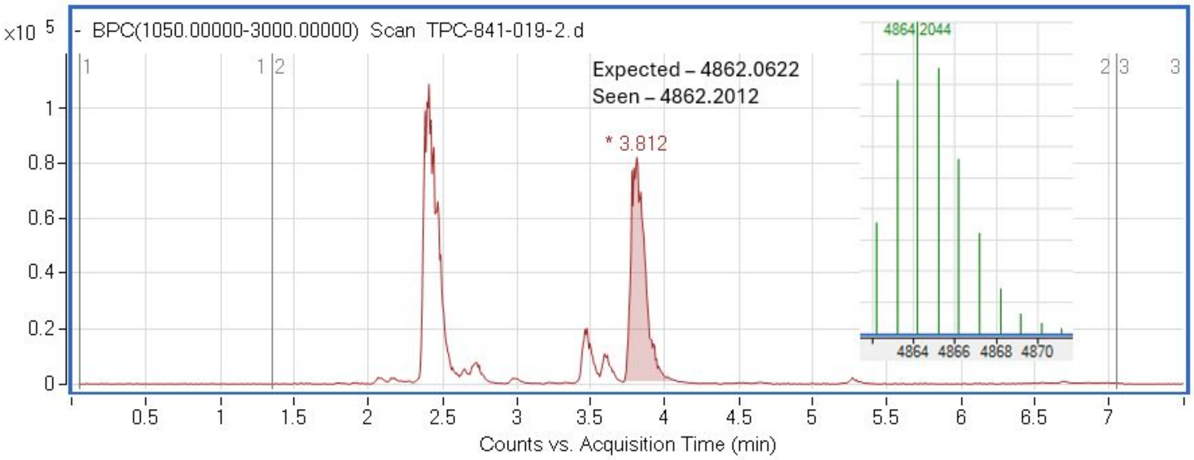

Figure S87 Chromatogram displaying double-stranded DNA product of Buchwald-Hartwig coupling 4-bromo-2-fluorophenyl-conjugated DNA-greasy amine linker and cyclohexylmethanamine.

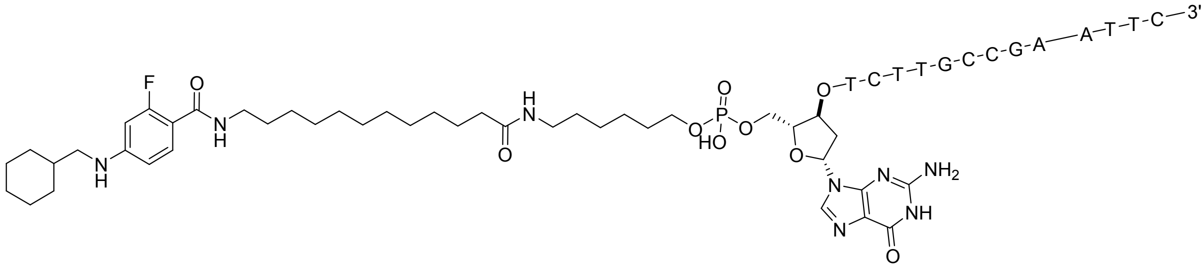

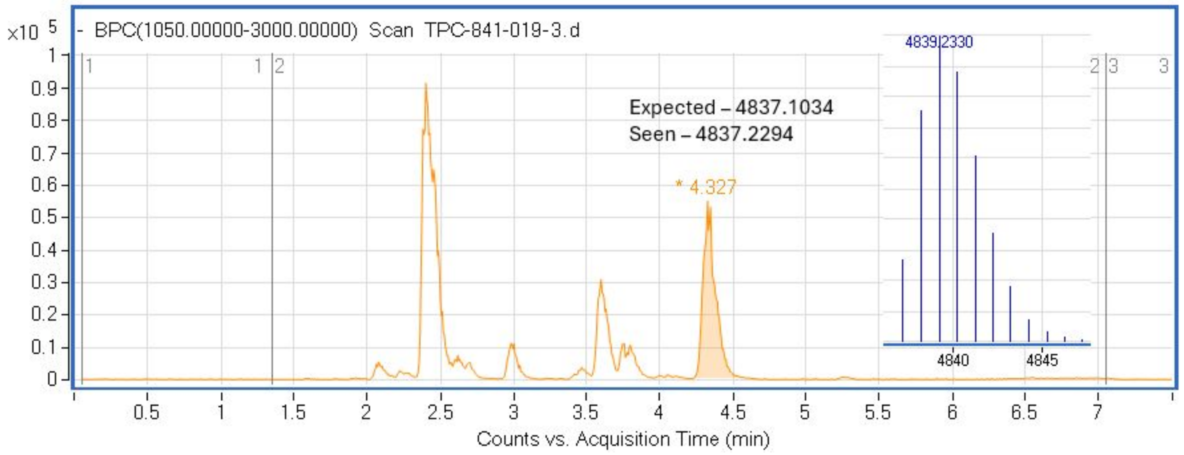

Figure S88 Chromatogram displaying double-stranded DNA product of Buchwald-Hartwig coupling 4-bromo-2-fluorophenyl-conjugated DNA-greasy amine linker and piperidine.

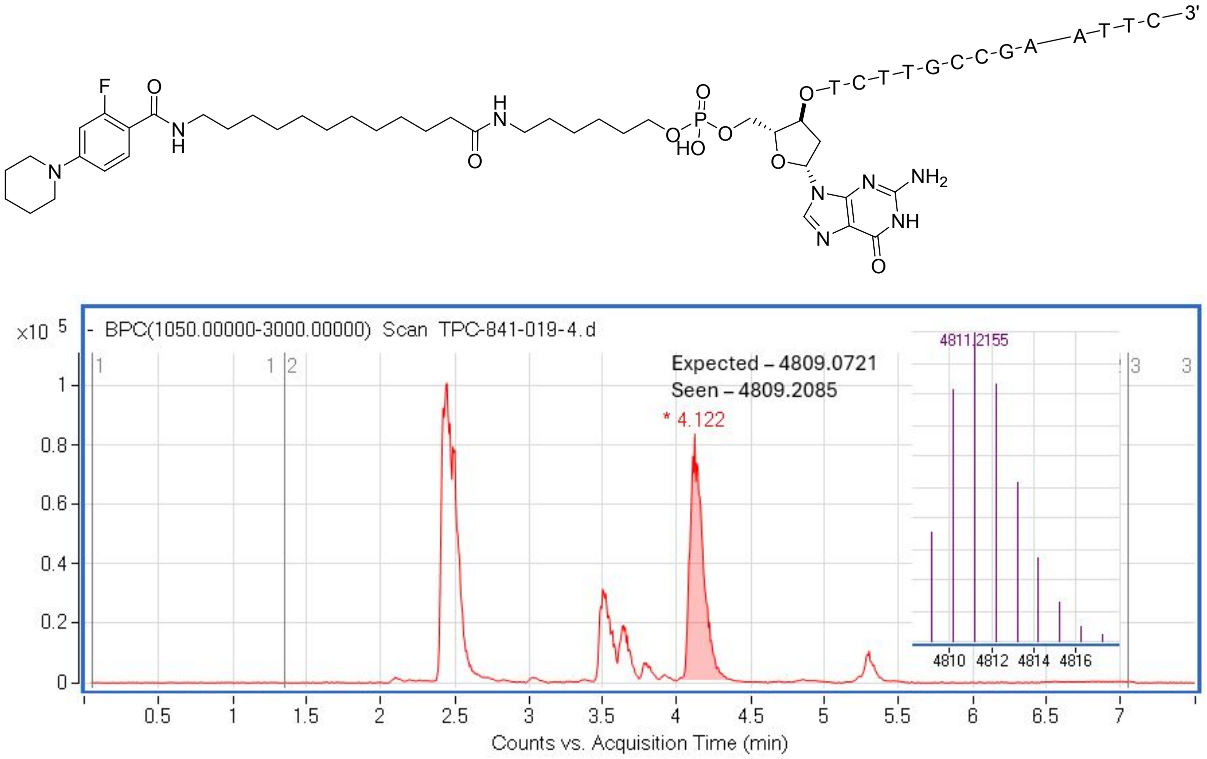

Figure S89 Chromatogram displaying double-stranded DNA product of Buchwald-Hartwig coupling 4-bromo-2-fluorophenyl-conjugated DNA-greasy amine linker and *N*-methyl-1-phenylmethanamine.

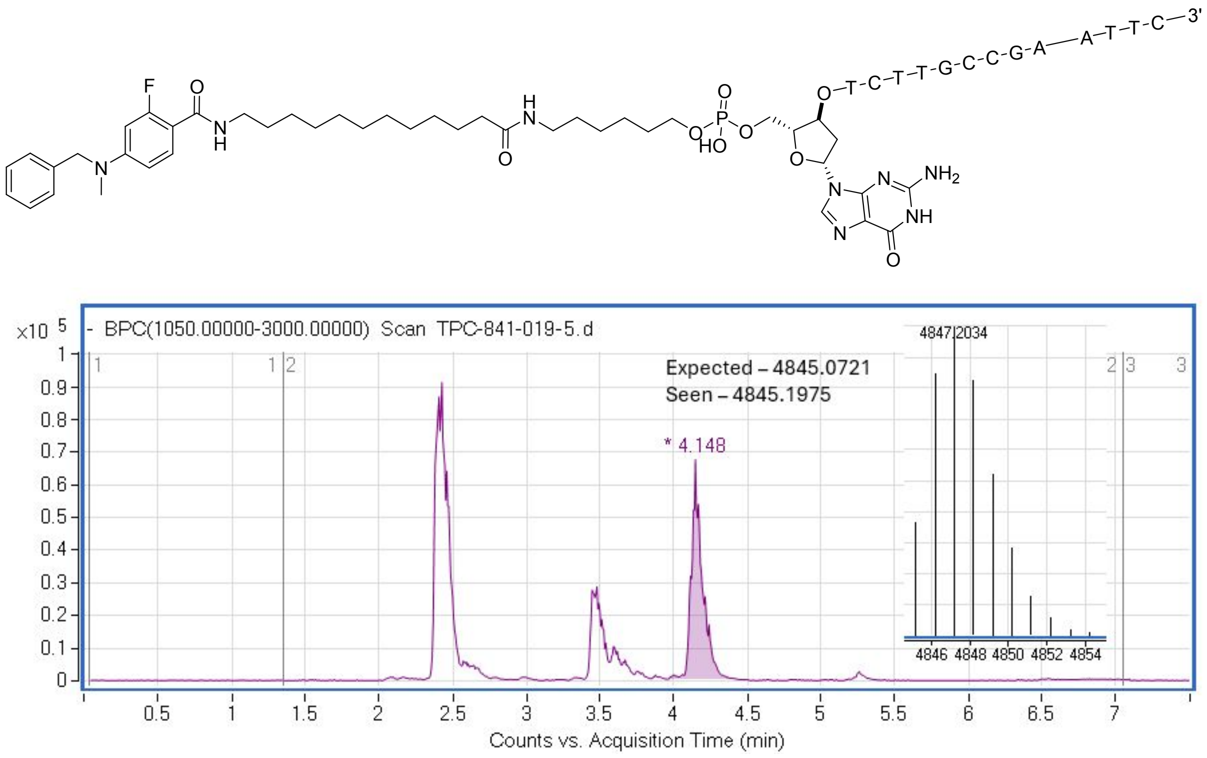

DNA-Encoded Library Synthesis

2x2 Library Construction

Scheme S2 Simplified schematic of 2x2 library synthesis. *Reagents and conditions:* i) phosphorylation (primer, complementary primer, first building block oligo), Ligation (phosphorylated DNA, headpiece oligo, first building block complementary oligo); ii) EDC, Sulfo-NHS, carboxylic acid, MOPS buffer, 37 °C, 16 h; iii) phosphorylation (library oligo + second building block), Ligation (phosphorylated DNA + complementary second building block); iv) amine, Pd(crotyl)$_2$, *t*-BuXPhos, *t*-BuOK, 5% Span 60, 70 °C, 1 h; v) phosphorylation (library oligo, third building block + complementary reverse primer oligo), Ligation (phosphorylated DNA, complementary third building block + reverse primer oligo).

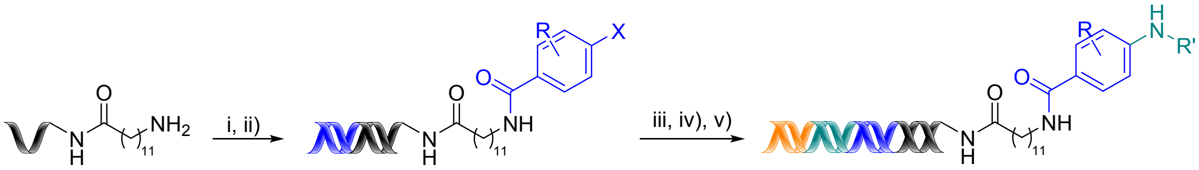

Table S1 Code abbreviations for DNA sequences utilised in 2x2 library construction.

Code	Function	Sequence (5'-3')
A	Adaptor (5' amino-linked headpiece)	GTCTTGCCGAATTC
P	Forward primer	AGGTCGGTGTGAACGGATTTG
L	Library Code	ACTATGTTAA
OH1	Overhang 1 (1st ligation)	GTAT
BB1a	Building block 1a	AGTTGGAGGA
BB1b	Building block 1b	AGATCAACCA
OH2	Overhang 2 (2nd ligation)	CCTA
BB2a	Building block 2a	TTCTAATCAA
BB2b	Building block 2b	TGTTTCGATA
OH3	Overhang 3 (3rd ligation)	TACG
BB3	Building block 3	GCGTCTCTAC
P2'	Complementary sequence to P2'	TGACCTCAACTACATGGTCTACA
A'	Complementary sequence to A	GAATTCGGCAAGAC
P'	Complementary sequence to P	CAAATCCGTTCACACCGACCT
L'	Complementary sequence to L	TTGAGCTAAT
OH1'	Complementary sequence to OH1	ATAC
BB1a'	Complementary sequence to BB1a	TCCTCCAACT
BB1b'	Complementary sequence to BB1b	TGGTTGATCT
OH2'	Complementary sequence to OH2	TAGG
BB2a'	Complementary sequence to BB2a	TTGATTAGAA
BB2b'	Complementary sequence to BB2b	TATCGAAACA
OH3'	Complementary sequence to OH3	CGTA
BB3'	Complementary sequence to BB3	GTAGAGACGC
P2'	Reverse primer	TGTAGACCATGTAGTTGAGGTCA

Figure S90 Ligation strategy for 2x2 library construction: a) complete breakdown and arrangement of DNA strands in full library member; b) overview of components added in each ligation step.

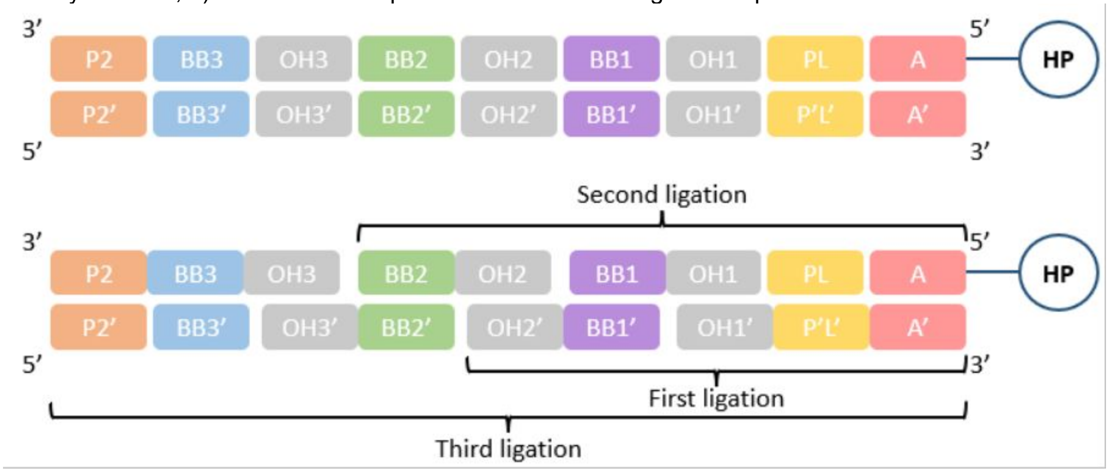

Cycle 1

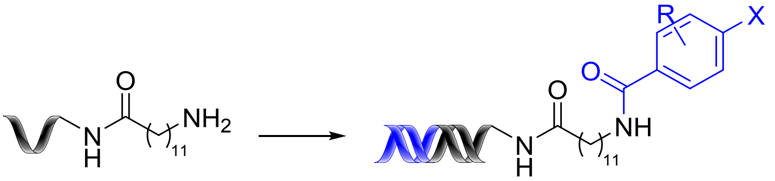

Scheme S3 First cycle of library synthesis, including ligations and chemical step (amide coupling).

In individual Applied Biosystems™ MicroAmp® 96-Well Reaction Plate wells, DNA sequences PL, BB1aOH1, and A'PL'OH1' (10 µL, 1 nmol) were combined with ATP (2 µL, 10 mM in H_2O), ThermoScientific™ 10X reaction buffer A (2 µL, 500 mM Tris-HCl [pH 7.6 at 25 °C], 100 mM $MgCl_2$, 50 mM DTT, 1 mM spermidine), T4 polynucleotide kinase (1 µL, 10 U/µL), and DEPC-treated water (5 µL). Each well was carried out in 4 repeats to yield 4 nmol of total DNA input per building block. The reactions were conducted at 37 °C for 1 h and then heated to 75 °C for 10 mins. DNA was used in subsequent ligation without further purification.

20 µL of each phosphorylation reaction was added to non-phosphorylated DNA sequences A (5 µL, 1 nmol) and BB1a'OH2' (10 µL, 1 nmol), followed by ThermoScientific™ 10X DNA ligase buffer (9 µL, 400 mM Tris-HCl, 100 mM $MgCl_2$, 100 mM DTT, 5 mM ATP), T4 DNA ligase HC (1.5 µL, 30 U/µL), and DEPC-treated water (4.5 µL). The reactions were conducted at 37 °C for 16 h and then heated to 75 °C for 10 mins. The product was then purified according to the general ethanol precipitation procedure.

The double-stranded DNA was then reacted with 4-iodobenzoic acid according to the general on-DNA EDC amide coupling procedure to yield the aqueous DNA product.

The above reactions were conducted in parallel, replacing BB1a with BB1b, BB1a' with BB1b', and 4-iodobenzoic acid with 4-bromo-2-fluorobenzoic acid, before both parallel products were combined to form 7.2 nmol of cycle 1 product (L1).

Figure S91 Gel electrophoresis visualisation of ligations utilising BB1a (1a, left) and BB1b (1b, right) showing bands ca.60 DNA base pairs in length (expected 59).

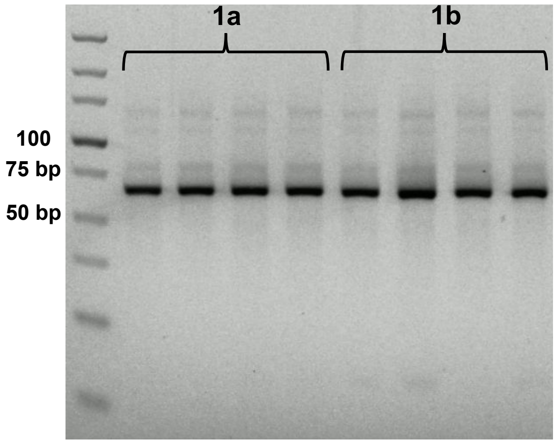

Cycle 2

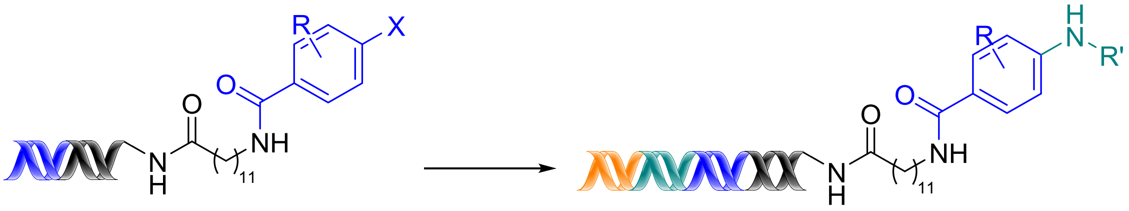

Scheme S4 Second cycle of library synthesis, including ligations and chemical step (Buchwald-Hartwig coupling).

In individual Applied Biosystems™ MicroAmp® 96-Well Reaction Plate wells, cycle 1 product L1 (10 µL, 1.8 nmol) and BB2aOH2 (10 µL, 1 nmol) were combined with ATP (2 µL, 10 mM in H_2O), ThermoScientific™ 10X reaction buffer A (2 µL), T4 polynucleotide kinase (1 µL, 10 U/µL), and DEPC-treated water (5 µL). Cycle 1 product well was carried out in 2 repeats to yield 3.6 nmol of total DNA input per building block. BB2aOH2 was carried out in 5 repeats to yield excess for subsequent ligation. The reactions were conducted at 37 °C for 1 h and then heated to 75 °C for 10 mins. DNA was used in subsequent ligation without further purification.

36 µL of the combined phosphorylation reaction of BB2aOH2 and 20 µL of the combined phosphorylation reaction of cycle 1 product (1.8 nmol) were added to non-phosphorylated DNA sequence OH3'BB2' (18 µL, 1.8 nmol), followed by ThermoScientific™ 10X DNA ligase buffer (9 µL, 400 mM Tris-HCl, 100 mM $MgCl_2$, 100 mM DTT, 5 mM ATP), T4 DNA ligase HC (1.5 µL, 30 U/µL), and DEPC-treated water (5.5 µL). The reactions were conducted at 37 °C for 16 h and then heated to 75 °C for 10 mins. The product was then purified according to the general ethanol precipitation procedure.

The double-stranded DNA was then reacted with benzylamine according to the general on-DNA Aliphatic Buchwald-Hartwig coupling procedure to yield the aqueous DNA product.

The above reactions were conducted in parallel, replacing BB2a with BB2b, BB2a' with BB2b', and benzylamine with (2-methoxypyridin-4-yl)methanamine, before both parallel products were combined to form 5.4 nmol of complete 2nd cycle (L2).

In individual Applied Biosystems™ MicroAmp® 96-Well Reaction Plate wells, cycle 2 product L2 (10 µL, 1.35 nmol) and BB3OH3P2 (10 µL, 1 nmol) were combined with ATP (2 µL, 10 mM in H_2O), ThermoScientific™ 10X reaction buffer A (2 µL, , 500 mM Tris-HCl [pH 7.6 at 25 °C], 100 mM $MgCl_2$, 50 mM DTT, 1 mM spermidine), T4 polynucleotide kinase (1 µL, 10 U/µL), and DEPC-treated water (5 µL). Cycle 2 product well was carried out in 4 repeats to yield 5.4 nmol of total DNA input. BB3OH3P2 was carried out in 8 repeats to yield excess for subsequent ligation. The reactions were conducted at 37 °C for 1 h and then heated to 75 °C for 10 mins. DNA was used in subsequent ligation without further purification.

27 µL of the combined phosphorylation reaction of BB3OH3P2 and 20 µL of the combined phosphorylation reaction of cycle 2 product (1.35 nmol) were added to non-phosphorylated DNA sequence P2'BB3' (18 µL, 1.8 nmol), followed by ThermoScientific™ 10X DNA ligase buffer (9 µL, 400 mM Tris-HCl, 100 mM $MgCl_2$, 100 mM DTT, 5 mM ATP), T4 DNA ligase HC (1.5 µL, 30 U/µL), and DEPC-treated water (19 µL). The reactions were conducted at 37 °C for 16 h and then heated to 75 °C for 10 mins. The product was then purified according to the general ethanol precipitation procedure to afford the complete 2x2 DNA-encoded library (**L3**).

Figure S92 Gel electrophoresis visualisation of ligations after combination of the complete 2x2 library, showing band ca.100 DNA base pairs in length (expected 110).

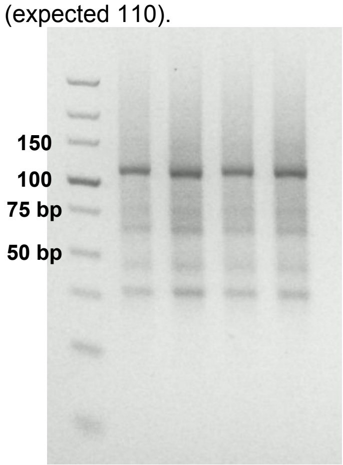

PCR and Sequencing

PCR amplification was performed in a Bio-Rad Hardshell® 96-well plate. Library DNA **L3** (1 pmol, 20 µL), combined PCR primer and reverse PCR primer (2 µL 10 µM in H_2O), DEPC-treated water (3 µL), and Applied Biosystems™ AmpliTaq Gold™ 360 Master Mix (25 µL) were combined in a single well and exposed to thermal cycling conditions consisting of 10 minutes at 95 °C, followed by 40 cycles of: 30 s at 95 °C, 30 s at 55 °C and 1 minutes at 72 °C; with a final extension time of 420 seconds at 72 °C. A negative control with DEPC-treated water replacing primer mix was ran in parallel.

The PCR products were cleaned up using NucleoSpin Gel and PCR Clean-up Columns for gel extraction and PCR clean up (Macherey-Nagel, Item number: 740609.250) (according to manufacturer guidelines). The PCR product was visualised by gel electrophoresis showing a band just above 150 base pairs (expected 161 base pairs). The DNA was diluted to 21 ng/µL and analysed by NGS (Genewiz, South Plainfield, NJ, USA), 68% of 42,222 reads corresponded to the expected sequences.

Figure S93 Gel electrophoresis visualisation post PCR, showing bands ca.150 DNA base pairs in length (expected 161).

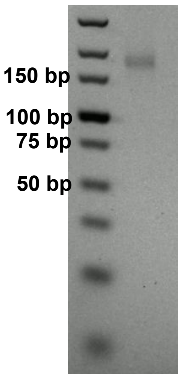

Table S2 Ten most frequent sequences and corresponding counts after NGS analysis of the PCR product (performed by Genewiz, South Plainfield, NJ, USA). 68% of ~42,222 reads correspond to the 4 expected sequences.

Sequence	Count	Relative Frequency
TGTAGACCATGTAGTTGAGGTCAGCGTCTCTACCGTATTGATTAGAATA GGTGGTTGATCTATACTTGAGCTAATCAAATCCGTTCACACCGACCT	9274	0.220
TGTAGACCATGTAGTTGAGGTCAGCGTCTCTACCGTATTGATTAGAATA GGTCCTCCAACTATACTTGAGCTAATCAAATCCGTTCACACCGACCT	8214	0.195
TGTAGACCATGTAGTTGAGGTCAGCGTCTCTACCGTATATCGAAACAT AGGTGGTTGATCTATACTTGAGCTAATCAAATCCGTTCACACCGACCT	5399	0.128
TGTAGACCATGTAGTTGAGGTCAGCGTCTCTACCGTATATCGAAACAT AGGTCCTCCAACTATACTTGAGCTAATCAAATCCGTTCACACCGACCT	5816	0.138
TGTAGACCATGTAGTTGAGGTCAGCGTCTCTACCGTATATTGAAACATA GGTGGTTGATCTATACTTGAGCTAATCAAATCCGTTCACACCGACCT	55	0.001
TGTAGACCATGTAGTTGAGGTCAGCGTCTCTACCGTATTGATTAGAATA GGTAGTTGATCTATACTTGAGCTAATCAAATCCGTTCACACCGACCT	238	0.006
TGTAGACCATGTAGTTGAGGTCAGCGTCTCTACCGTATATCGAAACAT AGGTGGTTGATTTATACTTGAGCTAATCAAATCCGTTCACACCGACCT	109	0.003
TGTAGACCATGTAGTTGAGGTCAGCGTCTCTACCGTATTGATTAGAATA GGTGATTGATCTATACTTGAGCTAATCAAATCCGTTCACACCGACCT	241	0.006
TGTAGACCATGTAGTTGAGGTCAGCGTCTCTACCGTATTGATTAGAATA GGTGGTTGATCTATACTTGAGCTAATCAAATCCGTTCACACCGACCT	256	0.006
TGTAGACCATGTAGTTGAGGTCAGCGTCTCTACCGTATATCGAAACAT AGGTCTTCCAACTATACTTGAGCTAATCAAATCCGTTCACACCGACCT	77	0.002
